# Anti-Neuroinflammatory Effects of Compounds Isolated from *Quercus acuta* Thunb. Fruits via NF-κB Signaling Inhibition in BV2 Microglia

**DOI:** 10.3390/molecules30234514

**Published:** 2025-11-22

**Authors:** Hwan Lee, Yezhi Jin, Ji-Ae Hong, Chenyang Bai, Gyoyoung Lee, Suhyeon Woo, Chi-Su Yoon, Donghyuck Bae, Dong-Sung Lee

**Affiliations:** 1 Natural Resources Laboratory, Green Bio Headquarters, Jeonnam Bio Foundation (JBF), Jangheung-gun 59338, Jeollanam-do, Republic of Korea; ghksdldi123@hanmail.net (H.L.); jiae0511@jbf.kr (J.-A.H.); 2 Research Institute of Pharmaceutical Sciences (RIPS), College of Pharmacy, Chosun University, Dong-gu, Gwangju 61452, Jeollanam-do, Republic of Korea; yezhi102@gmail.com (Y.J.); uac1660@gmail.com (C.B.); dlrydud612@naver.com (G.L.); 3 Institute of Pharmaceutical Research and Development, College of Pharmacy, Wonkwang University, Iksan 54538, Jeollabuk-do, Republic of Korea; suhyeon423@naver.com (S.W.); ycs91@wku.ac.kr (C.-S.Y.)

**Keywords:** *Quercus acuta*, natural compounds, BV2 microglia, neuro-inflammation, NF-κB

## Abstract

Aging is a major risk factor for neurodegenerative diseases in which microglia-driven neuroinflammation plays a critical role in neuronal dysfunction and disease progression. In this study, we sought to isolate bioactive metabolites from *Quercus acuta* Thunb. fruits—which have traditionally been used in oriental medicine but are chemically and pharmacologically underexplored—and evaluate their anti-neuroinflammatory potential. A total of 14 compounds were isolated from an ethanol extract of *Q. acuta* fruits through bioactivity-guided isolation, and their structures were identified by NMR spectroscopy. Notably, this study is the first to demonstrate that 3,5,7,2′,6′-pentahydroxyflavanone and 2,5-dihydroxybenzaldehyde, among the compounds isolated from *Q. acuta* fruits, exhibit significant anti-neuroinflammatory activities. Both compounds reduced the production of proinflammatory mediators, including IL-6 and TNF-α, while inhibiting the expression of iNOS and COX-2. Moreover, they significantly inhibited NF-κB activation in lipopolysaccharide-induced BV2 microglia. Collectively, these findings indicate that *Q. acuta* fruits contain bioactive constituents with previously unreported anti-neuroinflammatory properties, supporting their potential as a natural source for the development of therapeutic agents targeting age-related neuroinflammation.

## 1. Introduction

Aging is a major risk factor for neurodegenerative disorders, including Alzheimer’s and Parkinson’s diseases. Among the multiple pathological mechanisms, neuroinflammation has emerged as a critical contributor to neuronal dysfunction and disease progression [1,2]. Activated microglia, the resident immune cells of the central nervous system, release proinflammatory cytokines and mediators that exacerbate neuronal damage. Suppressing microglial activation is therefore considered an important strategy for mitigating neuroinflammatory processes [3,4].

BV2 microglia are widely used as a model for studying neuroinflammation due to their stable growth characteristics and reproducible responses [5,6,7]. Upon lipopolysaccharide (LPS) stimulation, these cells induce activation of the nuclear factor kappa B (NF-κB) signaling pathway, leading to the upregulation of inflammatory mediators such as cytokines and enzymes [8,9]. Specifically, LPS-induced BV2 cells produce nitric oxide (NO) through the induction of inducible nitric oxide synthase (iNOS), while releasing proinflammatory cytokines, including interleukin-6 (IL-6) and tumor necrosis factor-alpha (TNF-α). These mediators are tightly regulated by NF-κB signaling and play a crucial role in increasing neuroinflammation; therefore, they are widely recognized biomarkers of microglial activation [10,11,12]. Consequently, the LPS-induced BV2 cell model provides a reliable experimental system for assessing whether candidate compounds can suppress NF-κB mediated inflammatory pathways.

*Quercus acuta* Thunb. is an evergreen broadleaf tree native to East Asia, including countries like the Republic of Korea, Japan, and China. In the Republic of Korea, *Q. acuta* fruits, acorns, are traditionally consumed as acorn jelly. Oriental Korean medical texts, such as the *Donguibogam*, describe *Q. acuta* fruits (referred to as “myeonja”) as possessing blood-cleansing, diuretic, analgesic, and detoxifying properties, and note their use in treating conditions such as neuralgia and joint pain. Recently, *Q. acuta* has attracted attention for its high carbon storage efficiency compared with other Fagaceae plants, leading to expanded cultivation in the southern territories of the Republic of Korea in response to climate change [13]. However, aside from ecological studies and limited applications in food sectors, most research has focused on *Q. acuta* leaves, which have been reported to exhibit anti-neuroinflammatory, antiviral, antibacterial, and antihyperuricemic effects [14,15,16,17]. Furthermore, while previous studies on *Q. acuta* in relation to neuroinflammation have primarily focused on the methanol extract of the stem, this study investigates individual bioactive compounds isolated from the fruit [14]. Studies on this fruit have suggested antiphotoaging activity, but their chemical constituents and potential anti-neuroinflammatory effects remain largely unexplored [18].

This study aimed to isolate and identify bioactive compounds from *Q. acuta* fruits and evaluate their anti-neuroinflammatory activity in LPS-induced BV2 microglia, focusing on NF-κB-mediated signaling pathways implicated in aging-related neurodegeneration. Furthermore, this study was conducted to explore the potential of *Q. acuta* as a sustainable natural resource that contributes to ecological balance through carbon storage that may provide bioactive compounds relevant to aging-related neuroinflammation, thereby supporting both natural and human antiaging processes.

## 2. Results

### 2.1. Effects of Q. acuta Fruit Ethanol Extract on Cytotoxicity and Nitrite Production

**Figure 1:**
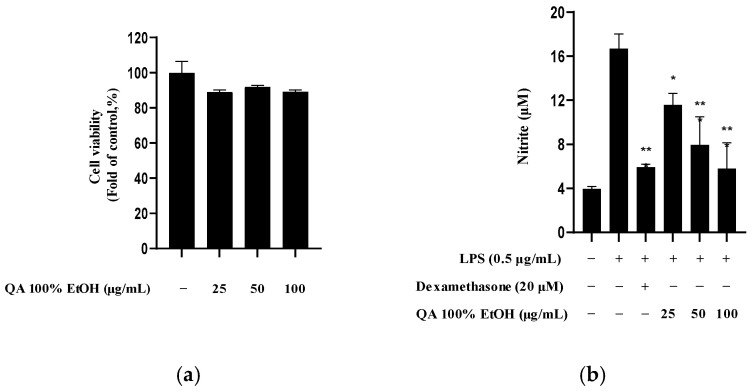
Effect of *Q. acuta* fruit EtOH extract on (**a**) cell viability and (**b**) inhibition of nitrite production in BV2 cells. (**a**) Cell viability was measured using the MTT assay after incubating cells with the indicated concentrations of *Q. acuta* fruit EtOH extract for 24 h. (**b**) The inhibitory effect on nitrite production was measured 3 h after *Q. acuta* fruit EtOH extract treatment and 24 h after LPS (0.5 μg/mL) stimulation; 20 μM dexamethasone was used as the positive control. Data are presented as the mean ± S.D. of three independent experiments (*n* = 3). * *p* < 0.05, and ** *p* < 0.01 vs. LPS-treated cells.

Since the purpose of this study was to identify the active ingredients in *Q. acuta* acorns, it was essential to determine whether the extract itself exhibited anti-neuroinflammatory effects. Therefore, we investigated the effects of the *Q. acuta* fruit ethanol (EtOH) extract in BV2 microglia. First, we performed a toxicity evaluation of the extract. No significant cytotoxicity was observed at concentrations up to 100 μg/mL (Figure 1a). We then examined LPS-induced nitrite production in BV2 microglia under non-toxic conditions, testing three extract concentrations (25, 50, and 100 μg/mL). The EtOH extract of *Q. acuta* significantly reduced nitrite production in a concentration-dependent manner (Figure 1b).

### 2.2. UHPLC-HR-MS/MS-Based Metabolite Profiling of Q. acuta Fruit Ethanol Extract

**Figure 2:**
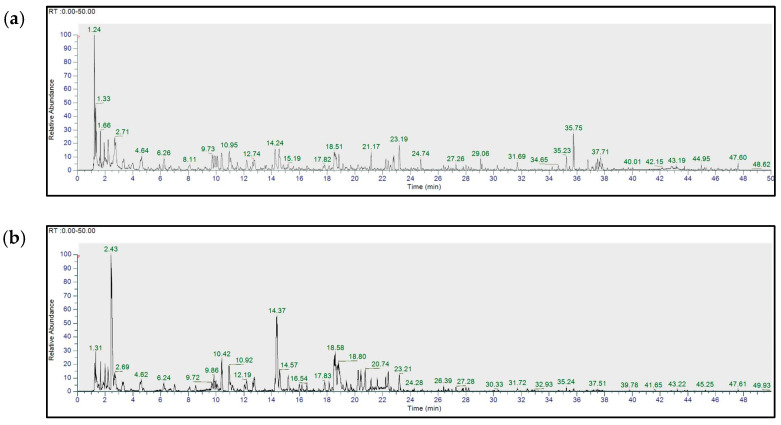
Total ion current (TIC) UPHLC-HR-MS/MS chromatograms of an EtOH extract of *Q. acuta* fruits obtained in both (**a**) positive and (**b**) negative ion modes. Identified peaks were sequentially numbered by retention time using “Elements viewer” (the identity or presumed identity of each peak is presented in Table 1, along with the experimental mass, MS/MS fragment, and identification reference).

Metabolite profiling of the EtOH extract of *Q. acuta* fruits was performed using UHPLC-HR-MS/MS. The total ion current (TIC) chromatogram of the extract detected multiple peaks distributed across a retention time range of 0–50 min, suggesting the presence of various metabolites in both positive and negative mode chromatograms (Figure 2). Tentative annotations of the detected metabolites were predicted through MS/MS fragment analysis and comparison with databases and references [19,20,21,22,23,24,25,26,27,28,29,30,31,32,33,34].

The analysis revealed that the EtOH extract of *Q. acuta* acorns comprised a variety of compounds, including phenols, coumarins, flavonoids, and tannins (Table 1). These results provided a clear basis for further separation of the *Q. acuta* EtOH extract into fractions of different polarities using a solvent partitioning method.

### 2.3. Bioactivity of Q. acuta Fruit EtOH Extract Fractions

**Figure 3:**
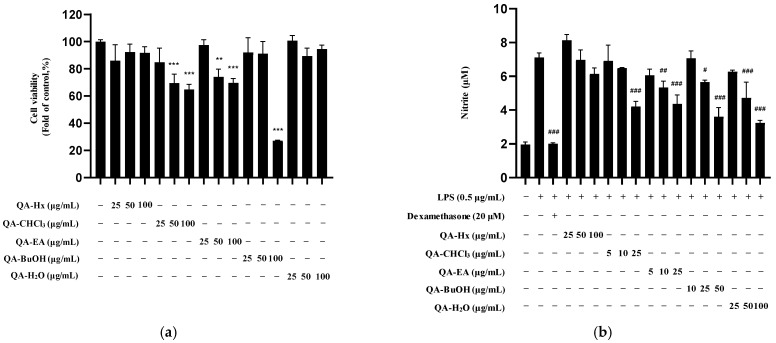

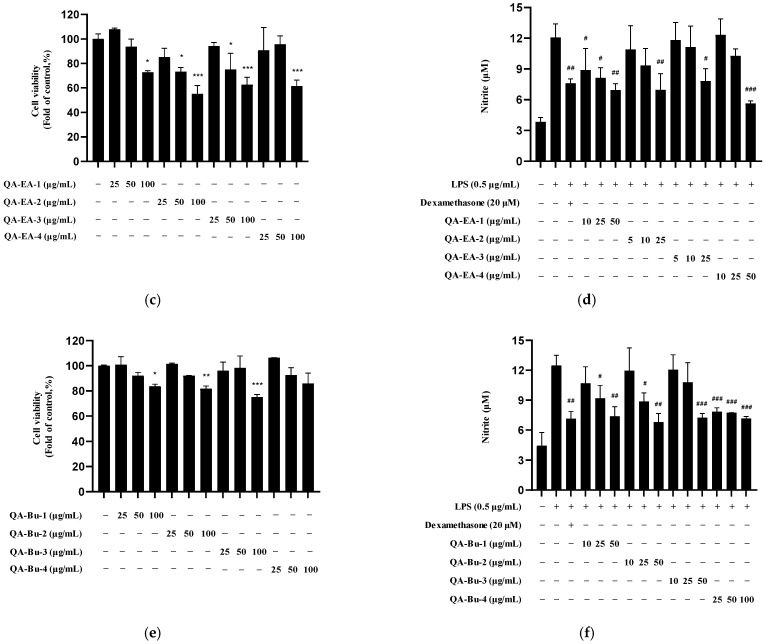
Cell viability and LPS-induced nitrite production in BV2 microglia in the presence of solvent fractions and subfractions of a *Q. acuta* fruit extract. (**a**,**c**,**e**) Cell viability of BV2 microglia treated with five solvent fractions (*n*-hexane, chloroform, ethyl acetate, *n*-butanol, and H_2_O) and ethyl acetate (QA-EA-1–4) and *n*-butanol (QA-Bu-1–4) subfractions was assessed by the MTT assay after 24 h of treatment. (**b**,**d**,**f**) Inhibitory effects of five solvent fractions (*n*-hexane, chloroform, ethyl acetate, *n*-butanol, and H_2_O) and ethyl acetate (QA-EA-1–4) and *n*-butanol (QA-Bu-1–4) subfractions on LPS-induced nitrite production in BV2 microglia after a 3 h treatment with the fractions and subfractions followed by 24 h of LPS (0.5 μg/mL) stimulation. Dexamethasone (20 μM) was used as the positive control. Data are presented as the mean ± S.D. of three independent experiments (*n* = 3). * *p* < 0.05, ** *p* < 0.01, and *** *p* < 0.001 vs. the control. ^#^ *p* < 0.05, ^##^ *p* < 0.01, and ^###^ *p* < 0.001 vs. the LPS-treated group.

To isolate anti-neuroinflammatory compounds, we examined the inhibitory effects of each solvent fraction (*n*-hexane [Hx], chloroform [CF], ethyl acetate [EA], *n*-butanol [Bu], and H_2_O [DW]) on nitrite production. To determine the optimal concentration for sample processing, we first examined the cell viability of each solvent fraction in BV2 microglia using the MTT assay (Figure 3a). Based on the results, we investigated the inhibitory effects of each fraction at non-toxic concentrations on LPS-induced nitrite production in BV2 microglia. The CF, EA, Bu, and DW fractions significantly reduced nitrite production, whereas the Hx fraction showed no significant inhibitory effect (Figure 3b).

Since HPLC analyses revealed multiple chromatographic peaks in the EA and Bu fractions, such fractions were further fractionated by C18 reversed-phase chromatography (QA-EA-1–4 and Bu-1–4), and the treatment concentrations of the fractions were determined based on the corresponding cell viability results (Figure 3c,e). Subsequently, EA and Bu subfractions were used to treat LPS-induced BV2 cells, and they all consistently inhibited nitrite production, suggesting that multiple active compounds contribute to the anti-neuroinflammatory activity of *Q. acuta* fruits (Figure 3d,f).

### 2.4. Isolation and Structural Identification of Compounds

**Figure 4:**
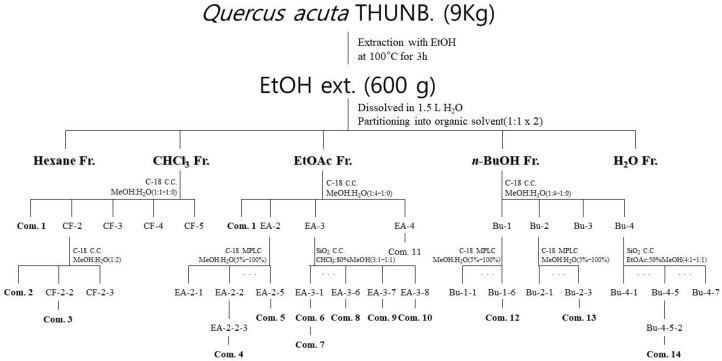
Schematic representation of the pathway followed for compound isolation from a *Q. acuta* fruits.

Guided by the nitrite inhibition effect and HPLC chromatogram results (Figure 3 and Figure S1), a total of 14 pure compounds were isolated from the CF, EA, and Bu fractions of the *Q. acuta* fruit extract (Figure 4).

**Figure 5:**
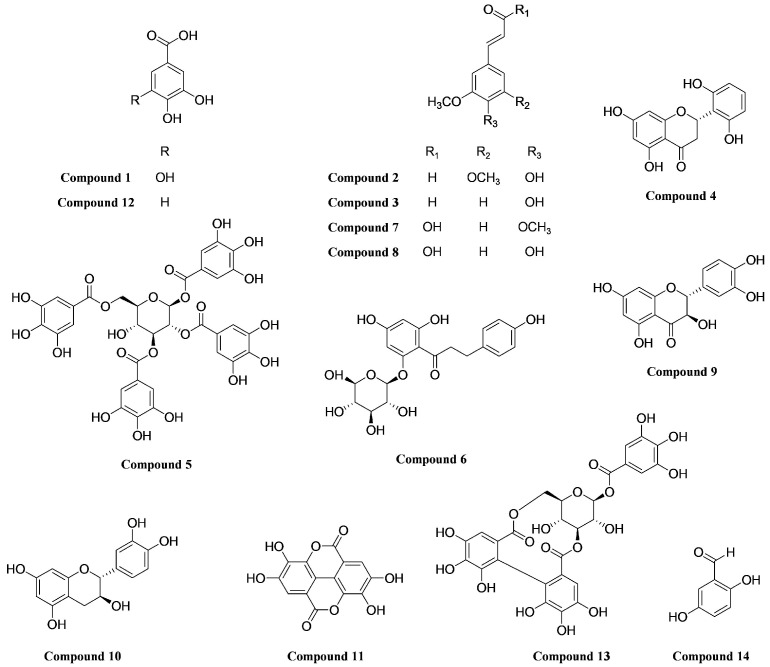
Chemical structures of the compounds isolated from a *Q. acuta* fruit EtOH extract.

The chemical structures of the isolated compounds were determined through 1D and 2D NMR spectroscopic analyses and comparison with published spectral data (Figure 5).

### 2.5. Nitrite Inhibition by Isolated Compounds

**Figure 6:**
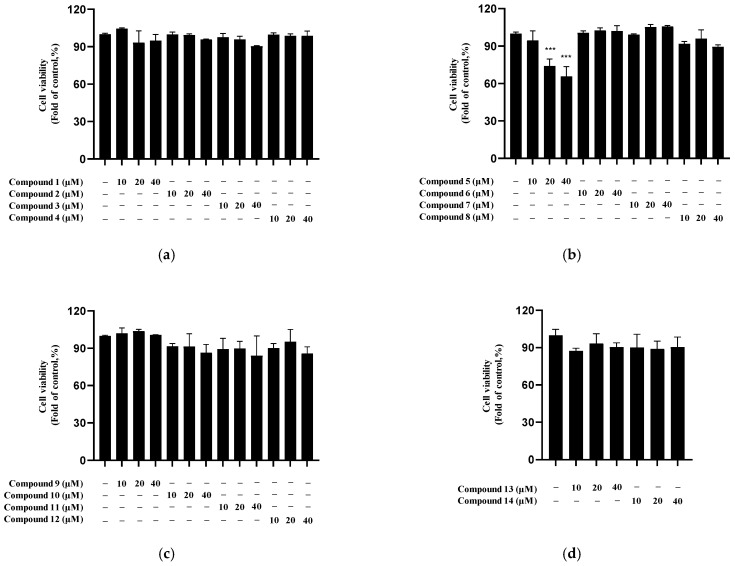
Viability of BV2 microglial cells treated with *Q. acuta* fruit compounds **1**–**14**. The subfigures represent the following groups: compounds **1**–**4** (**a**), **5**–**8** (**b**), **9**–**12** (**c**), and **13**, **14** (**d**). Cells were incubated with the indicated concentrations of *Q. acuta* fruit compounds for 24 h. Cell viability was determined by MTT assay. Data are presented as the mean ± S.D of three independent experiments (*n* = 3). *** *p* < 0.001 vs. the control.

The cytotoxicity of the 14 isolated compounds was evaluated in BV2 microglia at concentrations up to 40 μM (Figure 6). Among the tested compounds, only compound **5** exhibited cytotoxicity at 20 and 40 μM, whereas the other compounds showed no significant toxicity under the experimental conditions.

**Figure 7:**
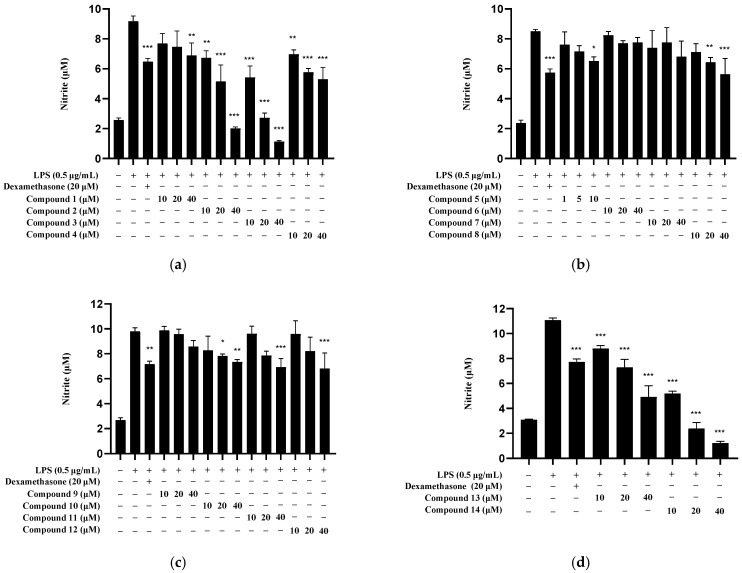
Effect of *Q. acuta* fruit compounds **1**–**14** on nitrite production in LPS-induced BV2 cells. The subfigures represent the following groups: compounds **1**–**4** (**a**), **5**–**8** (**b**), **9**–**12** (**c**), and **13**, **14** (**d**). Cells were treated with compounds **1**–**14** at the indicated concentrations for 3 h and then induced with LPS for 24 h. Dexamethasone was used as the positive control. Data are presented as the mean ± S.D. of three independent experiments (*n* = 3). * *p* < 0.05, ** *p* < 0.01, and *** *p* < 0.001 compared to the LPS-stimulated group.

Based on the cytotoxicity results, the maximum concentration for further testing was set at 10 μM for compound **5** (1, 5, and 10 μM) and at 40 μM for all other compounds (10, 20, and 40 μM). Each compound was used at three different concentrations to evaluate its inhibitory effect on nitrite production in LPS-induced BV2 microglia (Figure 7). Among the active compounds, compounds **2**, **3**, **4**, **13**, and **14** exhibited IC_50_ values within the non-toxic concentration (Table 2). These IC_50_ values were 13.68 ± 2.38 μM for compound **2**, 9.96 ± 3.03 μM for compound **3**, 22.55 ± 0.77 μM for compound **4**, 19.60 ± 1.53 μM for compound **13**, and 7.41 ± 1.40 μM for compound **14**.

### 2.6. Inhibitory Effect of Compounds ***4*** and ***16*** on Inflammatory Cytokine Production

**Figure 8:**
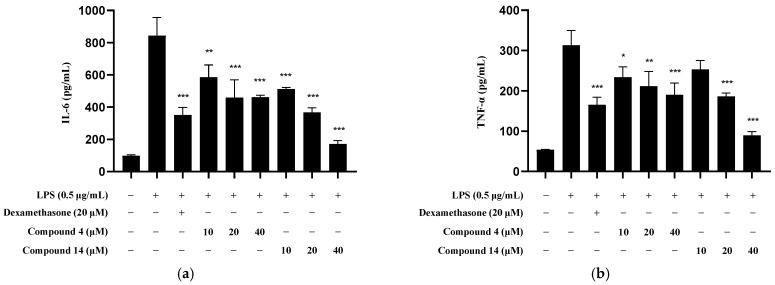
Effect of compound **4** and **14** treatments on the expression of (**a**) IL-6 and (**b**) TNF-α in LPS-induced BV2 cells. Cells were pretreated with the indicated concentrations of either compound **4** or **14** for 3 h and then stimulated with LPS (0.5 μg/mL) for 24 h. Enzyme-linked immunosorbent assays (ELISAs) were performed for protein quantification. Bars represent the mean ± standard deviation of three independent experiments (*n* = 3). * *p* < 0.05, ** *p* < 0.01, and *** *p* < 0.001 vs. the LPS-treated group.

Among the five compounds that showed IC_50_ values under non-toxic conditions, compounds **2**, **3**, and **13** were previously reported to exhibit anti-neuroinflammatory effects [3,4,5,15]. Therefore, compounds **4** (Figure 8a) and **14** (Figure 8b) were selected for further investigation. Our results showed that both compounds inhibited the production of IL-6 and TNF-α in LPS-induced BV2 microglia in a concentration-dependent manner.

### 2.7. Inhibition of iNOS and COX-2 Expression

**Figure 9:**
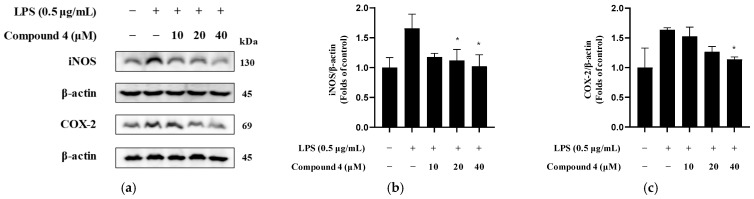

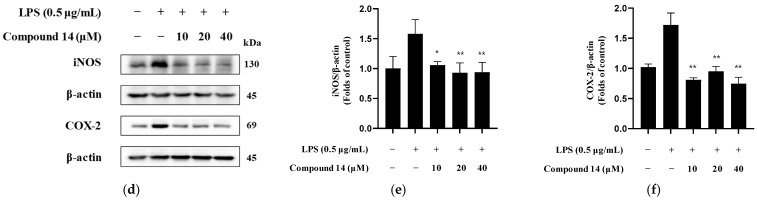
Western blot analysis showing the expression levels of iNOS and COX-2 in BV2 microglia treated with three concentrations (10–40 µM) of compounds (**a**–**c**) **4** and (**d**–**f**) **14** in the presence of 0.5 µg/mL LPS. β-Actin was used as the loading control. Densitometry analyses of COX-2 and iNOS protein levels normalized to β-Actin demonstrated a dose-dependent decrease in protein expression. Data are expressed as the mean ± SEM (*n* ≥ 3). * *p* < 0.05 and ** *p* < 0.01 compared to the LPS-only treated group.

To further investigate the mechanisms underlying the anti-neuroinflammatory effects of compounds **4** and **14**, the expression levels of iNOS and COX-2 proteins were evaluated by Western blot analysis. LPS induction markedly increased the expression of both iNOS and COX-2 in BV2 microglia compared with that in untreated controls. Treatment with compounds **4** (Figure 9a–c) and **14** (Figure 9d–f) significantly reduced the LPS-induced upregulation of these enzymes. These findings indicate that both compounds attenuate the production of NO and prostaglandins by downregulating their key biosynthetic enzymes.

### 2.8. Inhibition of NF-κB Activation

**Figure 10:**
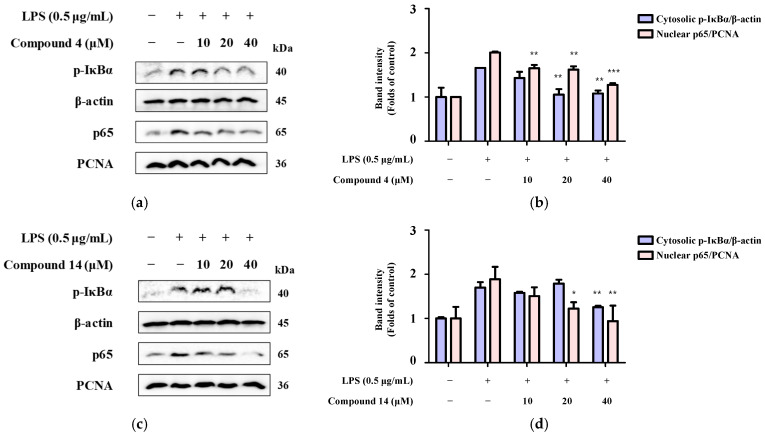
Effects of compounds (**a**,**b**) **4** and (**c**,**d**) **14** on NF-κB activation in BV2 microglia. Cells were pretreated with the indicated concentrations of the compounds for 3 h and then stimulated with LPS for 1 h. Proteins were analyzed by Western blotting and quantified using the ImageJ software (imageJ 1.54g). Band intensities were normalized to those of β-actin. Data represent the average of three independent experiments. Untreated control groups are included (*n* ≥ 3). * p < 0.05, ** *p* < 0.01, and *** *p* < 0.001 compared with the LPS-treated group.

**Figure 11:**
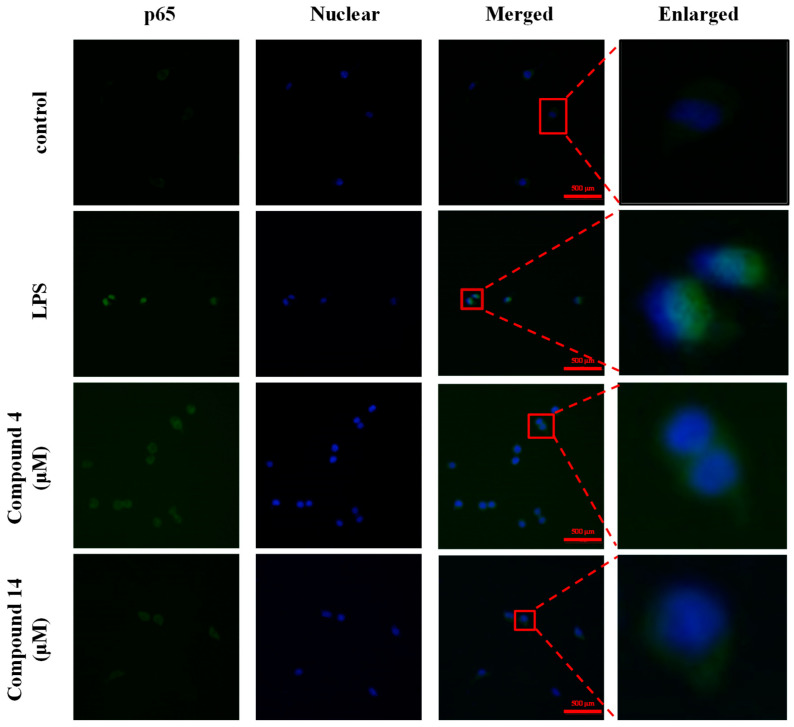
Immunofluorescence analysis of NF-κB signaling activation and p65 nuclear translocation in BV2 microglia treated with compounds **4** or **14**. Immunofluorescence staining was conducted to visualize NF-κB p65 (green) and nuclei (DAPI, blue) in BV2 microglia. Merged images indicate that LPS-induced nuclear translocation of p65 was reduced by compound **4** and **14** treatments. Scale bars are indicated in red.

Given that NF-κB signaling plays a pivotal role in the induction of proinflammatory mediators, we examined whether compounds **4** and **14** influenced NF-κB activation. Our results showed that LPS induction markedly enhanced NF-κB activity, as demonstrated by the nuclear translocation of the p65 subunit, which was confirmed by Western blot analysis of cytoplasmic and nuclear protein fractions, and that cell treatment with compounds **4** (Figure 10a,b) and **14** (Figure 10c,d) inhibited this LPS-induced nuclear translocation of p65 in a concentration-dependent manner. In addition, we detected the nuclear translocation of p65 using immunofluorescence, which further demonstrated the inhibitory effects of compounds **4** and **14** on p65 translocation into the nucleus (Figure 11). These results indicate that both compounds exert anti-neuroinflammatory effects, at least in part, through the inhibition of NF-κB activation.

## 3. Discussion

The genus *Quercus* has long been recognized for its pharmacological potential, particularly with respect to anti-inflammatory, antioxidant, and antimicrobial activities. Previous phytochemical studies of *Q. acuta*, a species native to East Asia, have primarily focused on leaves, stem, and bark, with tannins and galloyl derivatives identified as important contributors to the biological activity of these plant organs. In contrast, the fruits of *Q. acuta* have remained largely unexplored in terms of chemical composition and pharmacological efficacy. To our knowledge, this study provides the first evidence that compounds isolated from *Q. acuta* fruits possess anti-neuroinflammatory activity.

Metabolite profiling using UHPLC-HR-MS/MS revealed that the extract was particularly rich in phenolic compounds. This result suggested that solvent-solvent partitioning, rather than size exclusion, had to be used as a separation method, as phenolic compounds are readily fractionated based on polarity. Similar to the profiling results, most of the isolated compounds belonged to the phenolic family. We then conducted a literature search of previously identified anti-neuroinflammatory effects of the isolated compounds. As expected, some compounds had previously been reported to have anti-neuroinflammatory effects (Table 3).

Subsequently, those isolated compounds that inhibited nitrite production and had not previously been reported to exhibit anti-neuroinflammatory effects were selected for further activity evaluation. Notably, 3,5,7,2′,6′-pentahydroxyflavanone and 2,5-dihydroxybenzaldehyde emerged as the most active constituents. Both compounds significantly suppressed nitrite accumulation in LPS-induced BV2 microglia, indicating their potential as novel anti-neuroinflammatory agents. Further mechanistic analysis revealed that these compounds attenuated the expression of iNOS and COX-2 and reduced the production of inflammatory mediators, including IL-6 and TNF-α. Importantly, both metabolites inhibited NF-κB activation by blocking the nuclear translocation of the p65 subunit, indicating that their anti-neuroinflammatory effects are at least partly mediated through suppression of NF-κB-dependent signaling. While additional upstream regulators (e.g., MAPK pathways or ROS-dependent mechanisms) may also be involved, this remains to be clarified in future studies. Therefore, it is expected to be necessary to employ a specific NF-κB inhibitor and MAPK/ROS-related assays in future studies to clarify the precise upstream mechanisms underlying these anti-neuroinflammatory effects. Although this study was conducted using the BV2 microglial cell model, future validation in primary or human-derived microglia would further strengthen these findings.

In summary, this study demonstrates that *Q. acuta* fruit is a source of 3,5,7,2′,6′-pentahydroxyflavanone and 2,5-dihydroxybenzaldehyde, compounds with previously unknown anti-neuroinflammatory effects. These results demonstrate the potential of *Q. acuta* fruit as a sustainable natural resource for the development of preventative or therapeutic agents for age-related neuro-inflammatory diseases and may contribute to a broader understanding of the pharmacological properties of the genus *Quercus*.

## 4. Materials and Methods

### 4.1. Chemicals, Solvents, and Instruments

Reagent-grade solvents were employed for extractions and column chromatography (CC). LiChroprep^®^ RP-18 resin and silica gel (Merck, Darmstadt, Germany) were used for CC. UHPLC-HR-MS/MS analyses were conducted on a Thermo Vanquish system coupled to a Thermo Q-Exactive mass spectrometer (Thermo Dionex, Sunnyvale, CA, USA). NMR spectra were recorded on a JEOL JNM ECP-400 spectrometer (400 MHz for ^1^H, 100 MHz for ^13^C; JEOL Ltd., Akishima, Japan) using chloroform-d, acetone-d_6_, DMSO-d_6_, and methanol-d_4_ (Cambridge Isotope Laboratories, Andover, MA, USA). HPLC purification was performed on a Waters e2695 system with a semiprep-C18 column (10 × 250 mm, 5 μm) at 2 mL/min.

### 4.2. Plant Materials

Fruits of *Q. acuta* Thunb. were collected from Wando County, Jeollanam-do, Republic of Korea, and preserved at the Natural Resources Laboratory of the Jeonnam Bio Foundation (JBF), Republic of Korea (voucher specimen: JBF1201). The plant material was taxonomically identified by Dr. Yonguk Kim, an expert in plant identification at the JBF.

### 4.3. Cell Culture and Reagents

BV2 microglia were routinely cultured in RPMI-1640 medium supplemented with 10% heat-inactivated fetal bovine serum (FBS) and an antibiotic–antimycotic solution (Thermo Fisher Scientific, Waltham, MA, USA) under standard conditions (37 °C, 5% CO_2_, humidified incubator). Culture plates were obtained from Corning (Amsterdam, The Netherlands), and unless otherwise stated, all other reagents were purchased from Sigma-Aldrich (St. Louis, MO, USA).

Cytotoxicity was assessed by the MTT assay. BV2 cells were seeded in 48-well plates at a density of 5 × 10^4^ cells per well and allowed to adhere overnight. The next day, cells were treated with different concentrations of the test compounds for 24 h. Subsequently, MTT solution (5 mg/mL) was added, and cells were further incubated for 4 h to allow the formation of formazan crystals. After carefully discarding the supernatant, the crystals were solubilized with dimethyl sulfoxide (DMSO), and absorbance was determined at 540 nm using a microplate reader (Molecular Devices, San Jose, CA, USA). Dexamethasone served as positive control.

### 4.4. Metabolite Profiling Analysis

Metabolite profiling was performed using a Thermo Vanquish UHPLC system (Thermo Fisher Scientific, Sunnyvale, CA, USA) equipped with a CORTECS T3 column (2.1 × 150 mm, 1.6 μm; Waters Technologies, Milford, MA, USA); it was maintained at 45 °C. The mobile phases consisted of solvent A (0.1% formic acid in water, *v*/*v*) and solvent B (0.1% formic acid in acetonitrile, *v*/*v*). The gradient program was as follows: 0–0.5 min, 3% B; 0.5–15 min, 15% B; 15–40 min, 75% B; 40–41 min, 100% B; 41–45 min, 100% B; 45–45.1 min, 3% B; and 45.1–50 min, 3% B. The flow rate was 0.25 mL/min, the injection volume was 3 μL, and the sample was prepared by dissolving *Q. acuta* extract in ethanol at 20 mg/mL.

High-resolution mass spectrometry (HRMS) was performed on a Thermo Q Exactive mass spectrometer (Thermo Fisher Scientific, Bremen, Germany) equipped with a heated electrospray ionization (H-ESI) source, operated in both positive and negative ion modes. The MS source parameters were as follows: spray voltage, 3500 V (positive mode) and 3000 V (negative mode); sheath gas, 50 (arbitrary units); auxiliary gas, 10 (arbitrary units); sweep gas, 1 (arbitrary unit); and ion transfer tube temperature, 320 °C. Full MS scans were acquired at a resolution of 35,000 (*m*/*z* 200) over a mass range of *m*/*z* 100–1500. Data-dependent MS/MS spectra were acquired at a resolution of 17,500 with a TopN of 10 and stepped normalized collision energies (NCE) of 10, 30, and 50 eV. Data acquisition and processing were performed using Elements Viewer version 2.1.

### 4.5. Compound Extraction and Isolation

Dried fruits of *Q. acuta* (9 kg) were extracted with 70% EtOH (9 L) at 100 °C for 3 h. The extract was filtered and evaporated under reduced pressure to yield a crude extract (600 g) that was dissolved in distilled water (1.5 L). The solution was then partitioned successively with *n*-hexane (1.5 L × 2), chloroform (1.5 L × 2), ethyl acetate (1.5 L × 2), and *n*-butanol (1.5 L × 2). Each organic layer was evaporated under reduced pressure to obtain *n*-hexane (QA-Hx, 11.5 g), chloroform (QA-CF, 7.2 g), ethyl acetate (QA-EA, 24.4 g), *n*-butanol (QA-Bu, 28.6 g), and an aqueous fraction (QA-H_2_O, 218.7 g).

First, 5 g of the QA-CF fraction was fractionated by C18 CC (MeOH:H_2_O, 1:1–1:0) using a bioassay guidance to obtain five subfractions (QA-CF-1–5). Among these, QA-CF-1 was purified to yield compound **1** (QA-CF-1, 51.7 mg, purity 97.84%). Next, the QA-CF-2 fraction, which showed purification potential using UV at 254 nm as determined by TLC analysis, was fractionated using C18 CC under isocratic system (MeOH:H_2_O, 1:2), yielding three subfractions (QA-CF-2-1–3). Among them, QA-CF-2-1 was purified to obtain compound **2** (QA-CF-2-1, 21.5 mg, purity 96.29%), whereas QA-CF-2-2 was purified to obtain compound **3** (QA-CF-2-2-1, 12.2 mg, purity 96.81%).

The QA-EA fraction (10 g) was then fractionated by C18 CC (MeOH:H_2_O, 1:4–1:0) to obtain five subfractions (QA-EA-1–5). Among these subfractions, QA-EA-2 and QA-EA-3 were confirmed to have anti-inflammatory properties through bioassay guidance. Therefore, QA-EA-2 was further separated into five subfractions (QA-EA-2-1–5) using MPLC with a C18 column (MeOH:H_2_O, 0.05:0.95–1:0). QA-EA-2-2 was further divided into five subfractions (QA-EA-2-2-1–5), among which QA-EA-2-2-3 was purified by preparative HPLC (MeOH:H_2_O, 1:4–1:0) to yield compound **4** (QA-EA-2-2-3-3, 6.9 mg, purity 95.57%). Subsequently, QA-EA-2-5 was purified by C18 CC (MeOH:H_2_O, 1:9–1:1) to yield compound **5** (QA-EA-2-5-1, 80.0 mg, purity 95.04%). QA-EA-3 was fractionated by SiO_2_ CC (CHCl_3_:80% MeOH, 3:1–1:1) to obtain eight subfractions (QA-EA-3-1–8). Among these subfractions, QA-EA-3 was purified by C18 preparative HPLC to yield compound **6** (QA-EA-3-1-1, 6.1 mg, purity 97.97%) and compound **7** (QA-EA-3-1-2, 7.2 mg, purity 99.54%). Fractions QA-EA-3-6 and QA-EA-3-7, which were readily detectable under UV at 254 nm in TLC analysis, were purified by C18 preparative LC to yield compound **8** (QA-EA-3-6-3, 11.0 mg, purity 98.31%) and compound **9** (QA-EA-3-7-2, 51.5 mg, purity 95.48%). In addition, QA-EA-8 was purified by SiO_2_ CC (EtOAc:50% MeOH, 3:1–1:1) to yield compound **10** (QA-EA-3-8-1, 7.1 mg, purity 96.79%) and QA-EA-5 was purified by C18 preparative LC to yield compound **11** (QA-EA-5-1, 4.7 mg, purity 98.79%).

The QA-Bu fraction (20 g) was then purified by C18 CC (MeOH:H_2_O, 1:4–1:0), yielding five subfractions (QA-Bu-1–5). QA-Bu-1 was further purified by MPLC with a C18 column (MeOH:H_2_O, 0.1:0.9–1:0) to obtain six subfractions (QA-Bu-1-1–6). Among these subfractions, QA-Bu-1-6 was purified by C18 preparative HPLC to yield compound **12** (QA-Bu-1-6-1, 6.1 mg, purity 97.92%), while QA-Bu-2 was purified by MPLC (C18 column) to obtain three subfractions (QA-Bu-2-1–3), and QA-Bu-2-3 was further purified by C18 preparative LC to yield compound **13** (QA-Bu-2-3-2, 10.1 mg, purity 96.83%). Next, QA-Bu-4 was purified by SiO_2_ CC (EtOAc:50% MeOH, 4:1–1:1) under gradient systems to yield seven subfractions (QA-Bu-4-1–7). Among them, QA-Bu-4-5 was further separated by SiO_2_ CC (EtOAc:50% MeOH, 4:12:1) to yield four subfractions (QA-Bu-4-5-1–4), and QA-Bu-4-5-2 was purified by C18 preparative HPLC to yield compound **14** (QA-Bu-4-5-2-2, 5.8 mg, purity 96.07%). Overall, all purified compounds reached a final purity of ≥95%, as confirmed by HPLC (Figures S44 and S45).

Isolated compounds were further identified through NMR as follows. NMR spectra were recorded in CDCl_3_, CD_3_OD, acetone-d_6_, or DMSO-d_6_ depending on the solubility and spectral quality of each compound.

Gallic acid (compound **1**): ^1^H NMR (400 MHz, methanol-d_4_) δ 7.05 (2H, s, H-2, H-6). ^13^C NMR (100 MHz, methanol-d_4_) δ 170.7 (C-7), 146.5 (C-3, C-5), 139.4 (C-4), 122.9 (C-1), 110.4 (C-2, C-6) [50] (Figures S2–S5).

Sinapaldehyde (compound **2**): ^1^H NMR (400 MHz, acetone-d_6_) δ 9.62 (1H, d, *J* = 7.6 Hz, H-9), 7.94 (1H, s, Ar–OH), 7.55 (1H, d, *J* = 15.6 Hz, H-7), 7.06 (2H, s, H-2, H-6), 6.69 (1H, dd, *J* = 15.6, 7.6 Hz, H-8), 3.88 (6H, s, 2xOCH_3_). ^13^C NMR (100 MHz, acetone-d_6_) δ 193.0 (C-9), 153.6 (C-7), 148.2 (C-3, C-5), 139.4 (C-4), 126.5 (C-8), 125.3 (C-1), 106.5 (C-2, C-6), 55.9 (10-OCH_3_, 11-OCH_3_) [51] (Figures S6 and S7).

Coniferaldehyde (compound **3**): ^1^H NMR (400 MHz, chloroform-d) δ 9.65 (1H, d, *J* = 7.6 Hz, H-9), 7.42 (1H, d, *J* = 16.0 Hz, H-7), 7.13 (1H, m, H-6), 6.97 (1H, d, *J* = 8.4 Hz, H-5), 6.62 (1H, dd, *J* = 15.6, 7.6 Hz, H-8), 3.94 (3H, s, OCH_3_). ^13^C NMR (100 MHz, chloroform-d) δ: 193.8 (C-9), 153.3 (C-7), 149.0 (C-3), 147.0 (C-5), 126.5 (C-1), 124.2 (C-6), 115.0 (C-5), 109.5 (C-2), 56.1 (OCH_3_) [50] (Figures S8 and S9).

3,5,7,2′,6′-Pentahydroxyflavanone (compound **4**): ^1^H NMR (400 MHz, acetone-d_6_) δ 11.83 (1H, s, 5-OH), 9.63 (1H, s, 3-OH), 8.57 (1H, s, 2′-OH, 6′-OH), 7.02 (1H, t, *J* = 8.4 Hz, H-4′), 6.45 (2H, d, *J* = 8.0 Hz, H-3′, H-5′), 5.95 (1H, d, *J* = 2.0 Hz, H-8), 5.90 (1H, d, *J* = 2.4 Hz, H-6), 5.81 (1H, d, *J* = 12.0 Hz, H-2), 5.51 (1H, dd, *J* = 12.4, 4.0 Hz, H-3). ^13^C NMR (100 MHz, acetone-d_6_) δ 198.9 (C-4), 166.6 (C-7), 164.4 (C-5), 164.3 (C-9), 158.1 (C-2′, C-6′), 130.3 (C-4′), 109.8 (C-1′), 107.4 (C-3′, C-5′), 100.8 (C-10), 95.9 (C-6), 95.0 (C-8), 76.0 (C-2), 69.5 (C-3) [52] (Figures S10 and S11).

1,2,3,6-Tetrakis-*O*-galloyl-β-*D*-glucose (compound **5**): ^1^H NMR (400 MHz, methanol-d_4_) δ 7.11 (2H, s, H-2, 6 of galloyl-O-6), 7.02 (2H, s, H-2, 6 of galloyl-O-2&3), 7.01 (2H, s, 2H, s, H-2, 6 of galloyl-O-2&3), 6.92 (2H, s, 2H, s, H-2, 6 of galloyl-O-1), 6.09 (1H, d, *J* = 7.6 Hz, H-1), 5.59–5.55 (1H, m, H-5), 5.45–5.41 (1H, m, H-2), 4.61 (1H, dd, *J* = 12.4, 2.0 Hz, H-6α), 4.53 (1H, dd, *J* = 12.0, 4.0 Hz, H-6β), 4.02 (1H, dd, *J* = 7.6, 1.6 Hz, H-3), 3.97–3.93 (1H, m, H-4), 2.60 (3H, s). ^13^C NMR (100 MHz, methanol-d_4_) 166.9 (C-7 of galloyl-O-6), 166.4 (C-7 of galloyl-O-3), 165.9 (C-7 of galloyl-O-2), 165.0 (C-7 of galloyl-O-1), 145.2 (C-3, 5 of galloyl-O-1&2), 145.0 (C-3, 5 of galloyl-O-1&2), 139.4 (C-4 of galloyl-O-1), 138.9 (C-4 of galloyl-O-2), 138.6 (C-4 of galloyl-O-3&6), 119.9 (C-1 of galloyl-O-6), 119.6 (C-1 of galloyl-O-1), 119.1 (C-1 of galloyl-O-2), 118.5 (C-1 of galloyl-O-3), 109.2 (C-2, 6 of galloyl-O-1), 109.1 (C-2, 6 of galloyl-O-2&3), 109.0 (C-2, 6 of galloyl-O-2&3), 108.7 (C-2, 6 of galloyl O-6), 92.6 (C-1), 75.3 (C-5), 75.1 (C-3), 71.0 (C-2), 68.3 (C-4), 62.7 (C-6) [53] (Figures S12–S17).

Phlorizin (compound **6**): ^1^H NMR (400 MHz, methanol-d_4_) δ 7.06 (2H, d, *J* = 8.8 Hz, H-3′, H-5′), 6.67 (2H, d, *J* = 8.8 Hz, H-2′, H-6′), 6.16 (1H, d, *J* = 2.4 Hz, H-3), 5.94 (1H, d, *J* = 2.4 Hz, H-5), 5.03 (1H, d, *J* = 7.2 Hz, H-1″), 3.91 (1H, dd, *J* = 12.0, 2.0 Hz, H-6″α), 3.72 (1H, dd, *J* = 12.0, 5.6 Hz, H-6″β), 3.45 (2H, m, H-8), 3.17 (1H, m, H-3″), 3.15 (1H, m, H-2″), 3.12 (1H, m, H-5″), 2.86 (2H, t, *J* = 7.6 Hz, H-9). ^13^C NMR (100 MHz, methanol-d_4_) δ 205.2 (C-7), 166.3 (C-2), 164.7 (C-6), 161.0 (C-4), 155.0 (C-4′), 132.5 (C-1′), 129.1 (C-3′, C-5′), 114.7 (C-2′, C-6′), 105.4 (C-1), 100.7 (C-1″), 97.0 (C-3), 94.1 (C-5), 77.1 (C-3″), 77.1 (C-5″), 73.4 (C-2″), 69.7 (C-4″), 61.1 (C-6″), 45.7 (C-8), 29.5 (C-9) [54] (Figures S18 and S19).

3,4-Dimethoxycinnamic acid (compound **7**): ^1^H NMR (400 MHz, DMSO-d_6_) δ 12.2 (1H, s, H-1), 7.51 (1H, d, *J* = 16.0 Hz, H-8), 7.28 (1H, d, *J* = 2.0 Hz, H-2′), 7.18 (1H, dd, *J* = 8.8, 2.0 Hz, H-6′), 6.95 (1H, d, *J* = 8.4 Hz, H-5′), 6.43 (1H, d, *J* = 16.0 Hz, H-7), 3.77 (3H, s, 3′-OCH_3_), 3.75 (3H, s, 4′-OCH_3_). ^13^C NMR (100 MHz, DMSO-d_6_) δ 168.5 (C-1), 151.3 (C-3′), 149.5 (C-4′), 144.7 (C-8), 127.6 (C-1′), 123.2 (C-6′), 117.2 (C-5′), 112.0 (C-7), 110.7 (C-2′), 56.1 (3′-OCH_3_), 56.1 (4′-OCH_3_) [55] (Figures S20 and S21).

Ferulic acid (compound **8**): ^1^H NMR (400 MHz, chloroform-d) δ 7.73 (1H, d, *J* = 15.6 Hz, H-1′), 7.11 (1H, dd, *J* = 8.4, 2.0 Hz, H-5), 7.05 (1H, d, *J* = 2.0 Hz, H-3), 6.94 (1H, d, *J* = 8.0 Hz, H-6), 6.31 (1H, d, *J* = 16.0 Hz, H-2′), 3.98 (3H, s, OCH_3_). ^13^C NMR (100 MHz, chloroform-*d*) δ 171.5 (C-3′), 148.5 (C-1), 147.3 (C-6), 146.9 (C-1′), 126.7 (C-4), 123.7 (C-3), 114.9 (C-2′), 114.3 (C-2), 109.6 (C-5), 56.1 (OCH_3_) [56] (Figures S22 and S23).

Taxifolin (compound **9**): ^1^H NMR (400 MHz, methanol-d_4_) δ 6.95 (1H, d, *J* = 2.0 Hz, H-2′), 6.84 (1H, dd, *J* = 8.4, 2.0 Hz, H-6′), 6.79 (1H, d, *J* = 8.0 Hz, H-5′), 5.90 (1H, d, *J* = 2.0 Hz, H-8), 5.87 (1H, d, *J* = 2.4 Hz, H-6), 4.91 (1H, d, *J* = 11.6 Hz, H-2), 4.50 (1H, d, *J* = 11.6 Hz, H-3). ^13^C NMR (100 MHz, methanol-d_4_) δ 197.1 (C-4), 167.4 (C-7), 164.0 (C-5), 163.2 (C-8a), 145.8 (C-4′), 145.0 (C-3′), 128.5 (C-1′), 119.6 (C-6′), 114.7 (C-2′), 114.5 (C-5′), 100.5 (C-4a), 96.0 (C-6), 94.9 (C-8), 83.8 (C-2), 72.3 (C-3) [57] (Figures S24 and S25).

Catechin (compound **10**): ^1^H NMR (400 MHz, DMSO-d_6_) δ 9.13 (1H, s, 5-OH), 8.89 (1H, s, 7-OH), 8.82 (1H, s, 4′-OH), 8.82 (1H, s, 3′-OH), 6.69 (1H, d, *J* = 2.0 Hz, H-2′), 6.66 (1H, d, *J* = 8.0 Hz, H-5′), 6.57 (1H, dd, *J* = 8.4, 2.0 Hz, H-6′), 5.85 (1H, d, *J* = 2.0 Hz, H-8), 5.65 (1H, d, *J* = 2.4 Hz, H-6), 4.45 (1H, d, *J* = 7.6 Hz, H-2), 3.79 (1H, m, H-3), 2.65 (1H, dd, *J* = 3.6, 2.0 Hz, H-4α), 2.61 (1H, d, *J* = 8.0 Hz, H-4β). ^13^C NMR (100 MHz, DMSO-d_6_) δ 157.0 (C-5), 156.7 (C-7), 155.9 (C-8a), 145.4 (C-3′, C-4′), 131.1 (C-1′), 118.9 (C-6′), 115.6 (C-5′), 115.0 (C-2′), 99.6 (C-4a), 95.6 (C-6), 94.3 (C-8), 81.5 (C-2), 66.8 (C-3), 28.4 (C-4) [58] (Figures S26 and S27).

Ellagic acid (compound **11**): ^1^H NMR (400 MHz, DMSO-d_6_) δ 10.67 (1H, br, s, OH), 7.43 (2H, s, H-2, H-2′). ^13^C NMR (100 MHz, DMSO-d_6_) δ 159.7 (C-7, C-7′), 148.7 (C-4, C-4′), 140.1 (C-2, C-2′), 136.9 (C-3, C-3′), 112.9 (C-6, C-6′), 110.8 (C-5, C-5′), 108.2 (C-1, C-1′) [59] (Figures S28–S31).

Protocatechuic acid (compound **12**): ^1^H NMR (400 MHz, methanol-d_4_) δ 7.42 (1H, s, H-5), 7.40 (1H, d, *J* = 2.0 Hz, H-2), 6.79 (1H, d, *J* = 8.4 Hz, H-6). ^13^C NMR (100 MHz, methanol-d_4_) δ 168.9 (C-7), 150.2 (C-4), 144.7 (C-3), 122.5 (C-1), 121.8 (C-5), 116.4 (C-6), 114.4 (C-2) [60] (Figures S32–S36).

Corilagin (compound **13**): ^1^H NMR (400 MHz, acetone-d_6_) δ 7.10 (2H, s, Galloyl H-2, H-6), 6.82 (1H, s, HHDPa-H), 6.67 (1H, s, HHDPb-H), 6.35 (1H, s, H-1), 4.97 (1H, t, H-6a), 4.82 (1H, m, H-3), 4.52 (1H, m, H-5), 4.47 (1H, m, H-4), 4.10 (2H, m, H-2, H-6b). ^13^C NMR (100 MHz, acetone-d_6_) δ 167.8 (galloyl-C-7), 166.4 (HHDPa-C-7), 164.2 (HHDPb-C-7), 145.1 (Galloyl-C-3, C-4), 144.6 (HHPDb-C-3, C-4), 144.1 (HHPDa-C-3), 144.0 (HHPDa-C-4), 138.4 (Galloyl-C-1), 136.4 (HHPDa-C-1), 135.9 (HHPDb-C-1), 125.0 (HHPDa-C-2, HHPDb-C-2), 124.8 (HHPDa-C-5, HHPDb-C-5), 120.0 (HHDPa-C-6, HHDPb-C-6), 115.7 (HHDP-bridge C-1), 115.0 (HHDP-bridge C-2), 110.0 (Galloyl-C-2, C-6), 109.1 (HHDPa-CH), 107.3 (HHDPb-CH), 93.3 (C-1), 74.9 (C-5), 69.9 (C-3), 68.2 (C-2), 63.5 (C-6), 61.5 (C-4) [61] (Figures S37–S41).

2,5-Dihydroxybenzaldehyde (compound **14**): ^1^H NMR (400 MHz, acetone-d_6_) δ 10.38 (1H, s, OH), 9.92 (1H, s, H-1), 8.36 (1H, s, 5-OH), 7.15 (1H, d, *J* = 0.4 Hz, H-6), 7.14 (1H, dd, *J* = 8.8, 3.2 Hz, H-4), 7.10 (1H, d, *J* = 3.2 Hz, H-3). ^13^C NMR (100 MHz, acetone-d_6_) δ 196.7 (C-1), 154.8 (C-2), 150.4 (C-5), 125.2 (C-1′), 121.0 (C-6), 118.0 (C-4), 117.5 (C-3) [62] (Figures S42 and S43).

### 4.6. Measurement of Nitrite Production

To induce inflammation in BV2 cells, we treated the cells with LPS (0.5 µg/mL) for 24 h, following conditions commonly used in previous studies, and then performed the subsequent experiments [63,64,65]. NO, a representative proinflammatory mediator, was quantified in LPS-induced BV2 microglia using the Griess reaction. This assay is based on the principle that nitrite (NO_2_^−^), a stable metabolite of NO, reacts with sulfanilamide to generate a diazonium intermediate, which subsequently couples with N-(1-naphthyl) ethylenediamine to form a red azo dye [63,66]. The intensity of the color produced reflects the concentration of NO present in the sample. After BV2 cells were treated with the test extract, its fractions, or the isolated compounds under inflammatory conditions, culture supernatants were collected and mixed with an equal volume of Griess reagent (1:1). The absorbance of the resulting solution was measured at 570 nm using a microplate reader. Dexamethasone (20 μM), a well-known inhibitor of inflammatory responses, was employed as the positive control. The 50% inhibitory concentration (IC_50_) values were calculated using GraphPad Prism 9.5.0 software (GraphPad Software Inc., San Diego, CA, USA).

### 4.7. IL-6 and TNF-α Analysis

The secretion of the proinflammatory cytokines IL-6 and TNF-α was quantified in BV2 culture supernatants using ELISA kits obtained from BioLegend (San Diego, CA, USA), according to the supplier’s protocols. After exposure of BV2 cells to the test compounds under inflammatory conditions, culture media were harvested and assayed at 450 nm. The cytokine-suppressive effect of dexamethasone was used as the positive control.

### 4.8. Western Blot Analysis

The expression of inflammation-related proteins, including iNOS, COX-2, nuclear p65, and phosphorylated IκBα, was evaluated by Western blotting. LPS-induced BV2 cells were harvested and lysed in 20 mM Tris-HCl buffer (pH 7.4) supplemented with a protease inhibitor cocktail (0.1 mM phenylmethanesulfonyl fluoride, 5 μg/mL aprotinin, 5 μg/mL pepstatin A, and 1 μg/mL chymostatin). Protein concentrations were quantified using a protein assay dye reagent concentrate (#5000006; Bio-Rad Laboratories, Hercules, CA, USA). Equal amounts of protein (30 μg) were separated on 7.5–12% SDS-polyacrylamide gels and transferred to nitrocellulose membranes (Hybond ECL; Bio-Rad Laboratories). After blocking with 5% skim milk, membranes were sequentially incubated with specific primary antibodies followed by horseradish peroxidase (HRP)-conjugated secondary antibodies. Target proteins were visualized using enhanced chemiluminescence reagents (Pierce Biotechnology, Rockford, IL, USA) according to the manufacturer’s instructions.

### 4.9. NF-κB Localization and Immunofluorescence

To examine the intracellular localization of NF-κB, BV2 cells were seeded onto Lab-Tek II chamber slides (Thermo Fisher Scientific, Waltham, MA, USA). After 24 h of attachment, cells were pretreated with the test compound for 3 h and subsequently induced with LPS (0.5 μg/mL) for 30 min. The cells were then fixed with 4% paraformaldehyde, permeabilized with 0.1% Triton X-100, and blocked with 5% bovine serum albumin. Slides were incubated with a primary antibody against NF-κB p65, followed by a fluorophore-conjugated secondary antibody. Nuclei were counterstained with 4′,6-diamidino-2-phenylindole (DAPI). The stained cells were observed using a fluorescence microscope (Zeiss, Oberkochen, Germany), and images were captured for analysis of NF-κB nuclear translocation.

### 4.10. Statistical Analysis

All experimental results are expressed as the mean ± standard deviation (S.D.) of at least three independent replicates. Statistical significance was determined using one-way analysis of variance (ANOVA) followed by Tukey’s multiple comparison test. Data processing and graph generation were carried out with GraphPad Prism software, version 9.5.0.

## 5. Conclusions

In this study, 14 compounds from an ethanol extract of *Quercus acuta* fruits—which were chemically underexplored—were successfully isolated and structurally characterized. Among them, compound **4** (identified as 3,5,7,2′,6′-pentahydroxyflavanone) and compound **14** (identified as 2,5-dihydroxybenzaldehyde) were identified for the first time as inhibitors of neuro-inflammatory responses in BV2 microglia. Both compounds significantly reduced the production of NO and proinflammatory cytokines, downregulated iNOS and COX-2 expression, and suppressed NF-κB activation, indicating that their anti-neuroinflammatory effects are mediated, at least in part, through NF-κB-dependent pathways.

Collectively, our findings provide the first comprehensive account of bioactive constituents from the *Q. acuta* fruit and demonstrate the potential of this fruit as a promising natural resource for the development of novel therapeutic agents against neuro-inflammation and neurodegenerative diseases.

## Figures and Tables

**Table 1:** Tentative identification of the chemical compounds obtained from the UPLC-HR-MS/MS analysis of a *Q. acuta* fruit EtOH extract.

R.T. (min)	M.F.	M.W.	Adduct	MS2 Ion Fragments (*m*/*z*)	I.D.	M.A.S.	Class	I.D. ref.
2.01	C_13_H_16_O_10_	332.1	[M − H]^−^	125.023, 169.011	Gallic acid hexoside	1	Phenol	[20]
2.46	C_7_H_6_O_5_	170.0	[M + H]^+^	81.034, 107.013, 127.039, 153.018	Gallic acid	1	Phenol	[20,21]
3.70	C_20_H_20_O_13_	126.0	[M + H]^+^	53.039, 81.034, 109.029	Pyrogallol	0.97	Phenol	[23]
4.00	C_13_H_16_O_10_	468.1	[M + H]^+^	111.044, 125.024, 153.018, 299.076	Ginnalin A	1	Phenol	[24]
4.56	C_20_H_20_O_14_	484.1	[M − H]^−^	85.028, 95.012, 113.023, 125.023, 169.013, 313.057	Hamamelitannin	1	Tannin	[25]
4.74	C_7_H_6_O_4_	154.0	[M − H]^−^	81.033, 108.020, 109.028	Protocatechuic acid	0.81	Phenol	[19,21]
6.96	C_7_H_6_O_3_	138.0	[M − H]^−^	81.033, 108.020, 109.028, 136.015	Protocatechuic aldehyde	0.77	Phenol	[26]
9.20	C_30_H_26_O_12_	578.1	[M − H]^−^	109.028, 125.023, 161.024, 289.072, 339.086, 407.077, 425.089	Procyanidin B1	1	Flavonoid	[20,25]
9.68	C_7_H_6_O_3_	138.0	[M + H]^+^	55.019, 65.039, 69.034, 111.044	2,5-Dihydroxybenzaldehyde	1	Phenol	[32]
9.68	C_15_H_14_O_6_	290.1	[M − H]^−^	57.033, 97.028, 109.028, 125.023, 151.039, 203.070, 245.082	Catechin	1	Flavonoid	[22,23]
11.12	C_27_H_22_O_18_	634.1	[M − H]^−^	123.008, 125.023, 169.013, 173.024, 185.024, 275.021, 300.999	Corilagin	0.98	Tannin	[20]
12.19	C_27_H_24_O_18_	636.1	[M − H]^−^	65.853, 75.418, 85.028, 95.012, 125.023, 169.013, 313.057, 465.067	1,3,6-tri-O-galloyl glucose	0.98	Tannin	[22,25]
13.28	C_15_H_14_O_6_	290.1	[M − H]^−^	109.028, 123.044, 151.039, 179.034, 203.071, 245.082, 289.072	Epicatechin	0.97	Flavonoid	[20,25]
13.37	C_16_H_18_O_8_	338.1	[M + H]^+^	-	1-Coumaroylquinic acid	1	Phenol	[19]
13.76	C_10_H_8_O_5_	208.0	[M + H]^+^	107.049, 135.044, 153.055, 163.039, 194.021	Fraxetin	0.99	Coumarin	[27]
14.53	C_15_H_12_O_7_	304.1	[M + H]^+^	123.044, 149.023, 153.018, 195.029, 231.065, 259.060, 287.055	(2R,3R)-2-(2,6-dihydroxyphenyl)-3,5,7-trihydroxy-2,3-dihydrochromen-4-one	0.99	Flavonoid	[23]
15.51	C_21_H_22_O_12_	466.1	[M − H]^−^	107.012, 151.003, 151.039, 178.997, 285.041, 303.003, 339.073	(2R,3R)-2-(3,4-dihydroxyphenyl)-5,7-dihydroxy-3-[(2S,3R,4S,5S,6R)-3,4,5-trihydroxy-6-(hydroxymethyl)oxan-2-yl]oxy-2,3-dihydrochromen-4-one	1	Flavonoid	[33]
16.65	C_10_H_8_O_4_	192.0	[M + H]^+^	133.029, 149.060, 150.031, 178.026	Scopoletin	0.98	Coumarin	[26]
16.90	C_10_H_10_O_4_	194.1	[M − H]^−^	-	Ferulic acid	0.91	Phenol	[20]
17.91	C_15_H_12_O_7_	304.1	[M + H]^+^	123.044, 149.023, 153.018, 231.065, 259.060	Taxifolin	1	Flavonoid	[21]
18.12	C_11_H_10_O_5_	222.1	[M + H]^+^	107.050, 134.036, 162.031, 190.026, 208.037	Fraxidin	1	Coumarin	[31]
18.78	C_14_H_6_O_8_	302.0	[M + H]^+^	173.024, 201.018, 229.013, 257.008, 285.003	Ellagic acid	1	Tannin	[20,21]
18.86	C_25_H_32_O_13_	540.2	[M − H]^−^	59.012, 89.025, 101.025, 123.008, 163.111	Oleuropein	1	Phenol	[30]
19.23	C_22_H_18_O_10_	442.1	[M − H]^−^	-	Epicatechin gallate	1	Flavonoid	[22]
19.38	C_15_H_12_O_8_	320.1	[M − H]^−^	102.288, 133.029, 145.028, 173.024, 189.019	Dihydromyricetin	1	Flavonoid	[29]
19.41	C_10_H_10_O_3_	178.1	[M + H]^+^	55.019, 119.049, 133.065, 147.044, 161.060	Coniferaldehyde	1	Phenol	[26]
20.18	C_11_H_12_O_4_	208.1	[M + H]^+^	-	Sinapoyl aldehyde	1	Phenol	[26]
21.17	C_21_H_24_O_10_	436.1	[M + H]^+^	81.034, 109.029, 125.022, 143.034, 171.029	Phloretin-2′-O-glucoside	1	Phenol	[19]
22.55	C_11_H_12_O_4_	208.1	[M + H]^+^	133.065, 148.052, 163.075, 191.071	Dimethylcaffeic acid	1	Phenol	[28]
22.76	C_19_H_22_O_5_	330.1	[M + H]^+^	137.060, 151.075, 189.091, 227.107, 255.102, 285.112, 287.128, 313.144, 331.202	Tetrahydrosappanone A Trimethyl Ether	0.99	Phenol	[34]
23.82	C_15_H_10_O_7_	302.0	[M − H]^−^	-	Quercetin	0.99	Flavonoid	[19,20]

I.D., identification; I.D. ref., reference used for identification; M.A.S., MS Accuracy score; M.F., Molecular formula; M.W., Molecular weight; R.T., Retention time.

**Table 2:** IC_50_ (µM ^a^) values of isolated compounds that suppress nitrite production in LPS-induced BV2 microglia.

Compound	Name	IC_50_ ± S.D.
Compound **1**	Gallic acid	-
Compound **2**	Sinapaldehyde	13.68 ± 2.38
Compound **3**	Coniferaldehyde	9.96 ± 3.03
Compound **4**	3,5,7,2′,6′-pentahydroxyflavanone	22.55 ± 0.77
Compound **5**	1,2,3,6-Tetrakis-O-galloyl-beta-D-glucose	-
Compound **6**	Phlorizin	-
Compound **7**	3,4-Dimethoxycinnamic acid	-
Compound **8**	Ferulic acid	-
Compound **9**	Taxifolin	-
Compound **10**	Catechin	-
Compound **11**	Ellagic acid	-
Compound **12**	Protocatechuic acid	-
Compound **13**	Corilagin	19.60 ± 1.53
Compound **14**	2,5-Dihydroxybenzaldehyde	7.41 ± 1.40

S.D., standard deviation. IC_50_, the IC_50_ value was defined as the concentration of the isolated compounds that reduced the LPS-induced response by 50% relative to the difference between the control and LPS-treated group. ^a^ Data represent the mean ± SD of three independent experiments.

**Table 3:** Studies on the anti-neuroinflammatory effects of the compounds isolated from *Q. acuta* fruits.

Compound Name (I.D. Number)	Neuro-Inflammation Activity Determination Model	Ref.
Gallic acid (**1**)	BV2 microglial cells, rat primary microglia	[35,36]
Sinapaldehyde (**2**)	BV2 microglial cells	[37]
Coniferaldehyde (**3**)	BV2 microglial cells, leptomeningeal cells	[38,39]
3,5,7,2′,6′-Pentahydroxyflavanone (**4**)	-	-
1,2,3,6-Tetrakis-O-galloyl-β-D-glucose (**5**)	-	-
Phlorizin (**6**)	Mice	[40]
3,4-Dimethoxycinnamic acid (**7**)	BV2 microglial cells	[41]
Ferulic acid (**8**)	BV2 microglial cells, rats, mice	[42,43,44]
Taxifolin (**9**)	BV2 microglial cells	[45]
Catechin (**10**)	BV2 microglial cells	[46]
Ellagic acid (**11**)	Rats	[47]
Protocatechuic acid (**12**)	Mice	[48]
Corilagin (**13**)	BV2 microglial cells	[49]
2,5-Dihydroxybenzaldehyde (**14**)	-	-

I.D., identification; Ref., reference.

## Data Availability

The data presented in this study are available upon request from the corresponding author.

## Supplementary Materials

The following supporting information can be downloaded at: https://www.mdpi.com/article/10.3390/molecules30234514/s1, Figures S1–S45: HPLC chromatograms of the ethanol extract and solvent fractions of *Quercus acuta* fruits are shown in Figure S1. The ^1^H, ^13^C, HSQC, and HMBC NMR spectra of gallic acid (**1**) in methanol-d_4_ are presented in Figures S2–S5. The ^1^H and ^13^C NMR spectra of sinapaldehyde (**2**) in acetone-d_6_ are shown in Figures S6 and S7, and those of coniferaldehyde (**3**) in chloroform-d are displayed in Figures S8 and S9. Figures S10 and S11 present the ^1^H and ^13^C NMR spectra of 3,5,7,2′,6′-pentahydroxyflavanone (**4**) in acetone-d_6_. Figures S12–S17 include the ^1^H, ^13^C, HMQC, HMBC, DEPT, and COSY spectra of 1,2,3,6-tetrakis-*O*-galloyl-β-*D*-glucose (**5**) in methanol-d_4_. The ^1^H and ^13^C NMR spectra of phlorizin (**6**) in methanol-d_4_ are provided in Figures S18 and S19. Figures S20 and S21 show the ^1^H and ^13^C NMR spectra of 3,4-dimethoxycinnamic acid (**7**) in DMSO-d_6_, and Figures S22 and S23 display those of ferulic acid (**8**) in chloroform-*d*. Figures S24 and S25 present the ^1^H and ^13^C NMR spectra of taxifolin (**9**) in methanol-d_4_, while Figures S26 and S27 show those of catechin (**10**) in DMSO-d_6_. Figures S28–S31 contain the ^1^H, ^13^C, HMQC, and HMBC spectra of ellagic acid (**11**) in DMSO-d_6_. The ^1^H, ^13^C, HMQC, HMBC, and COSY spectra of protocatechuic acid (**12**) in methanol-d_4_ are presented in Figures S32–S36. Figures S37–S41 display the ^1^H, ^13^C, HMQC, HMBC, and COSY spectra of corilagin (**13**) in acetone-d_6_. Figures S42 and S43 show the ^1^H and ^13^C NMR spectra of 2,5-dihydroxybenzaldehyde (**14**) in acetone-d_6_. Figures S44 and S45 provide the HPLC chromatograms of the isolated compounds, with S44 shown for compounds **1**–**7** and S45 shown for compounds **8**–**14**.

## Abbreviations

The following abbreviations are used in this manuscript:

Bu	*n*-butanol
CC	Column chromatography
CF	Chloroform
COX-2	Cyclooxygenase-2
DAPI	4′,6-diamidino-2-phenylindole
DMSO	Dimethyl sulfoxide
DW	Deionized H_2_O
EA	Ethyl acetate
ELISA	Enzyme-linked immunosorbent assay
FBS	Fetal bovine serum
H-ESI	Heated electrospray ionization
HPLC	High-Performance Liquid Chromatography
HRP	horseradish peroxidase
Hx	*n*-hexane
iNOS	Inducible nitric oxide synthase
IL-6	Interleukin-6
LPS	Lipopolysaccharide
MPLC	Medium-pressure liquid chromatography
NF-κB	Nuclear factor kappa-light-chain-enhancer of activated B cells
NMR	Nuclear magnetic resonance
NO	Nitric oxide
*Q. acuta*	*Quercus acuta* Thunb.
RPMI-1640	Roswell Park Memorial Institute 1640 medium
RP-18	Reversed-phase C18
SiO_2_	Silica gel
TIC	Total ion current (chromatogram)
TNF-α	Tumor necrosis factor-alpha
HR-MS/MS	High resolution tandem mass spectrometry
