# Supplementary material for: Anti-Neuroinflammatory Effects of Compounds Isolated from Quercus acuta Thunb. Fruits via NF-κB Signaling Inhibition in BV2 Microglia
Source: Molecules. 2025 Nov 22;30(23):4514. doi: 10.3390/molecules30234514 (PMC12693635; doi:10.3390/molecules30234514)
Supplement: Supplementary file 1 [file molecules-30-04514-s001.zip › molecules-3965636-supplementary.pdf]

Supplementary data

Article

Anti-Neuroinflammatory Effects of Compounds Isolated from *Quercus acuta* Thunb. Fruits via NF-κB Signaling Inhibition in BV2 Microglia

Contents

Figure S1. HPLC chromatograms of the ethanol extract and solvent fractions of *Quercus acuta* fruits

Figure S2. ^{1}H-NMR spectrum (methanol-d_4) of gallic acid (**1**)

Figure S3. ^{13}C-NMR spectrum (methanol-d_4) of gallic acid (**1**)

Figure S4. HSQC spectrum (methanol-d_4) of gallic acid (**1**)

Figure S5. HMBC spectrum (methanol-d_4) of gallic acid (**1**)

Figure S6. ^{1}H-NMR spectrum (acetone-d_6) of sinapaldehyde (**2**)

Figure S7. ^{13}C-NMR spectrum (acetone-d_6) of sinapaldehyde (**2**)

Figure S8. ^{1}H-NMR spectrum (chloroform-d) of coniferaldehyde (**3**)

Figure S9. ^{13}C-NMR spectrum (chloroform-d) of coniferaldehyde (**3**)

Figure S10. ^{1}H-NMR spectrum (acetone-d_6) of 3,5,7,2',6'-pentahydroxyflavanone (**4**)

Figure S11. ^{13}C-NMR spectrum (acetone-d_6) of 3,5,7,2',6'-pentahydroxyflavanone (**4**)

Figure S12. ^{1}H-NMR spectrum (methanol-d_4) of 1,2,3,6-Tetrakis-*O*-galloyl-beta-*D*-glucose (**5**)

Figure S13. ^{13}C-NMR spectrum (methanol-d_4) of 1,2,3,6-Tetrakis-*O*-galloyl-beta-*D*-glucose (**5**)

Figure S14. HMQC spectrum (methanol-d_4) of 1,2,3,6-Tetrakis-*O*-galloyl-beta-*D*-glucose (**5**)

Figure S15. HMBC spectrum (methanol-d_4) of 1,2,3,6-Tetrakis-*O*-galloyl-beta-*D*-glucose (**5**)

Figure S16. DEPT spectrum (methanol-d_4) of 1,2,3,6-Tetrakis-*O*-galloyl-beta-*D*-glucose (**5**)

Figure S17. COSY spectrum (methanol-d_4) of 1,2,3,6-Tetrakis-*O*-galloyl-beta-*D*-glucose (**5**)

Figure S18. ^{1}H-NMR spectrum (methanol-d_4) of Phlorizin (**6**)

Figure S19. ^{13}C-NMR spectrum (methanol-d_4) of Phlorizin (**6**)

Figure S20. ^{1}H-NMR spectrum (DMSO-d_6) of 3,4-dimethoxycinnamic acid (**7**)

Figure S21. ^{13}C-NMR spectrum (DMSO-d_6) of 3,4-dimethoxycinnamic acid (**7**)

Figure S22. ^{1}H-NMR spectrum (chloroform-d) of ferulic acid (**8**)

Figure S23. ^{13}C-NMR spectrum (chloroform-d) of ferulic acid (**8**)

Figure S24. ^{1}H-NMR spectrum (methanol-d_4) of taxifolin (**9**)

Figure S25. ^{13}C-NMR spectrum (methanol-d_4) of taxifolin (**9**)

Figure S26. ^{1}H-NMR spectrum (DMSO-d_6) of catechin (**10**)

Figure S27. ^{13}C-NMR spectrum (DMSO-d_6) of catechin (**10**)

Figure S28. ^{1}H-NMR spectrum (DMSO-d_6) of ellagic acid (**11**)

Figure S29. ^{13}C-NMR spectrum (DMSO-d_6) of ellagic acid (**11**)

Figure S30. HMQC spectrum (DMSO-d_6) of ellagic acid (**11**)

Figure S31. HMBC spectrum (DMSO-d_6) of ellagic acid (**11**)

Figure S32. ^{1}H-NMR spectrum (methanol-d_4) of protocatechuic acid (**12**)

Figure S33. ^{13}C-NMR spectrum (methanol-d_4) of protocatechuic acid (**12**)

Figure S34. HMQC spectrum (methanol-d_4) of protocatechuic acid (**12**)

Figure S35. HMBC spectrum (methanol-d_4) of protocatechuic acid (**12**)

Figure S36. COSY spectrum (methanol-d_4) of protocatechuic acid (**12**)

Figure S37. ^{1}H-NMR spectrum (acetone-d_6) of corilagin (**13**)

Figure S38. ^{13}C-NMR spectrum (acetone-d_6) of corilagin (**13**)

Figure S39. HMQC spectrum (acetone-d_6) of corilagin (**13**)

Figure S40. HMBC spectrum (acetone-d_6) of corilagin (**13**)

Figure S41. COSY spectrum (acetone-d_6) of corilagin (**13**)

Figure S42. ^{1}H-NMR spectrum (acetone-d_6) of 2,5-dihydroxybenzaldehyde (**14**)

Figure S43. ^{13}C-NMR spectrum (acetone-d_6) of 2,5-dihydroxybenzaldehyde (**14**)

Figure S44. HPLC chromatograms of compounds **1–7** isolated from the ethanol extract of *Quercus acuta* fruits

Figure S45. HPLC chromatograms of compounds **8–14** isolated from the ethanol extract of *Quercus acuta* fruits

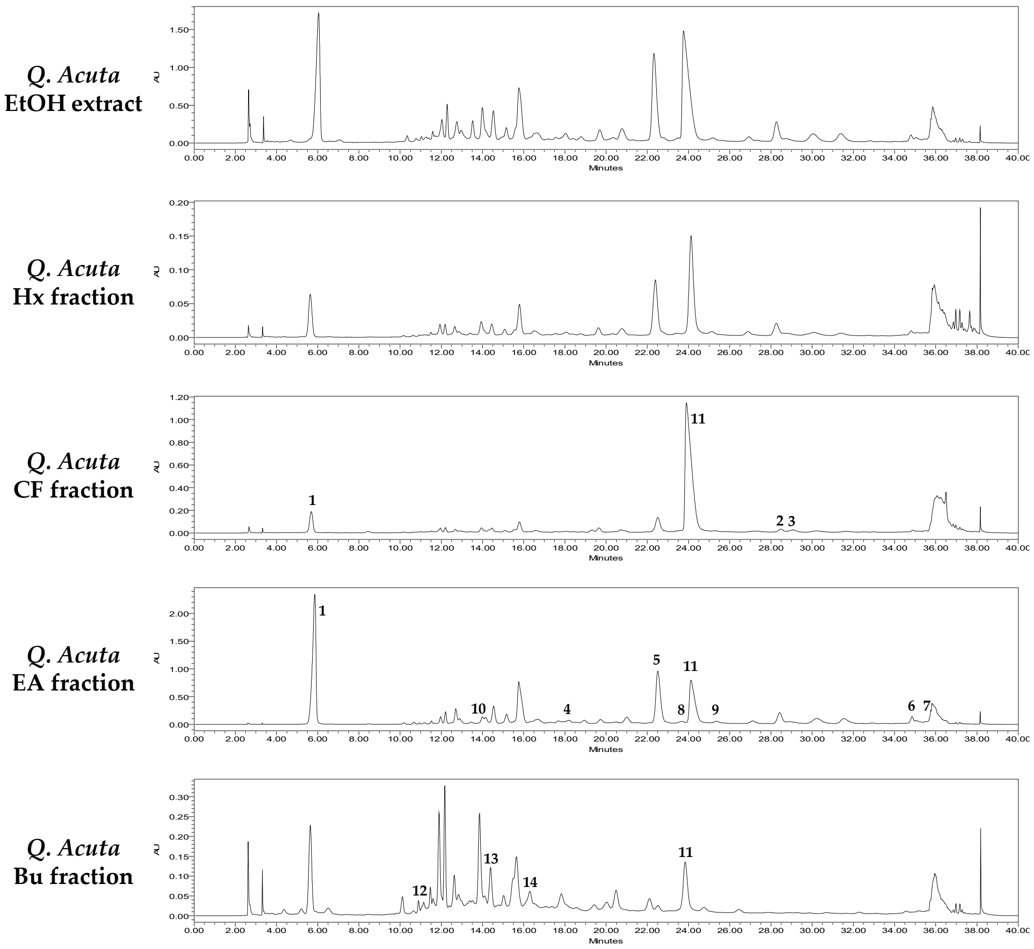

Figure S1. HPLC chromatograms of the ethanol extract and solvent fractions of *Quercus acuta* fruits. Analyses were performed using an Agilent Eclipse XDB-C18 column (4.6 × 250 mm, 5 μm). The mobile phases consisted of solvent A (0.1% formic acid in water) and solvent B (acetonitrile) with the following gradient system: 0.0–5.0 min, 5% B; 5.0–20.0 min, 5–30% B; 20.0–28.0 min, 30–50% B; 28.0–32.0 min, 50–95% B; 32.0–36.0 min, 95% B; 36.0–40.0 min, 95–5% B. The flow rate was 1.0 mL/min, the injection volume was 10 μL, and detection was performed at 254 nm.

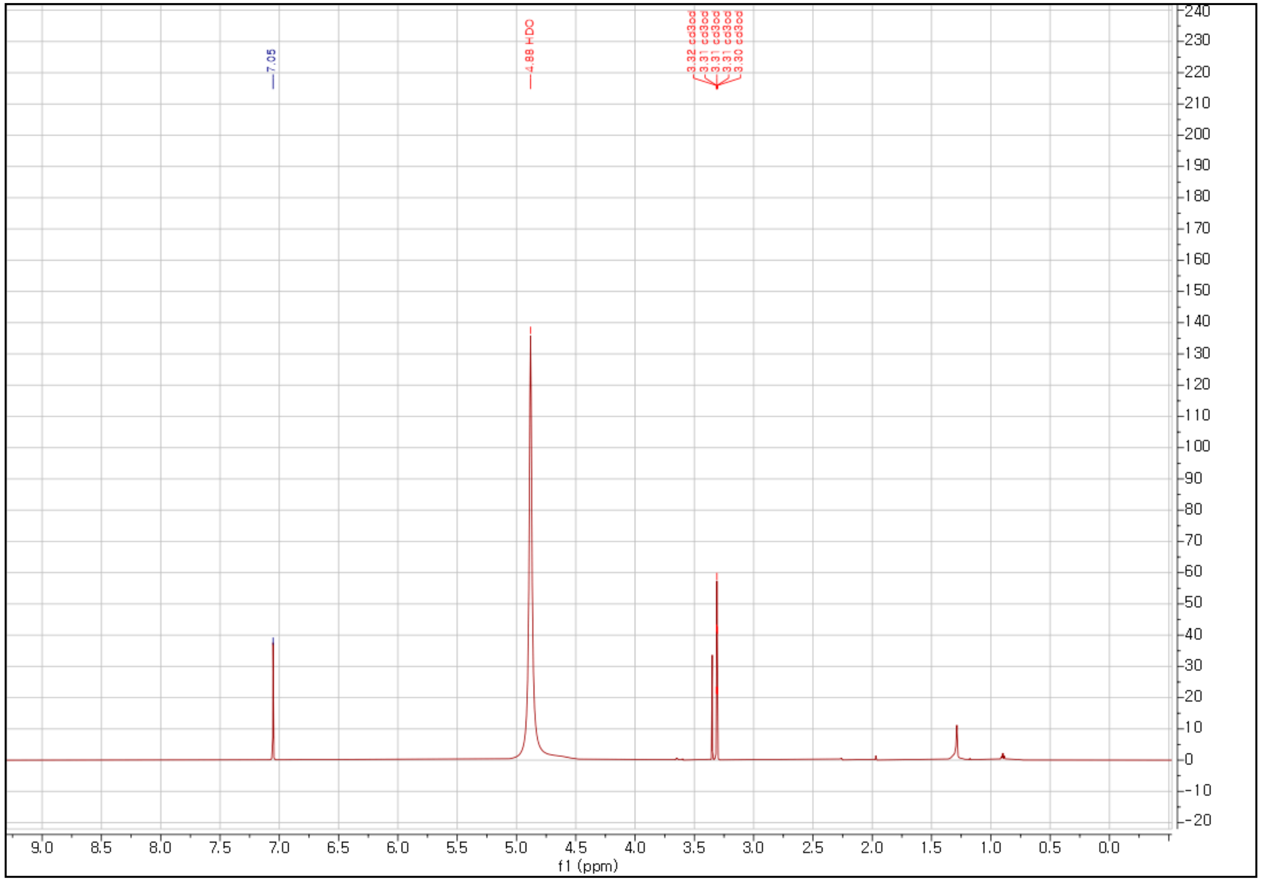

Figure S2. ^{1}H-NMR spectrum (methanol-d_4) of gallic acid (**1**)

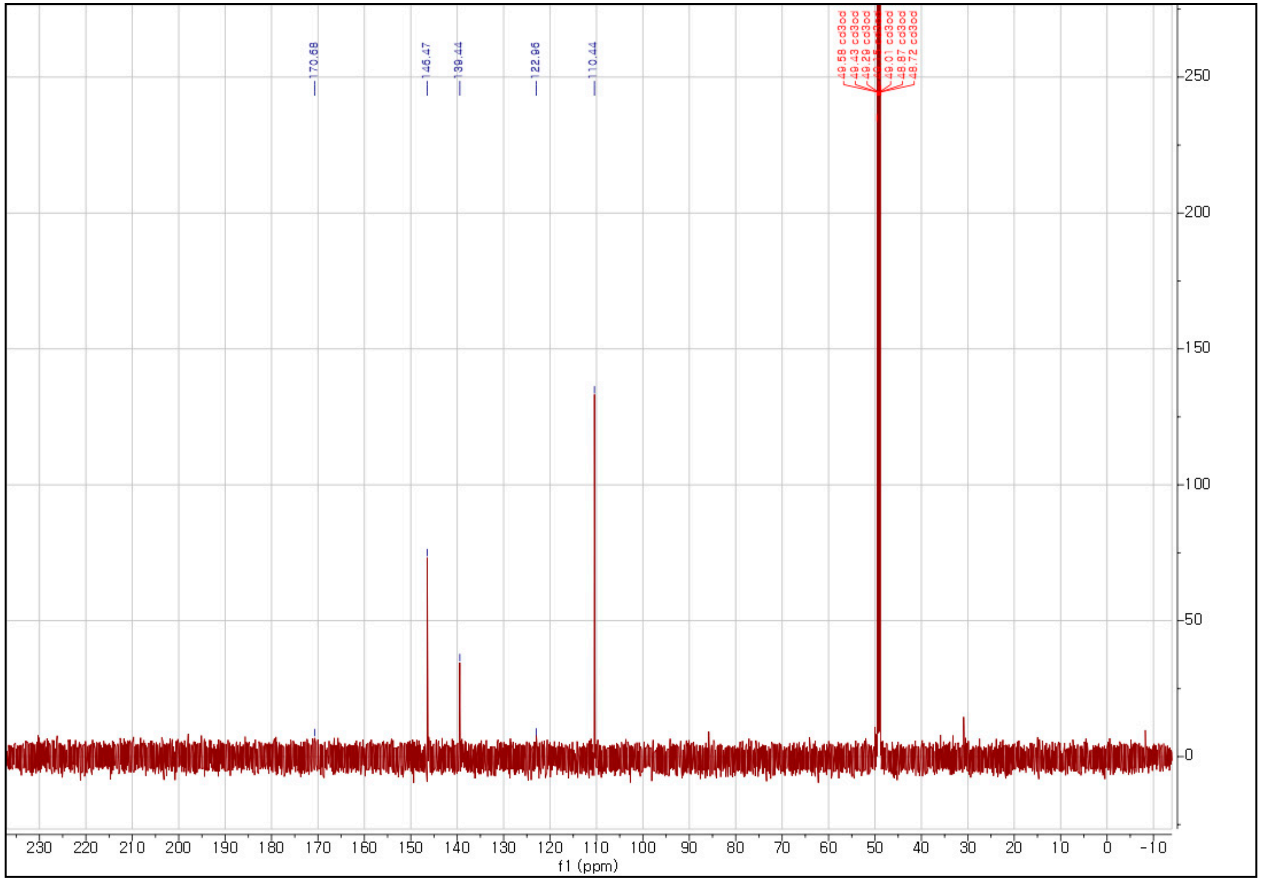

Figure S3. ^{13}C-NMR spectrum (methanol-d_4) of gallic acid (**1**)

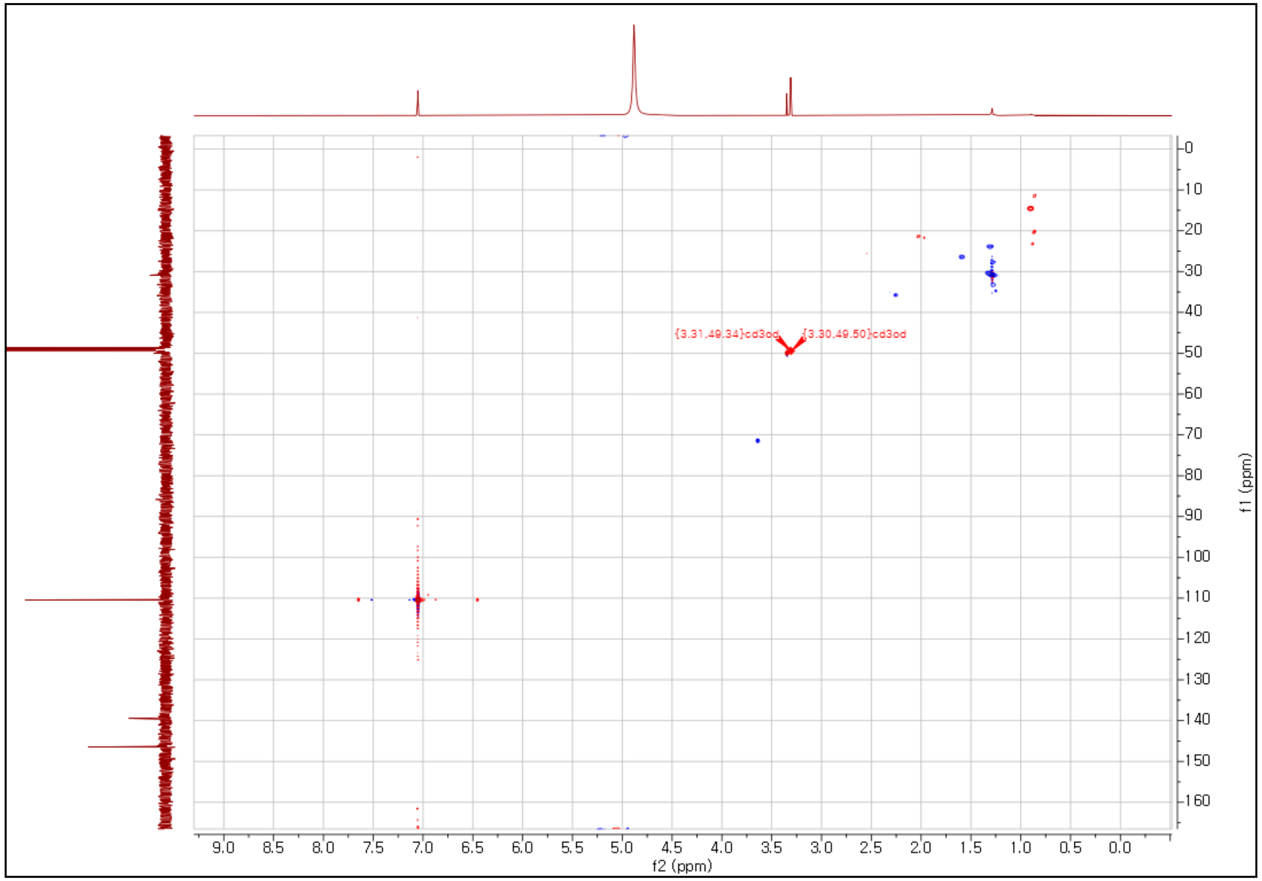

Figure S4. HSQC spectrum (methanol-d4) of gallic acid (**1**)

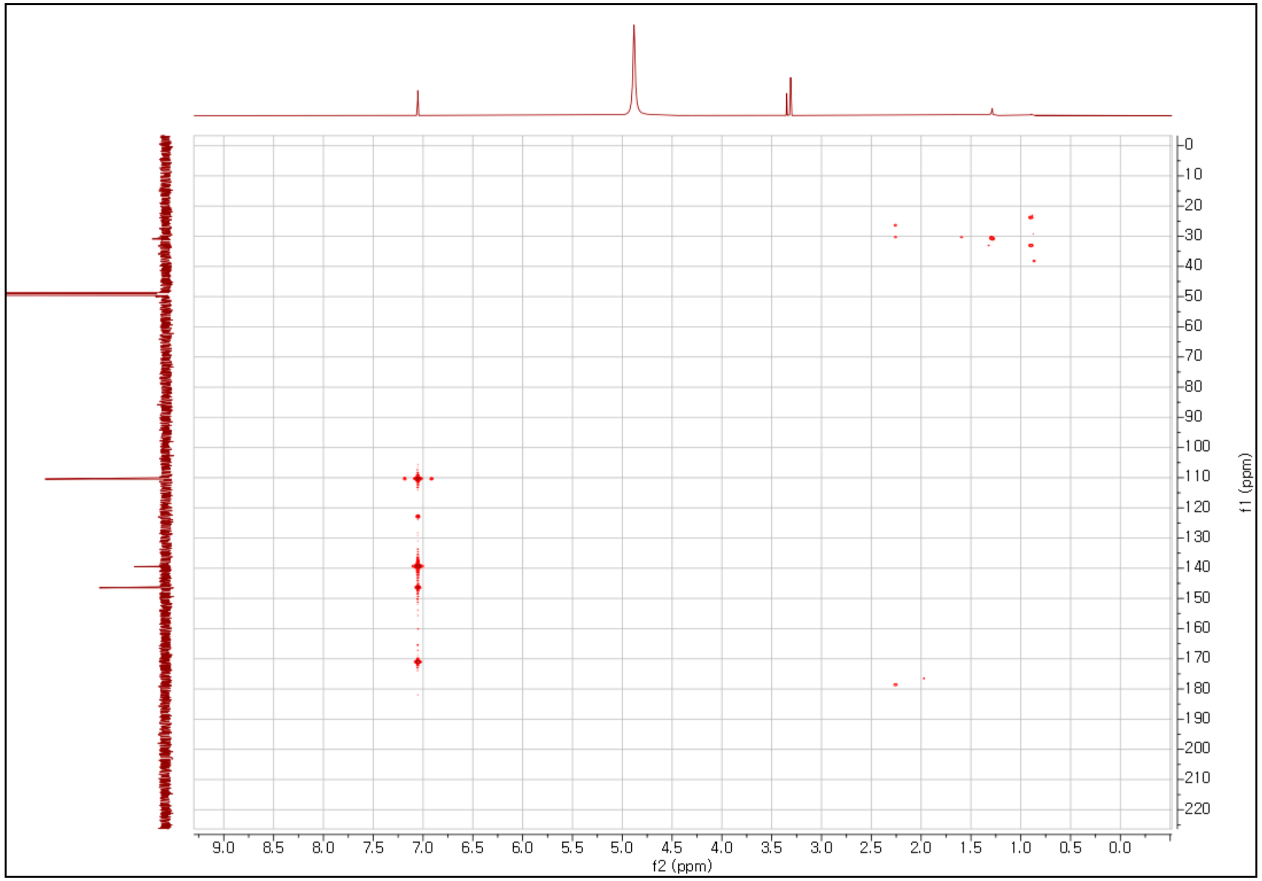

Figure S5. HMBC spectrum (methanol-d4) of gallic acid (**1**)

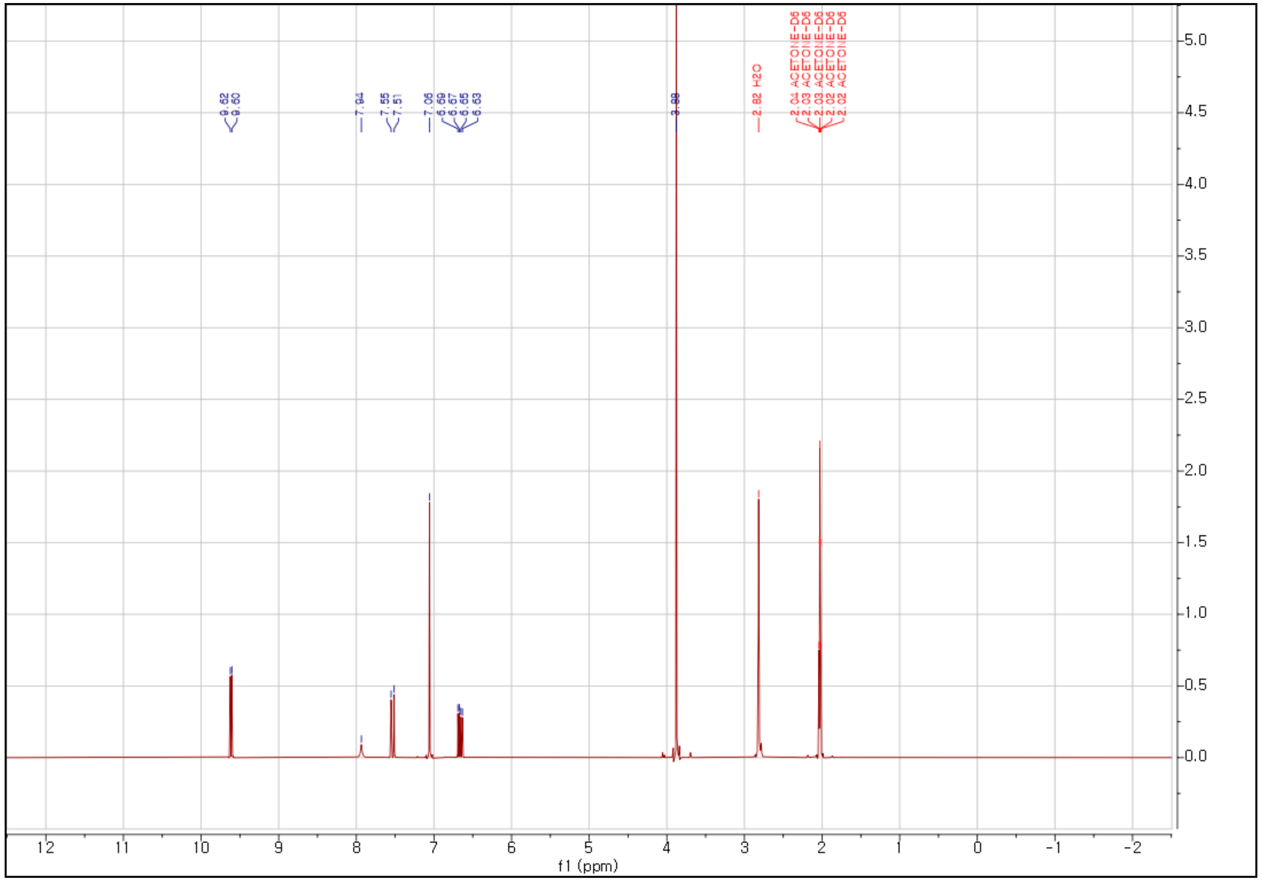

Figure S6. ^{1}H-NMR spectrum (acetone-d_6) of sinapaldehyde (**2**)

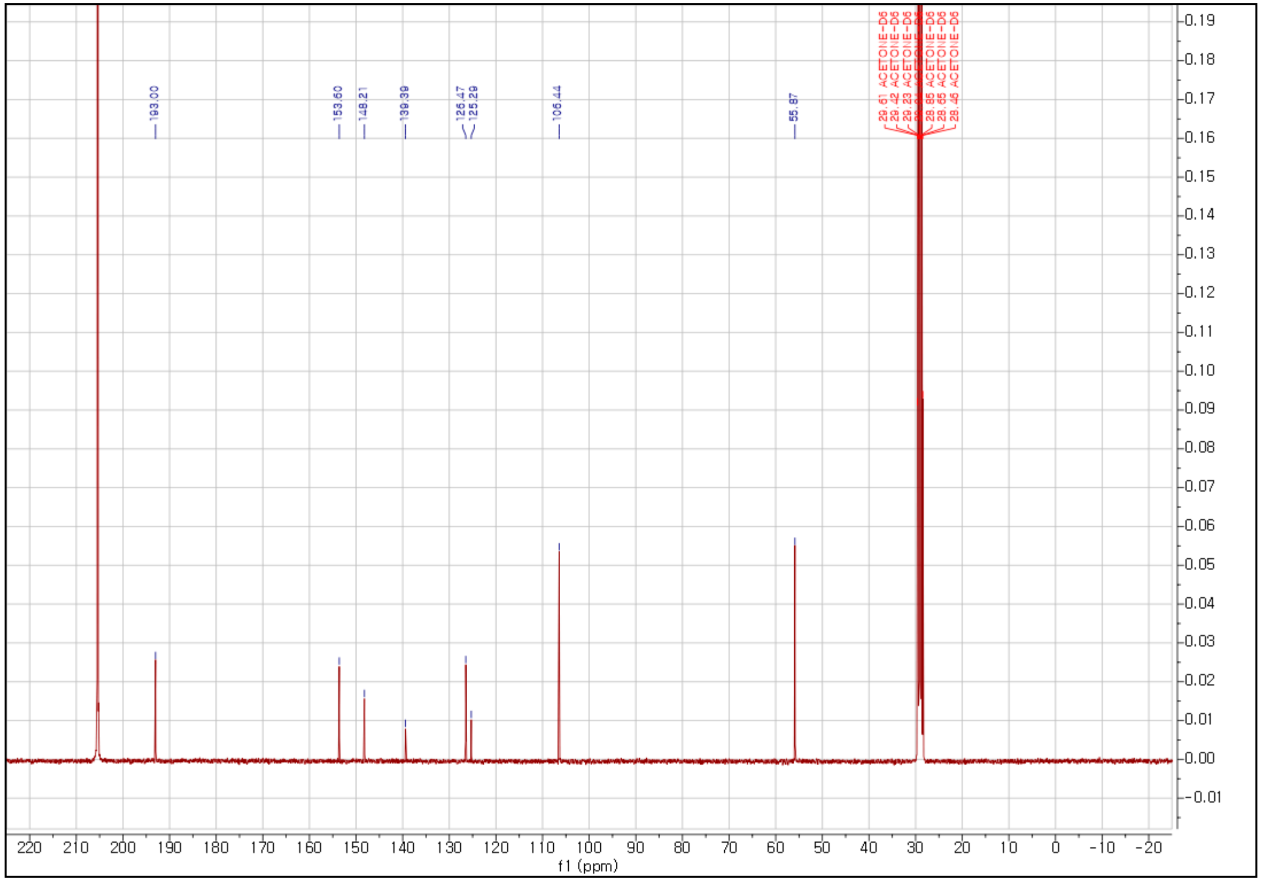

Figure S7. ^{13}C-NMR spectrum (acetone-d$_6$) of sinapaldehyde (**2**)

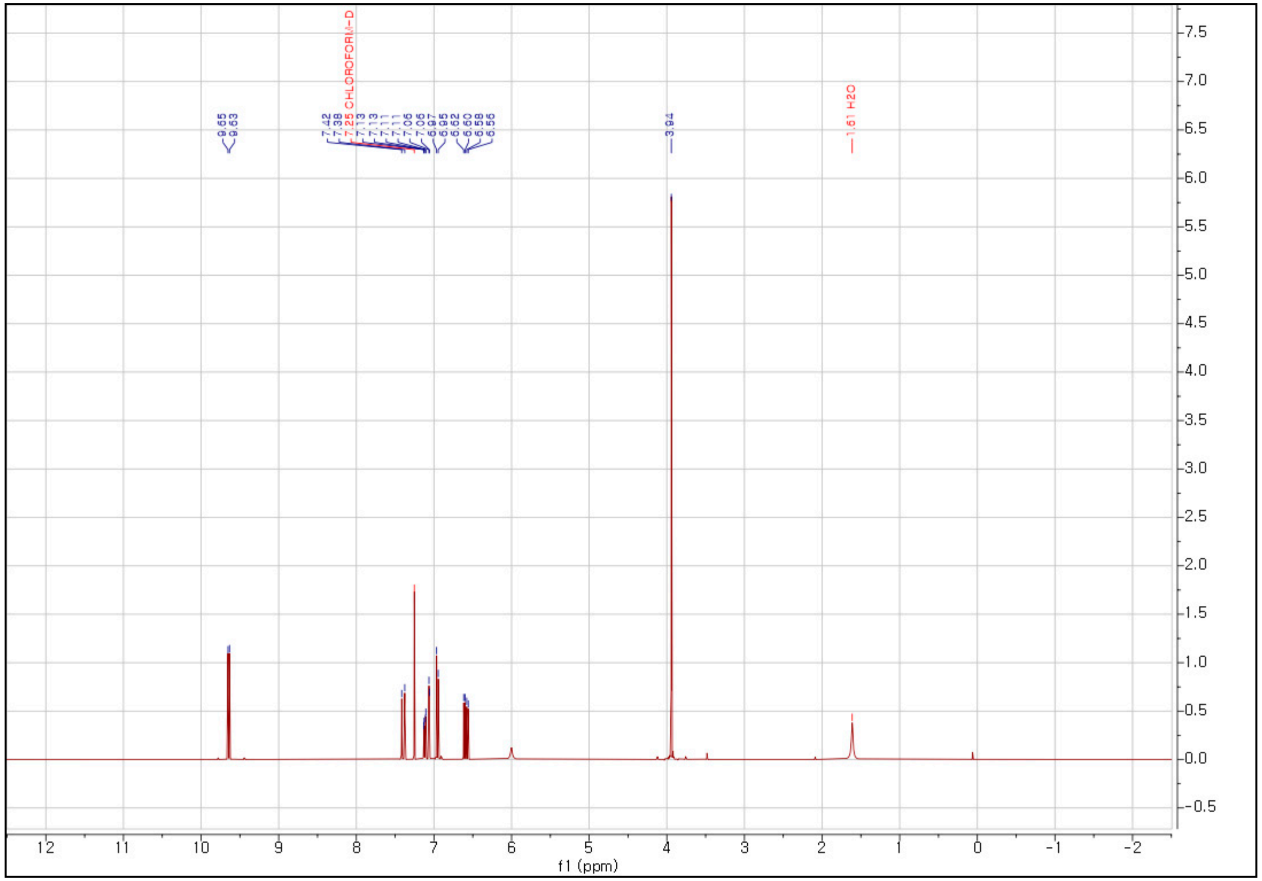

Figure S8. ^{1}H-NMR spectrum (chloroform-d) of coniferaldehyde (**3**)

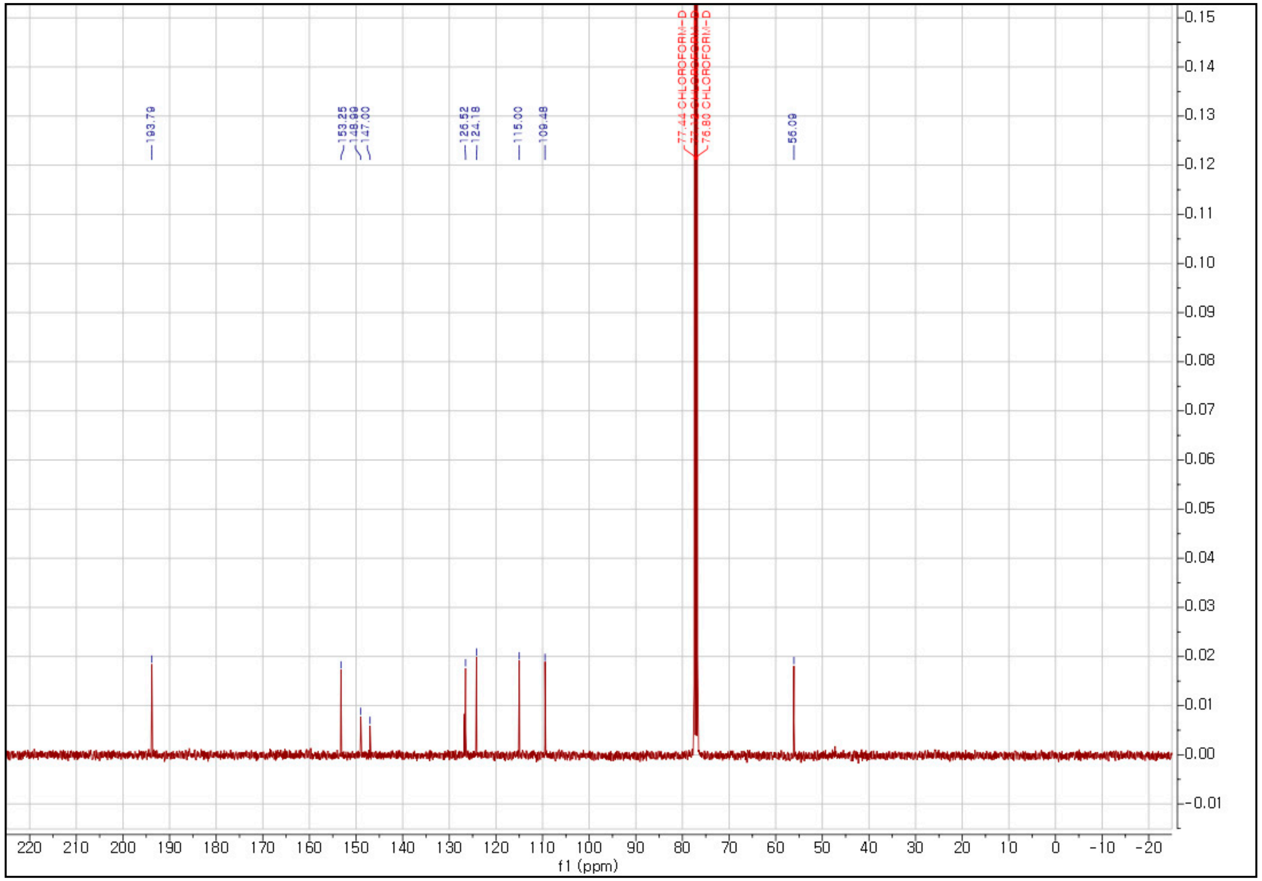

Figure S9. ^{13}C-NMR spectrum (chloroform-d) of coniferaldehyde (**3**)

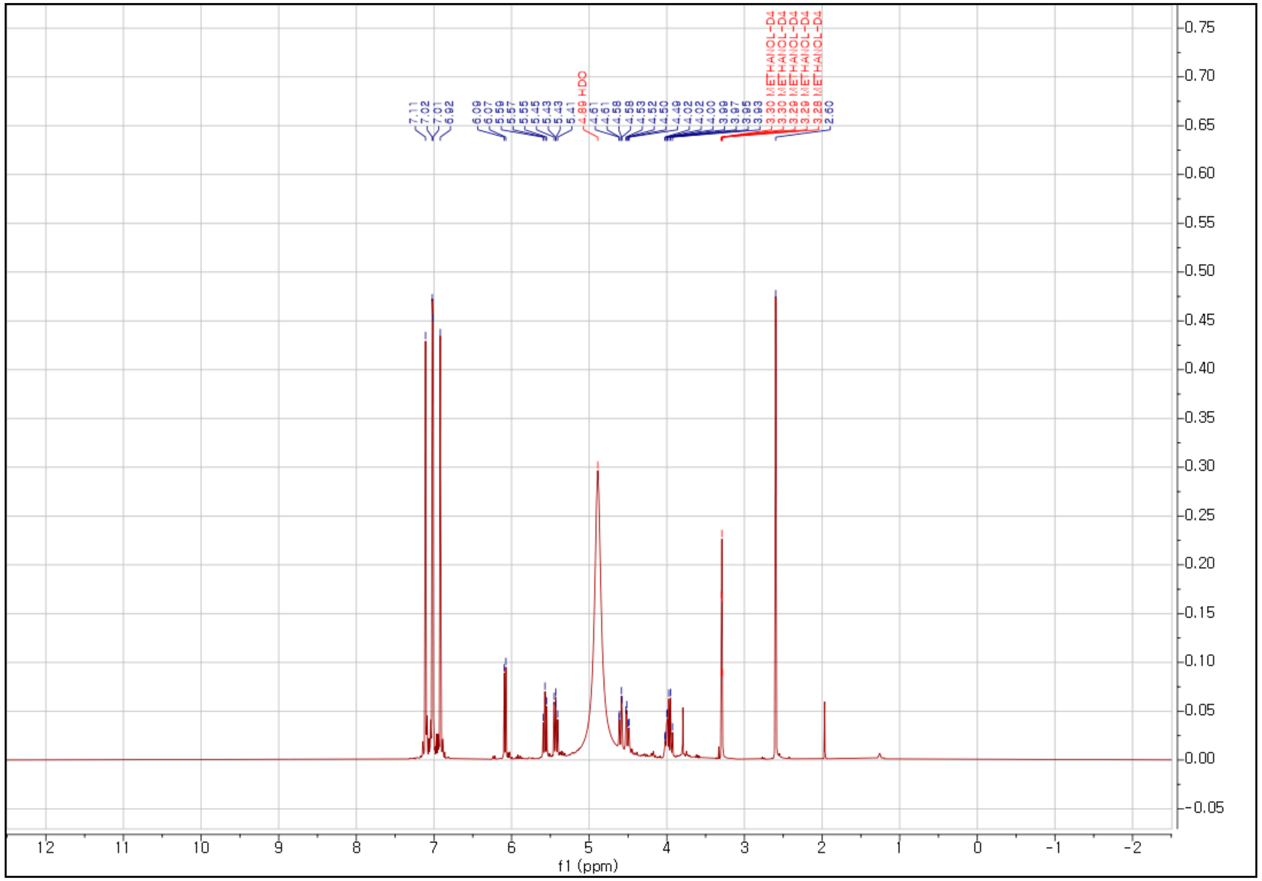

Figure S10. ^{1}H-NMR spectrum (acetone-d$_6$) of 3,5,7,2',6'-pentahydroxyflavanone (**4**)

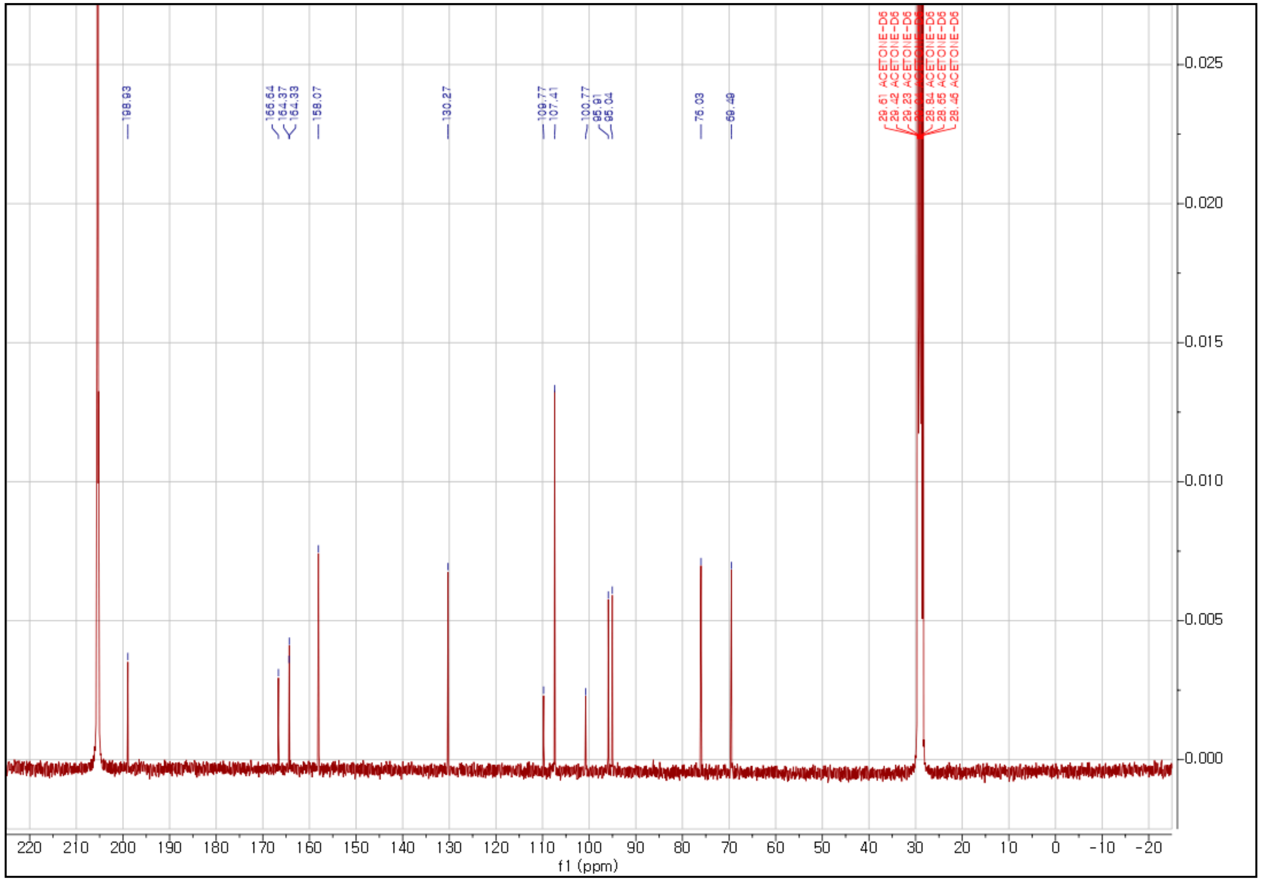

Figure S11. ^{13}C-NMR spectrum (acetone-d_6) of 3,5,7,2',6'-pentahydroxyflavanone (**4**)

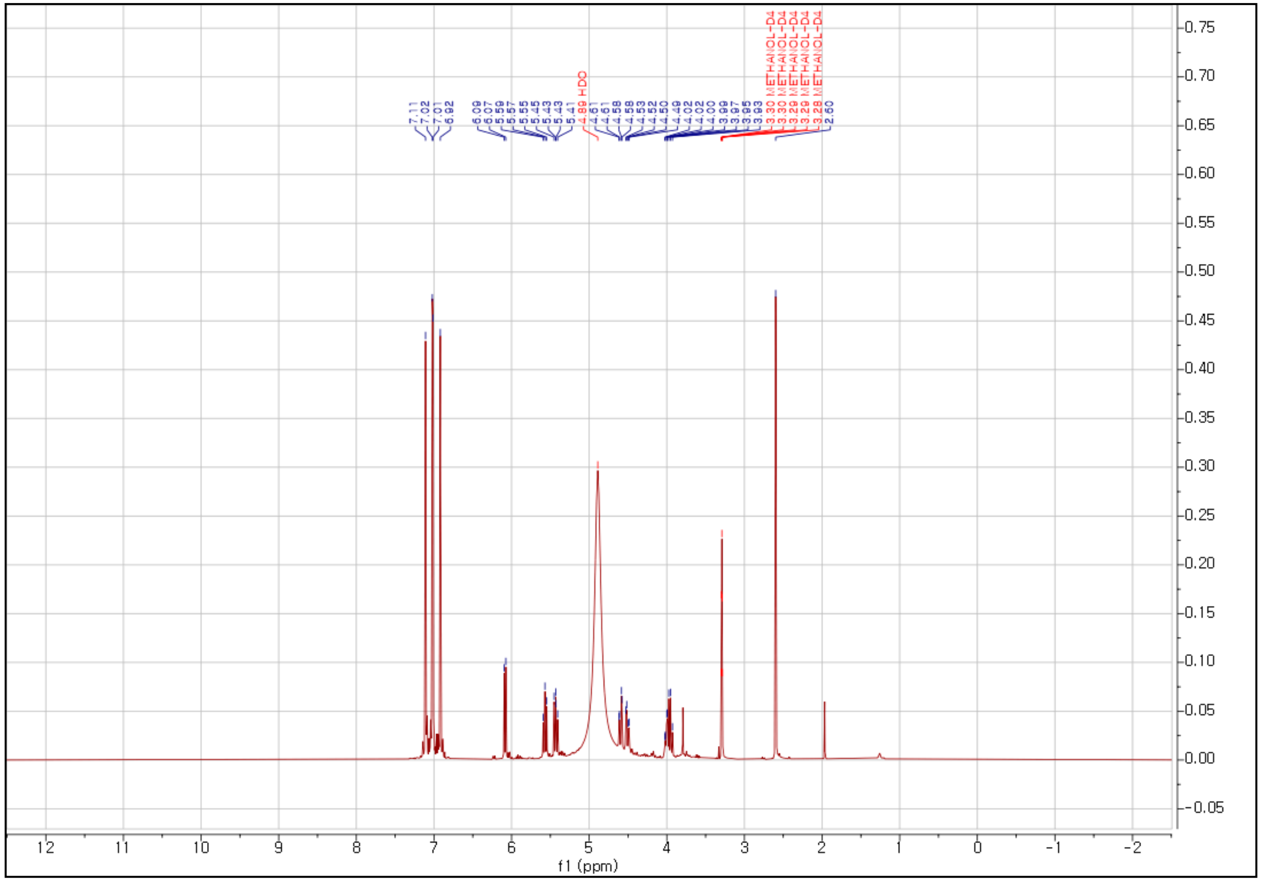

Figure S12. ^{1}H-NMR spectrum (methanol-d_4) of 1,2,3,6-Tetrakis-*O*-galloyl-beta-*D*-glucose (**5**)

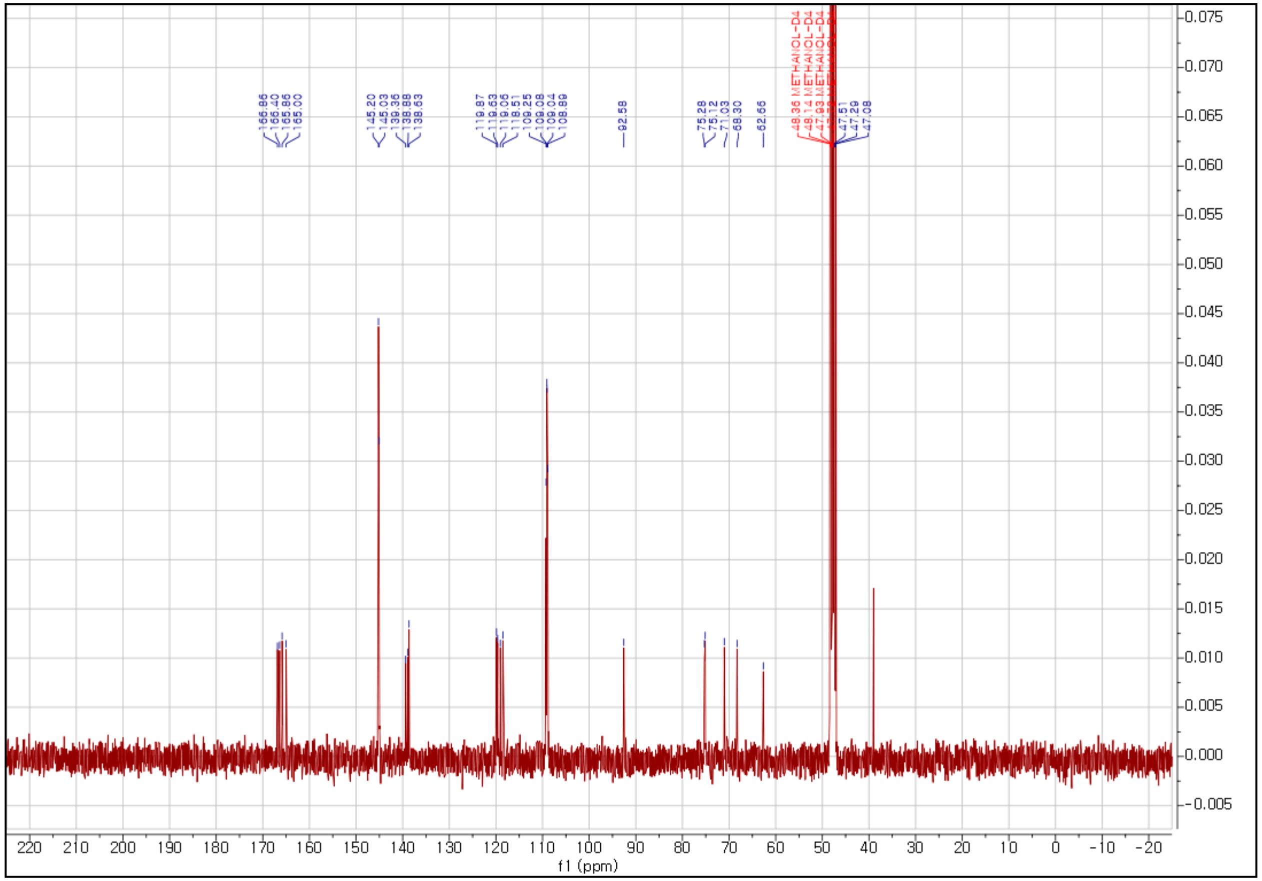

Figure S13. ^{13}C-NMR spectrum (methanol-d$_4$) of 1,2,3,6-Tetrakis-*O*-galloyl-beta-*D*-glucose (**5**)

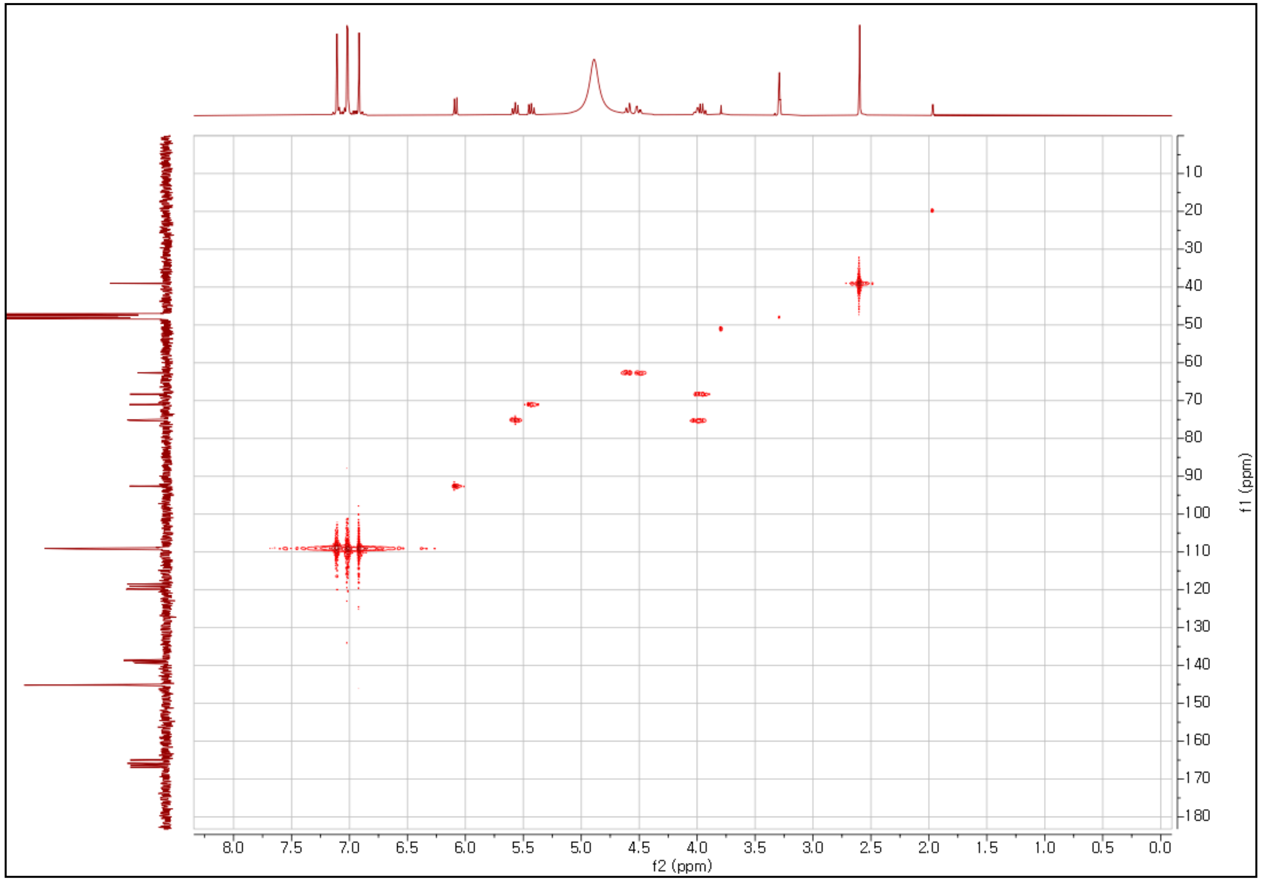

Figure S14. HMQC spectrum (methanol-d$_4$) of 1,2,3,6-Tetrakis-*O*-galloyl-beta-*D*-glucose (**5**)

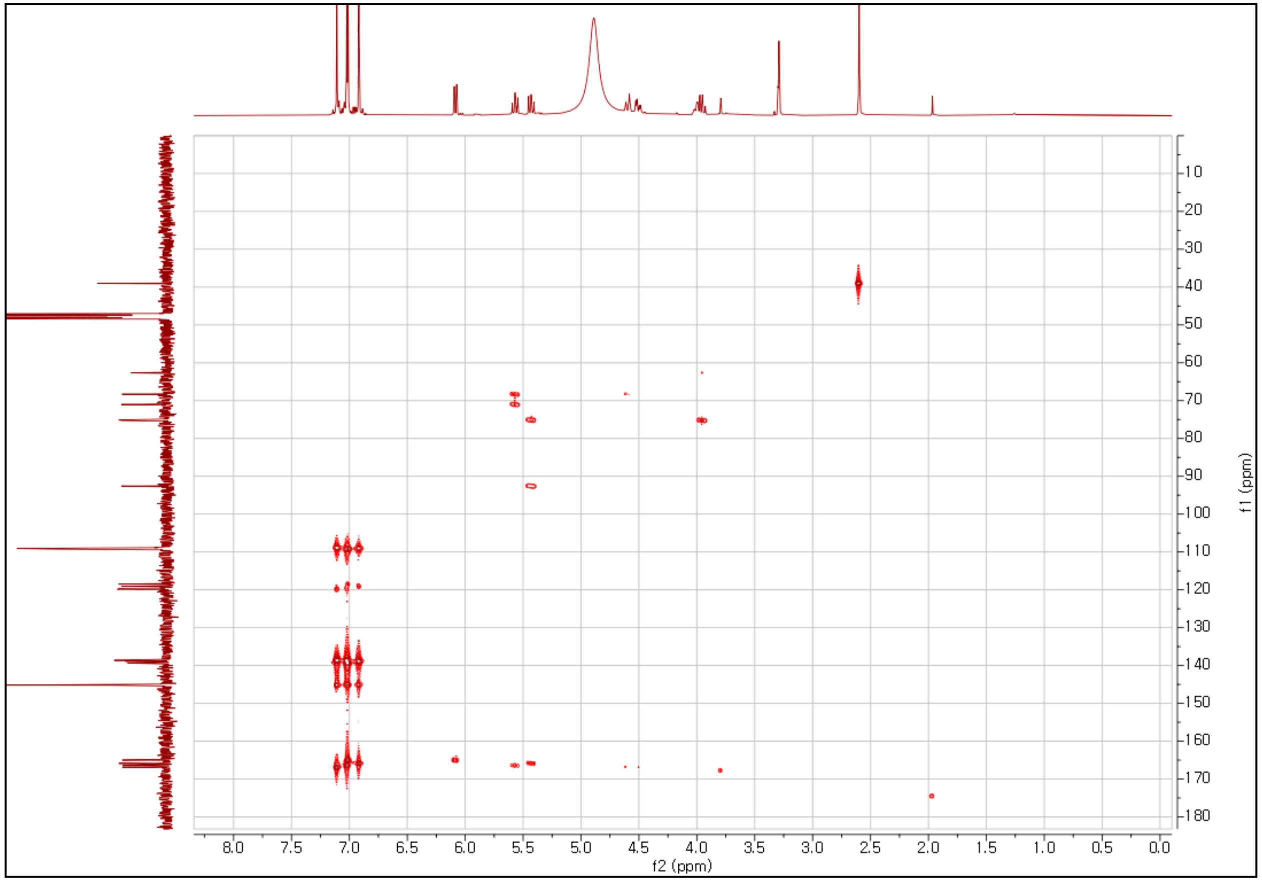

Figure S15. HMBC spectrum (methanol-d4) of 1,2,3,6-Tetrakis-*O*-galloyl-beta-*D*-glucose (**5**)

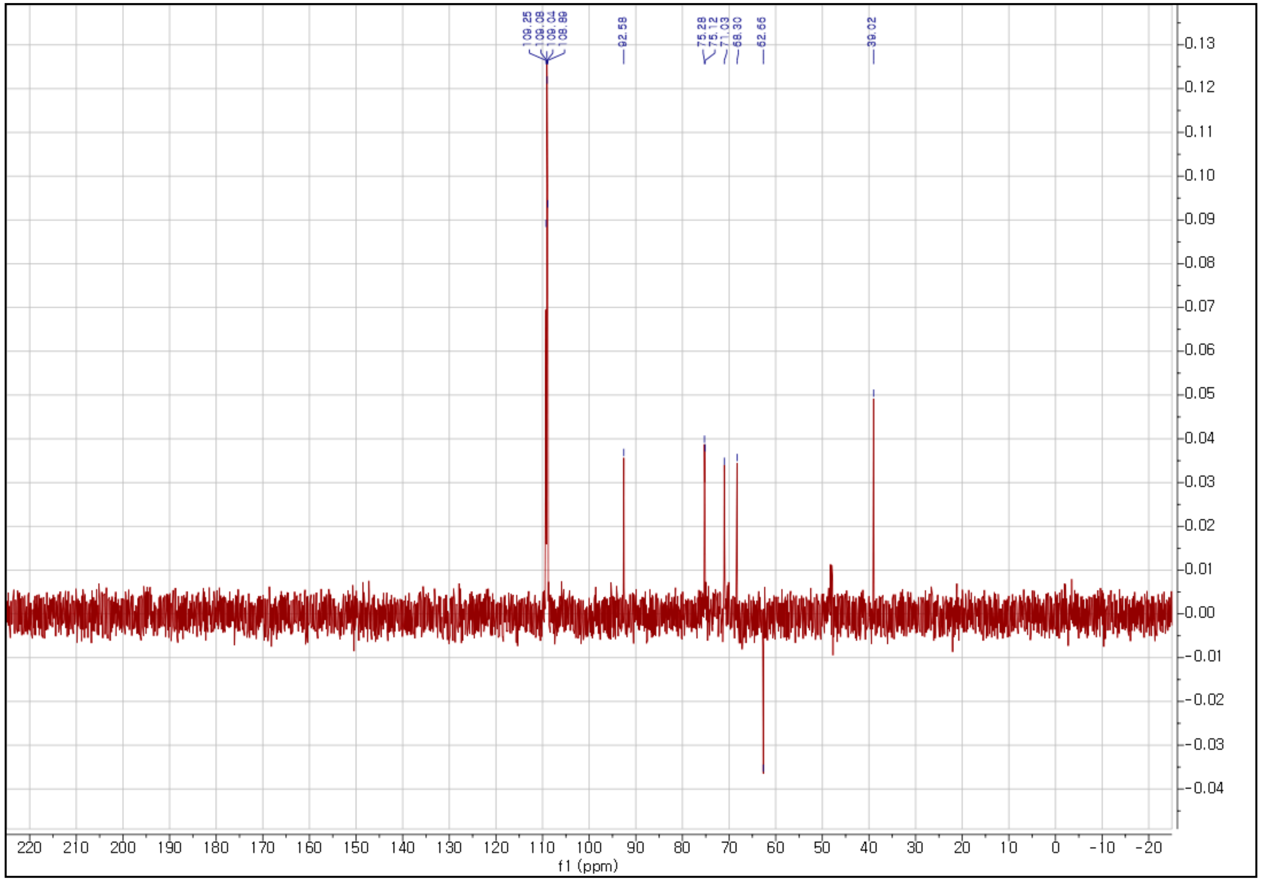

Figure S16. DEPT spectrum (methanol-d4) of 1,2,3,6-Tetrakis-*O*-galloyl-beta-*D*-glucose (**5**)

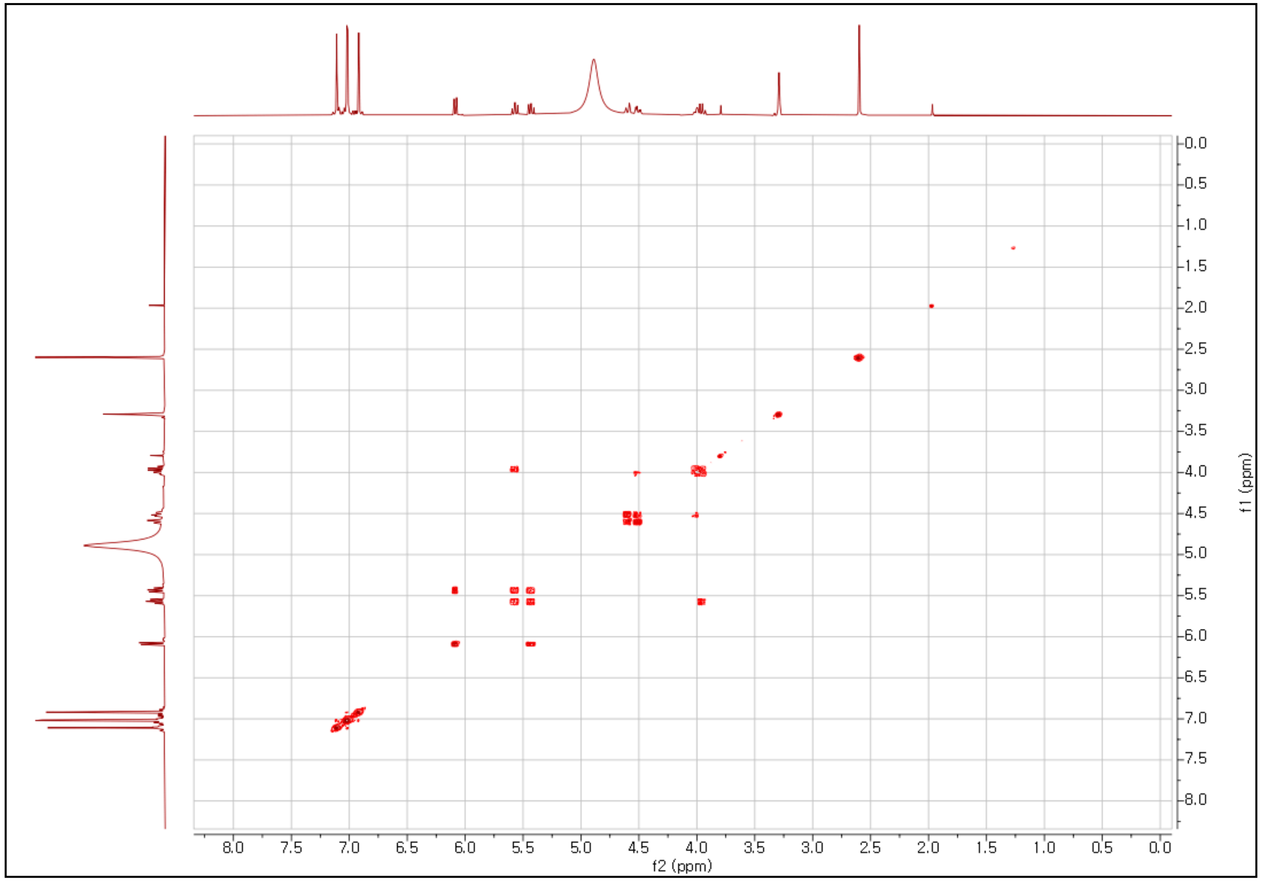

Figure S17. COSY spectrum (methanol-d$_4$) of 1,2,3,6-Tetrakis-*O*-galloyl-beta-*D*-glucose (**5**)

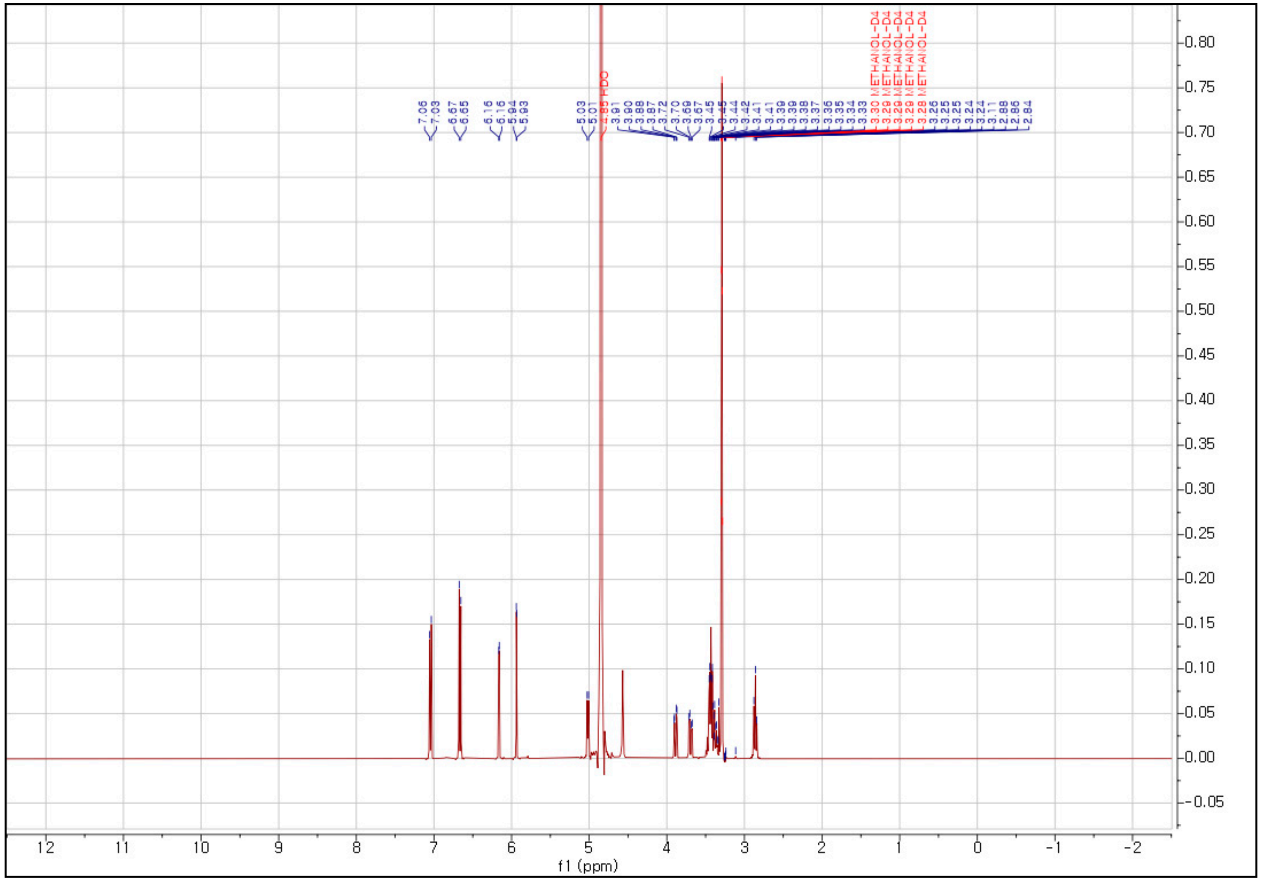

Figure S18. ^{1}H-NMR spectrum (methanol-d4) of Phlorizin (**6**)

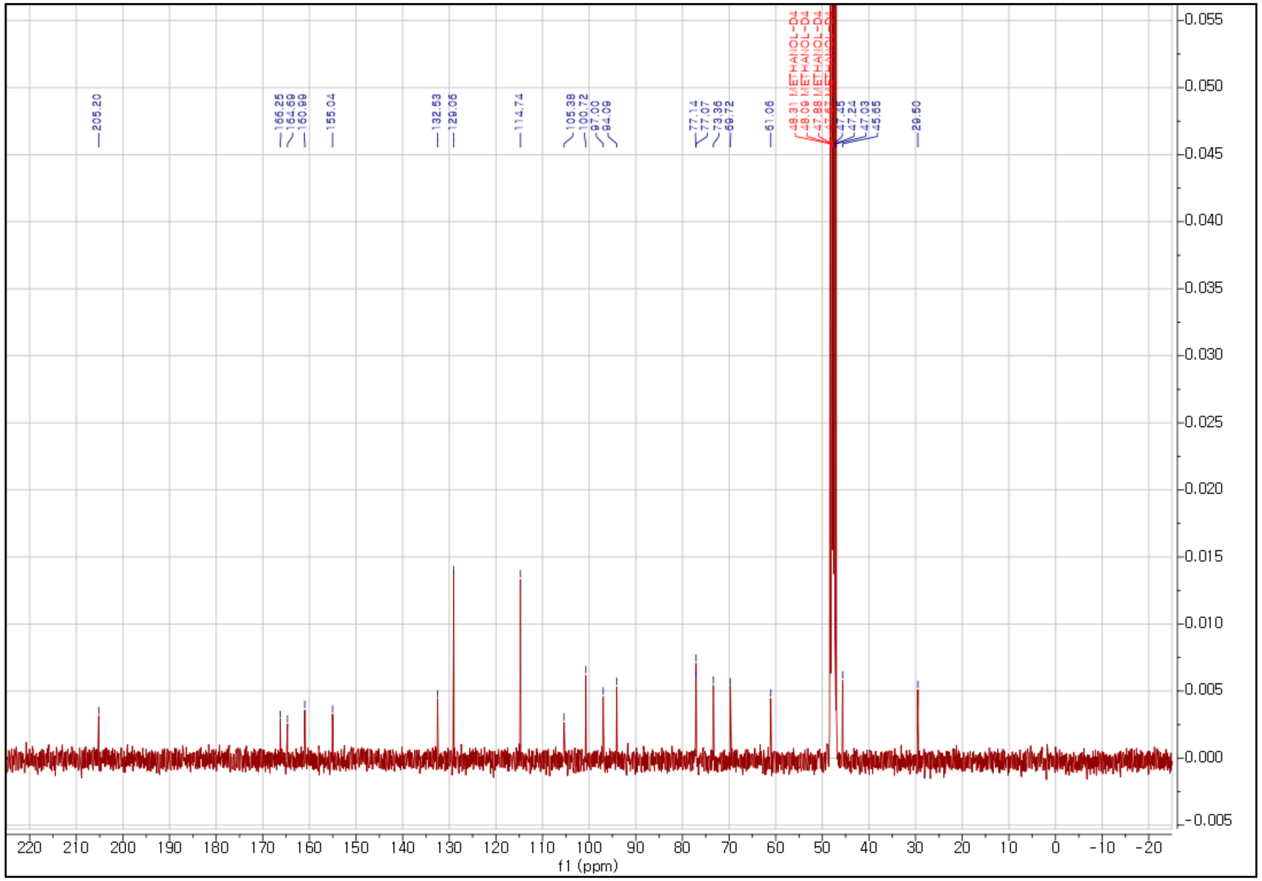

Figure S19. ^{13}C-NMR spectrum (methanol-d$_4$) of Phlorizin (**6**)

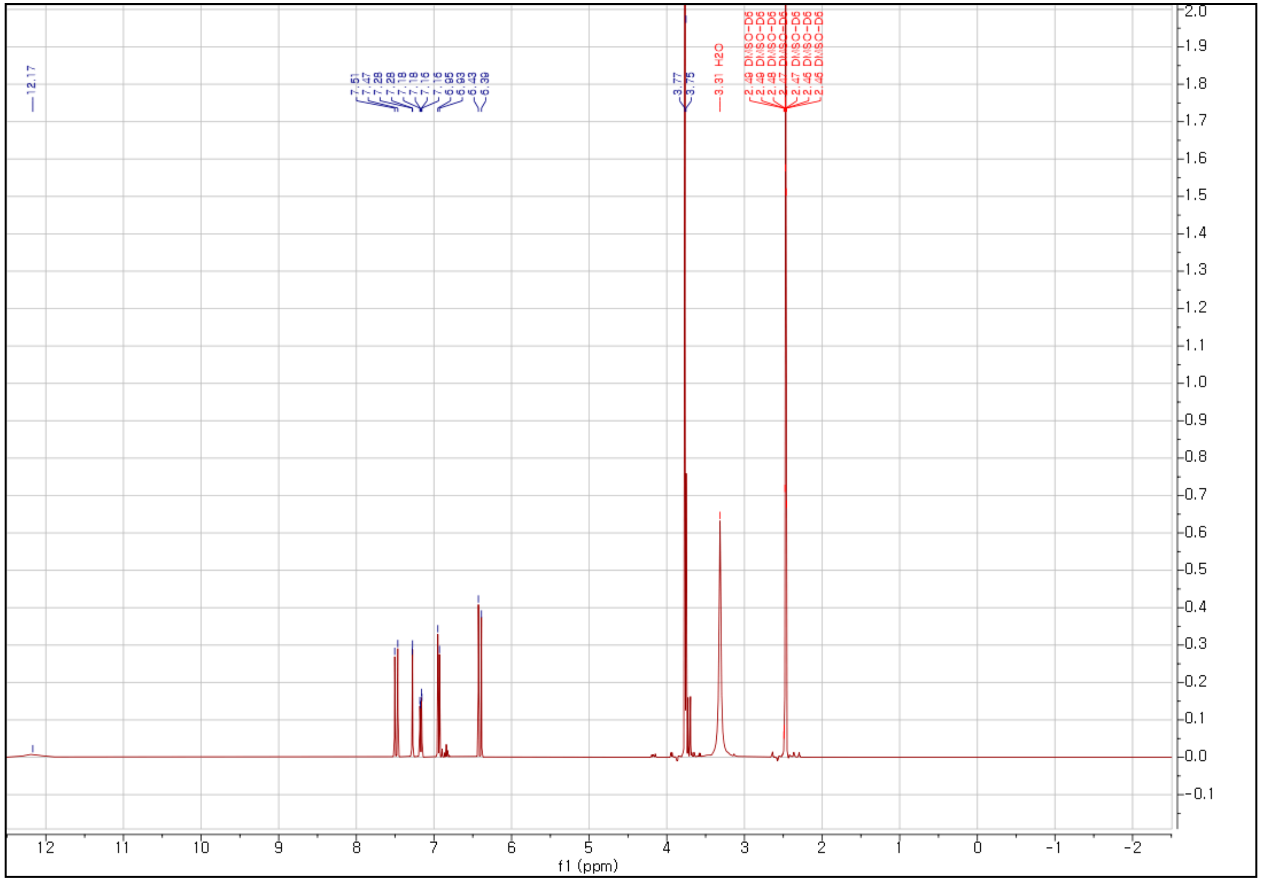

Figure S20. ^{1}H-NMR spectrum (DMSO-d$_6$) of 3,4-dimethoxycinnamic acid (**7**)

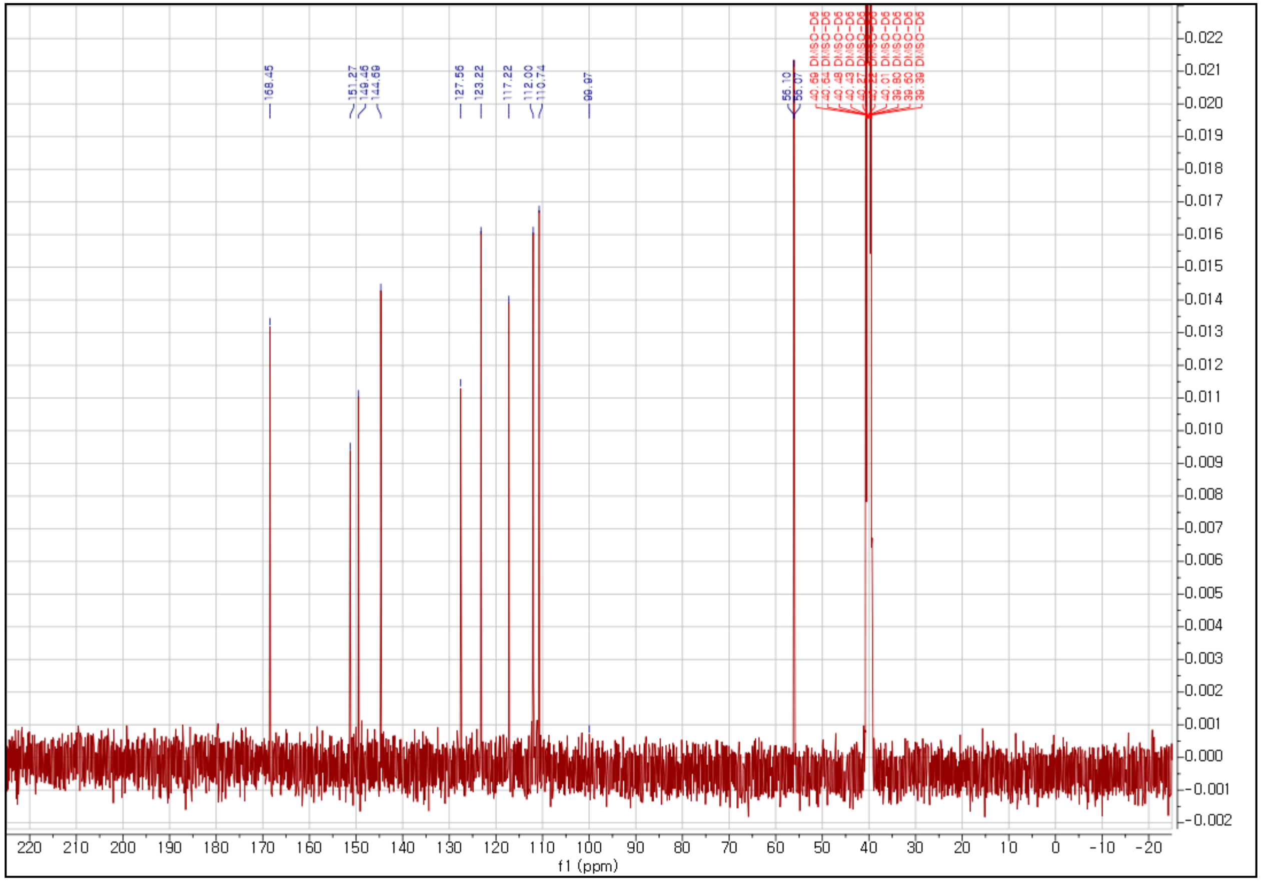

Figure S21. ^{13}C-NMR spectrum (DMSO-d_6) of 3,4-dimethoxycinnamic acid (**7**)

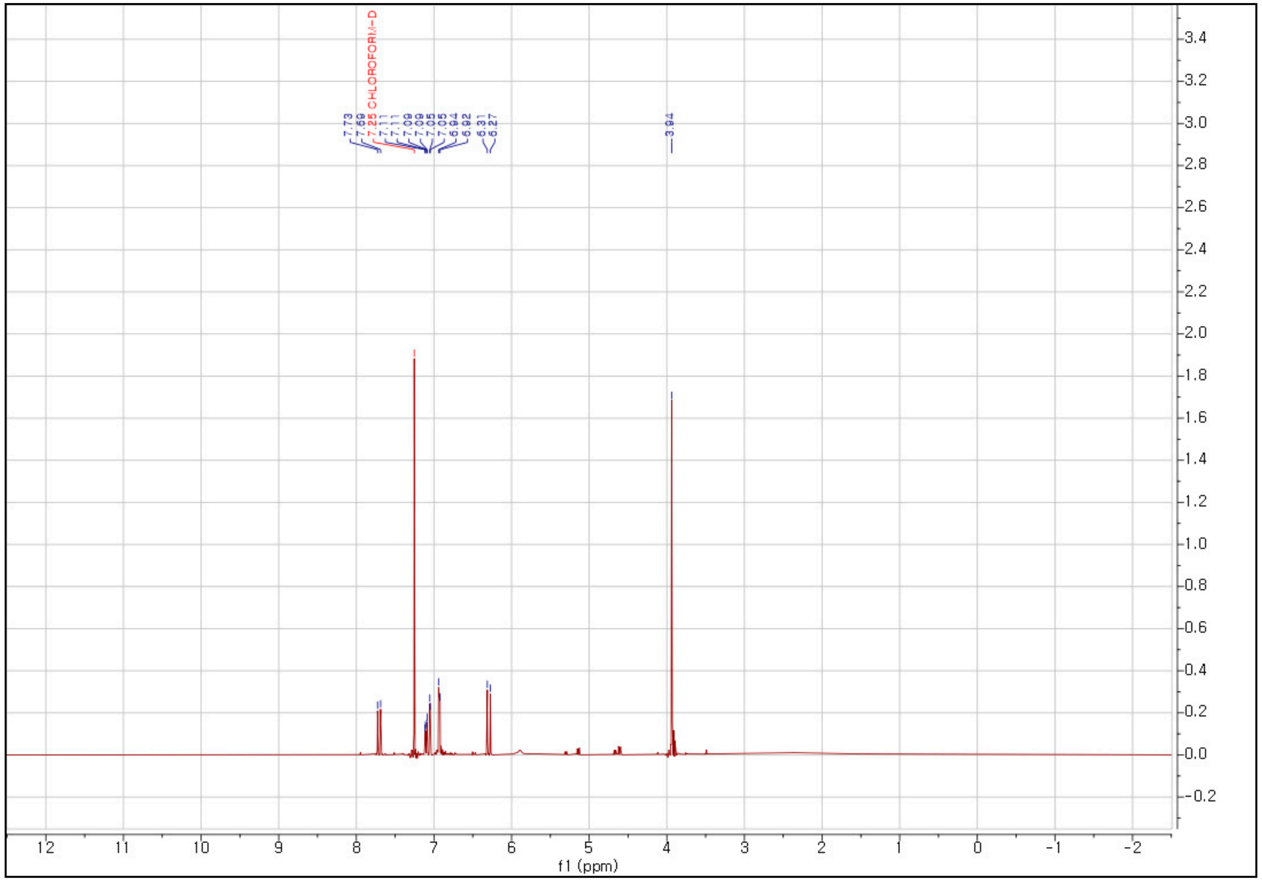

Figure S22. ^{1}H-NMR spectrum (chloroform-d) of ferulic acid (**8**)

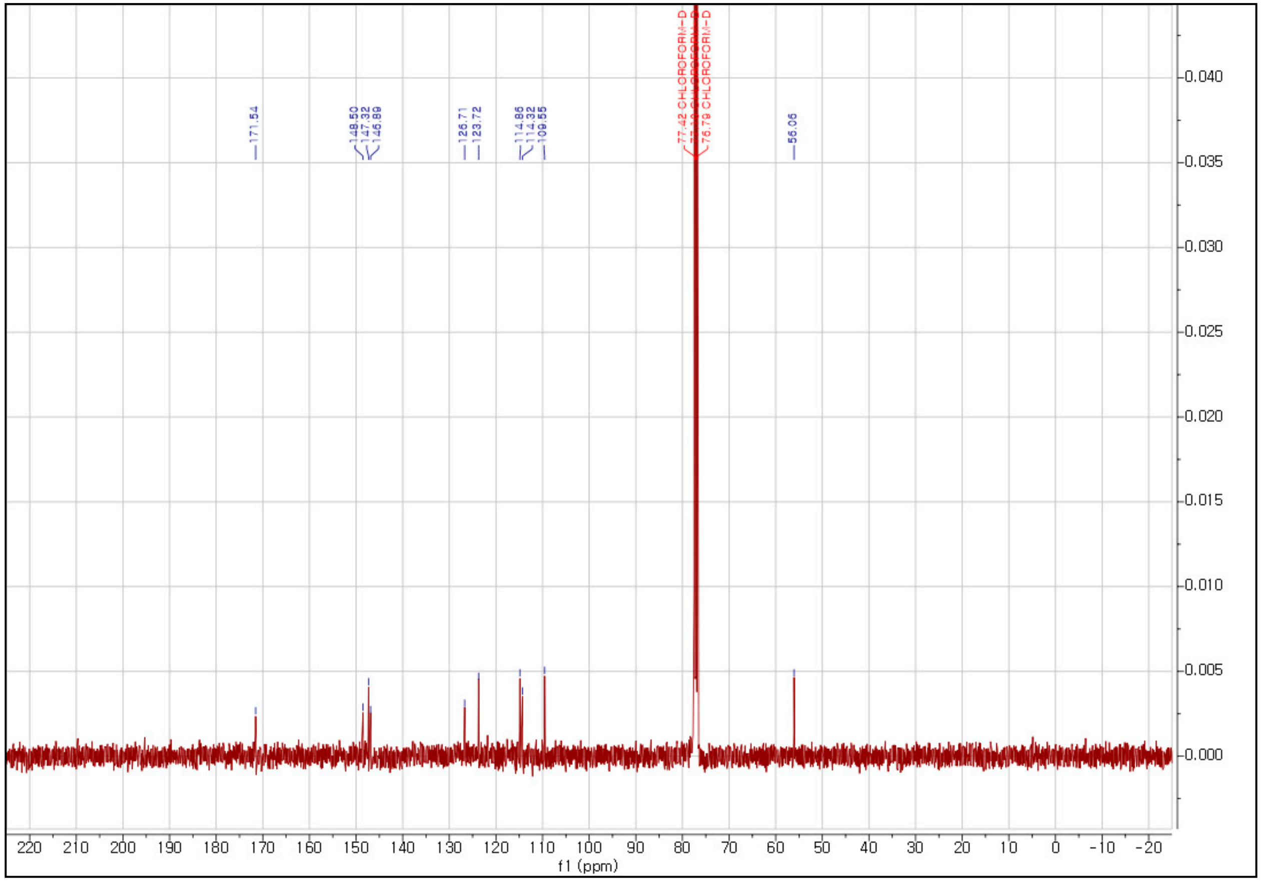

Figure S23. ^{13}C-NMR spectrum (chloroform-d) of ferulic acid (**8**)

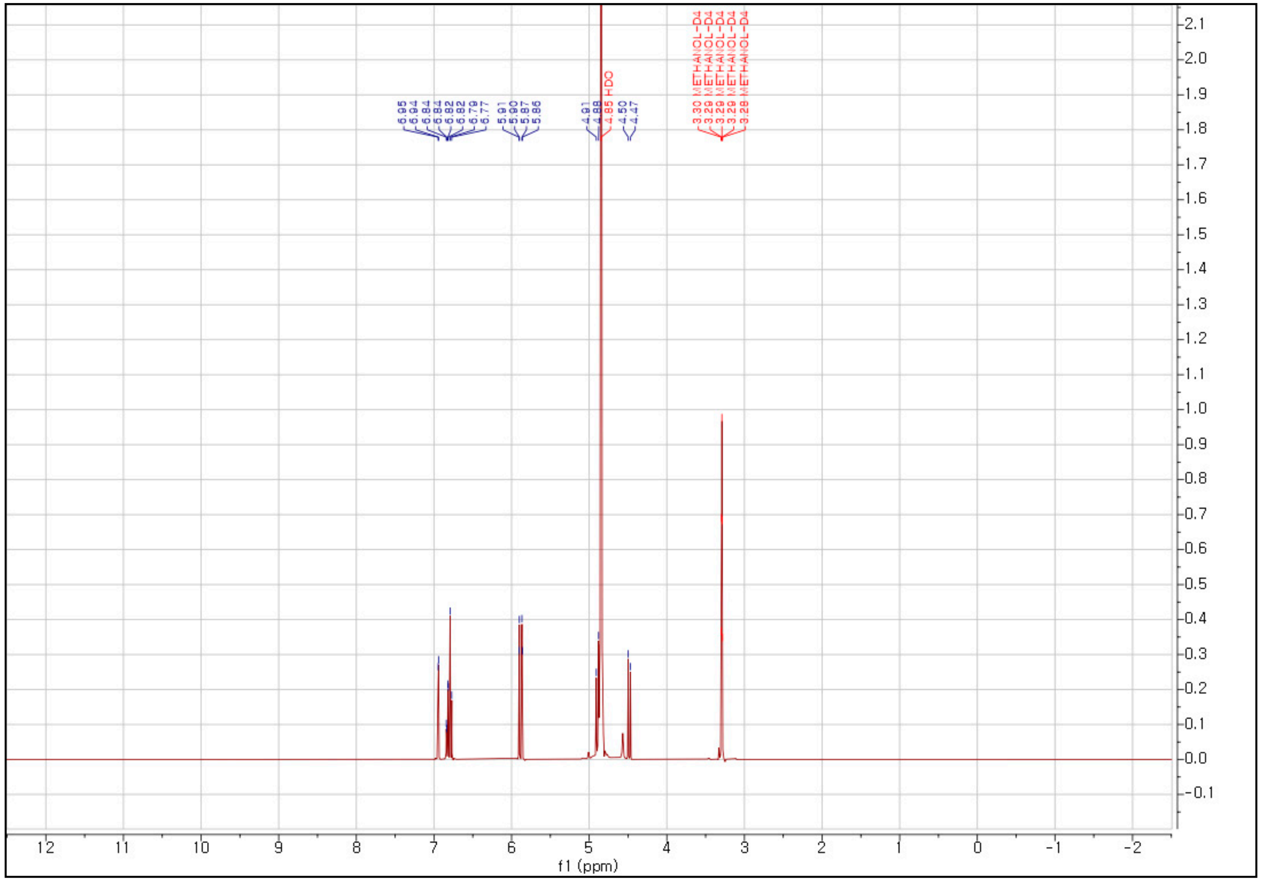

Figure S24. ^{1}H-NMR spectrum (methanol-d_4) of taxifolin (**9**)

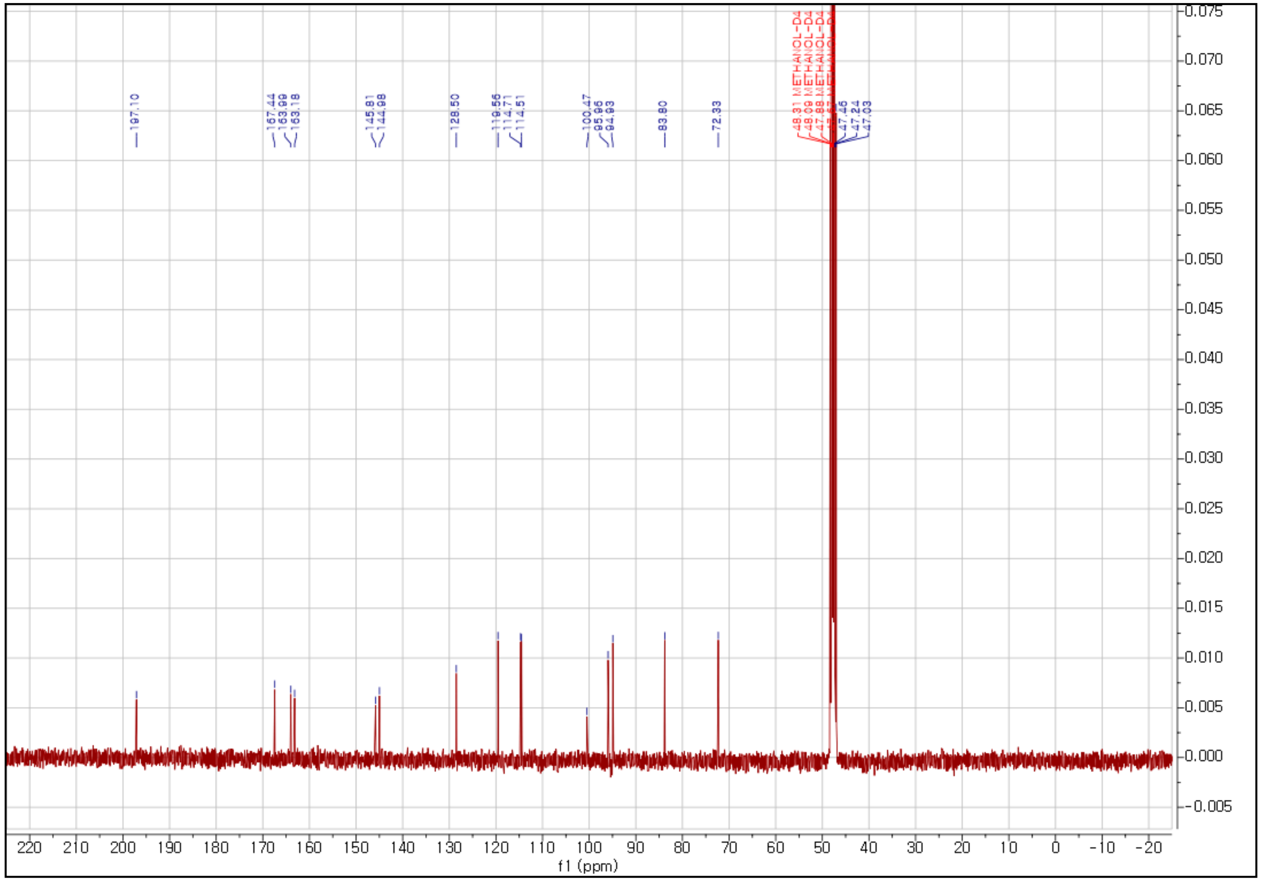

Figure S25. ^{13}C-NMR spectrum (methanol-d_4) of taxifolin (**9**)

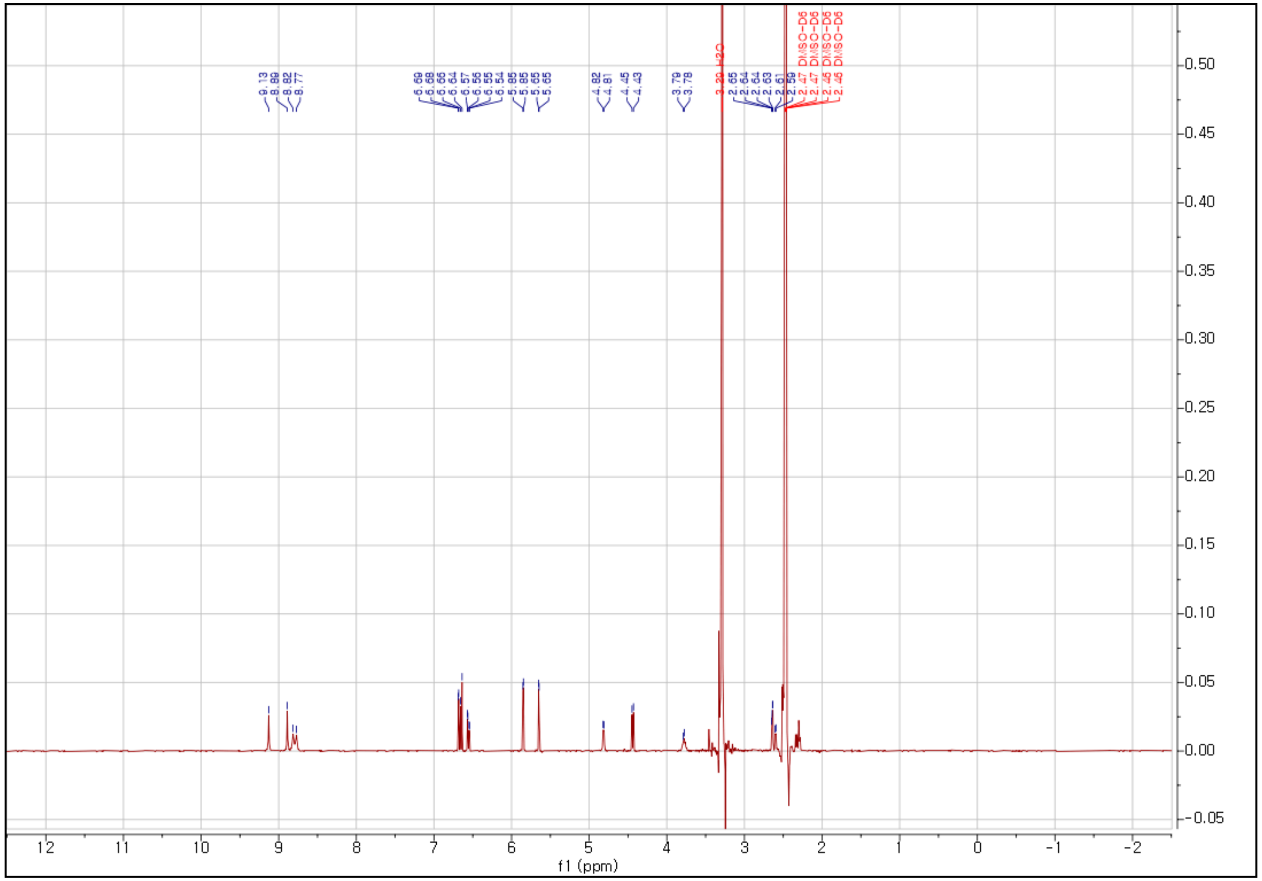

Figure S26. ^{1}H-NMR spectrum (DMSO-d_6) of catechin (**10**)

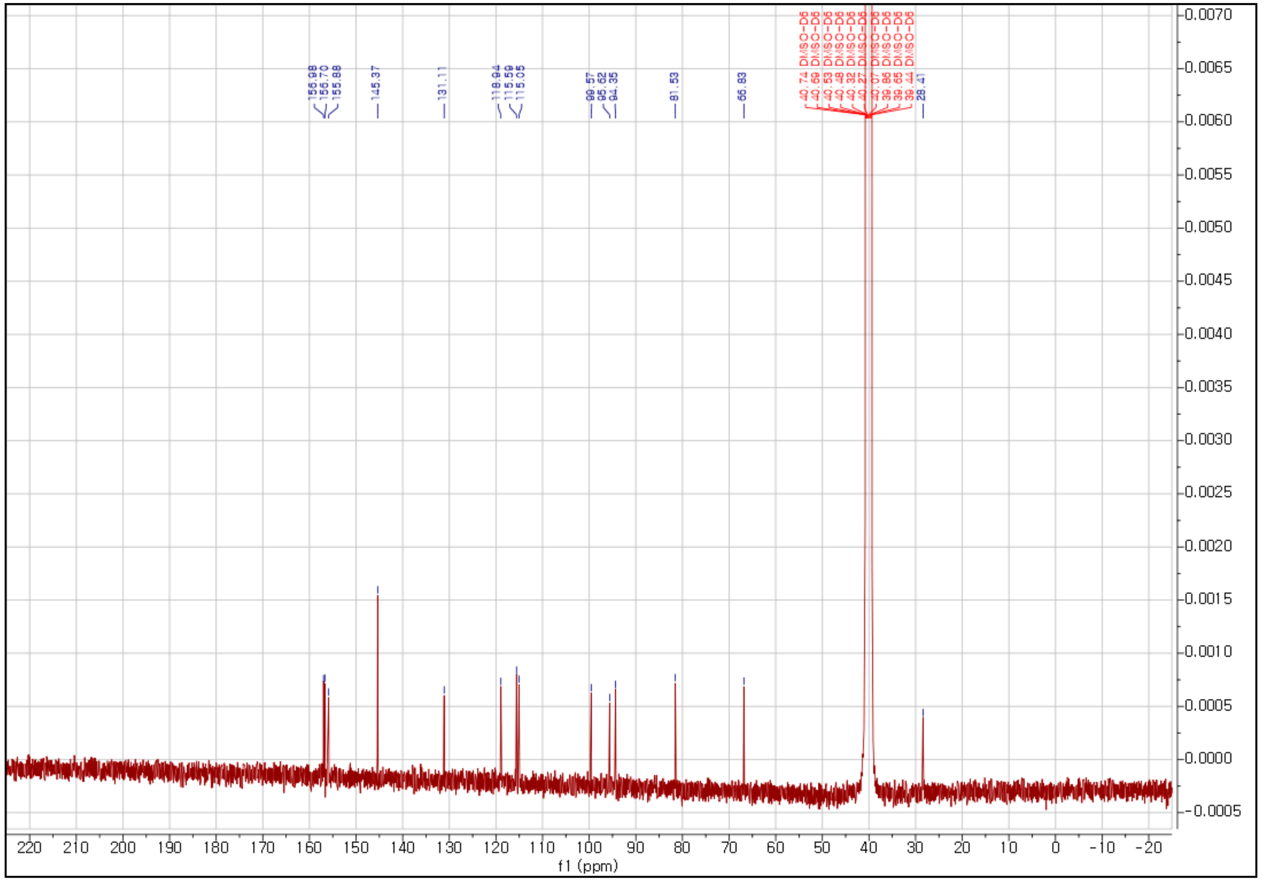

Figure S27. ^{13}C-NMR spectrum (DMSO-d_6) of catechin (**10**)

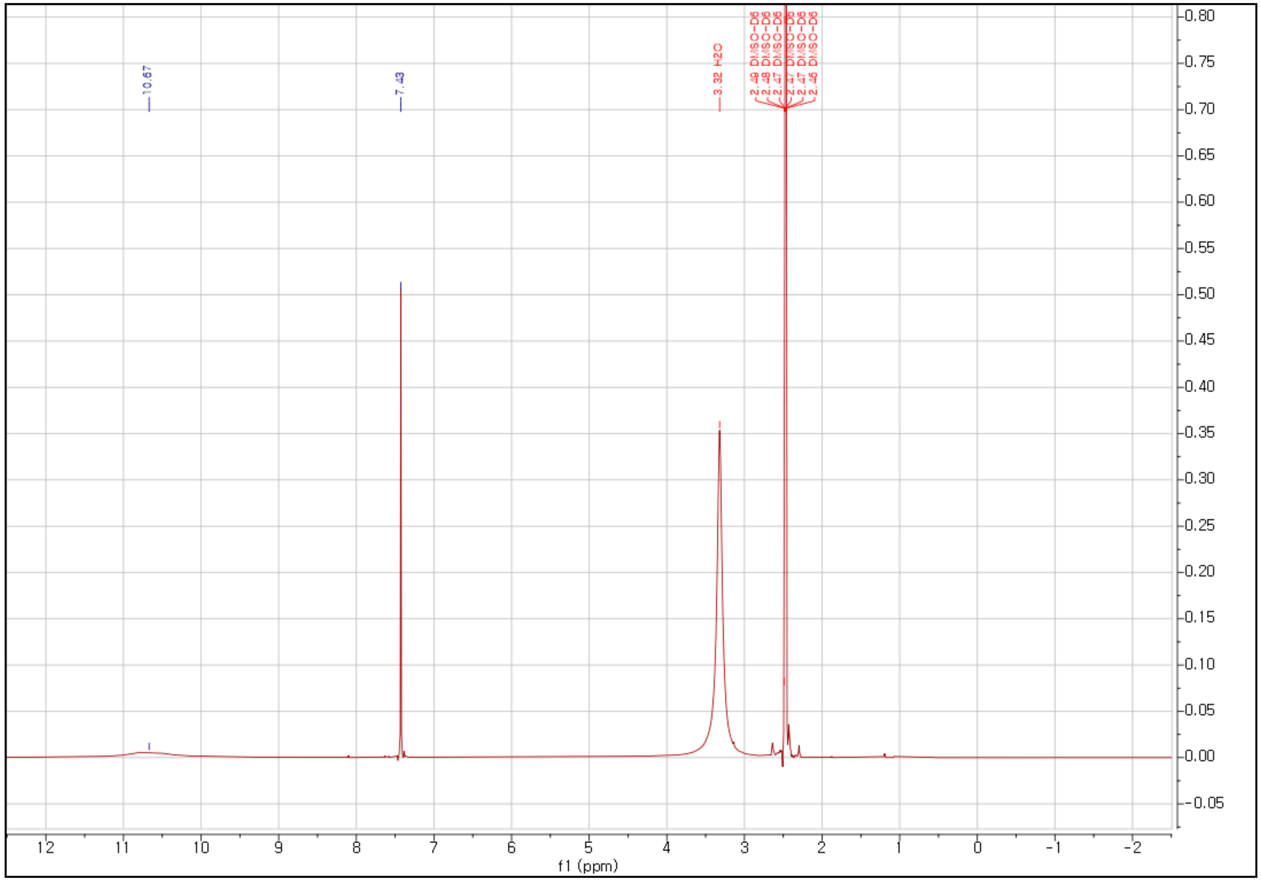

Figure S28. ^{1}H-NMR spectrum (DMSO-d_6) of ellagic acid (**11**)

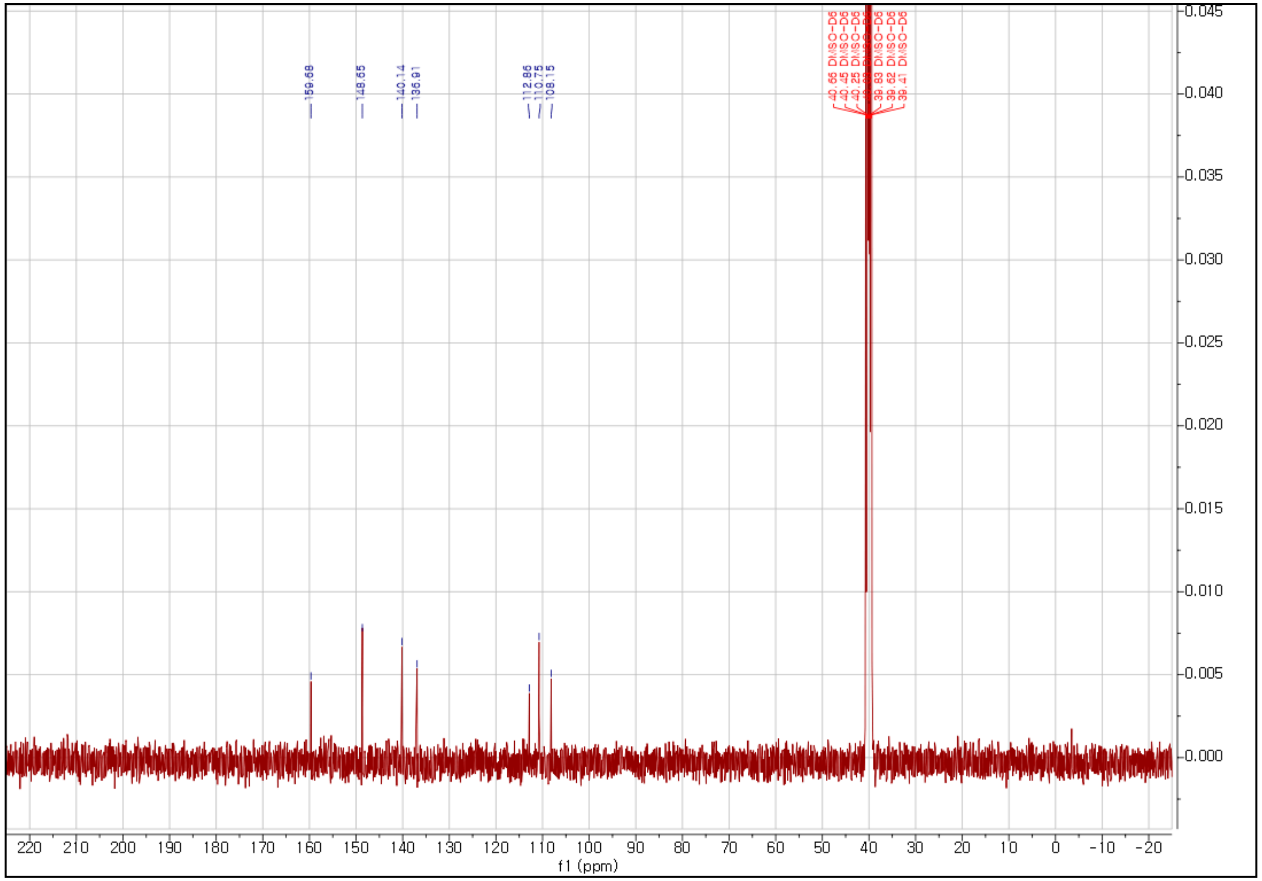

Figure S29. ¹³C-NMR spectrum (DMSO-d$_6$) of ellagic acid (**11**)

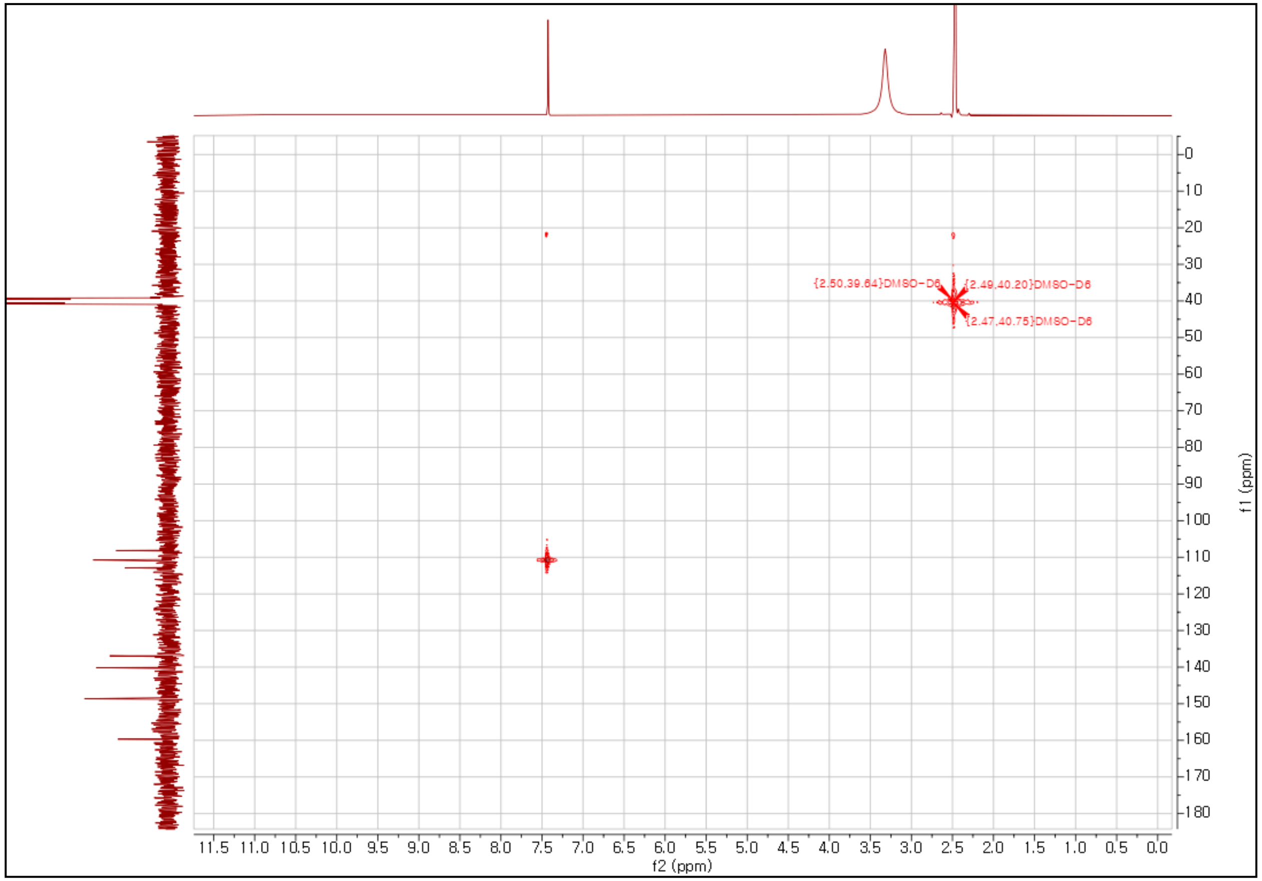

Figure S30. HMQC spectrum (DMSO-d$_6$) of ellagic acid (**11**)

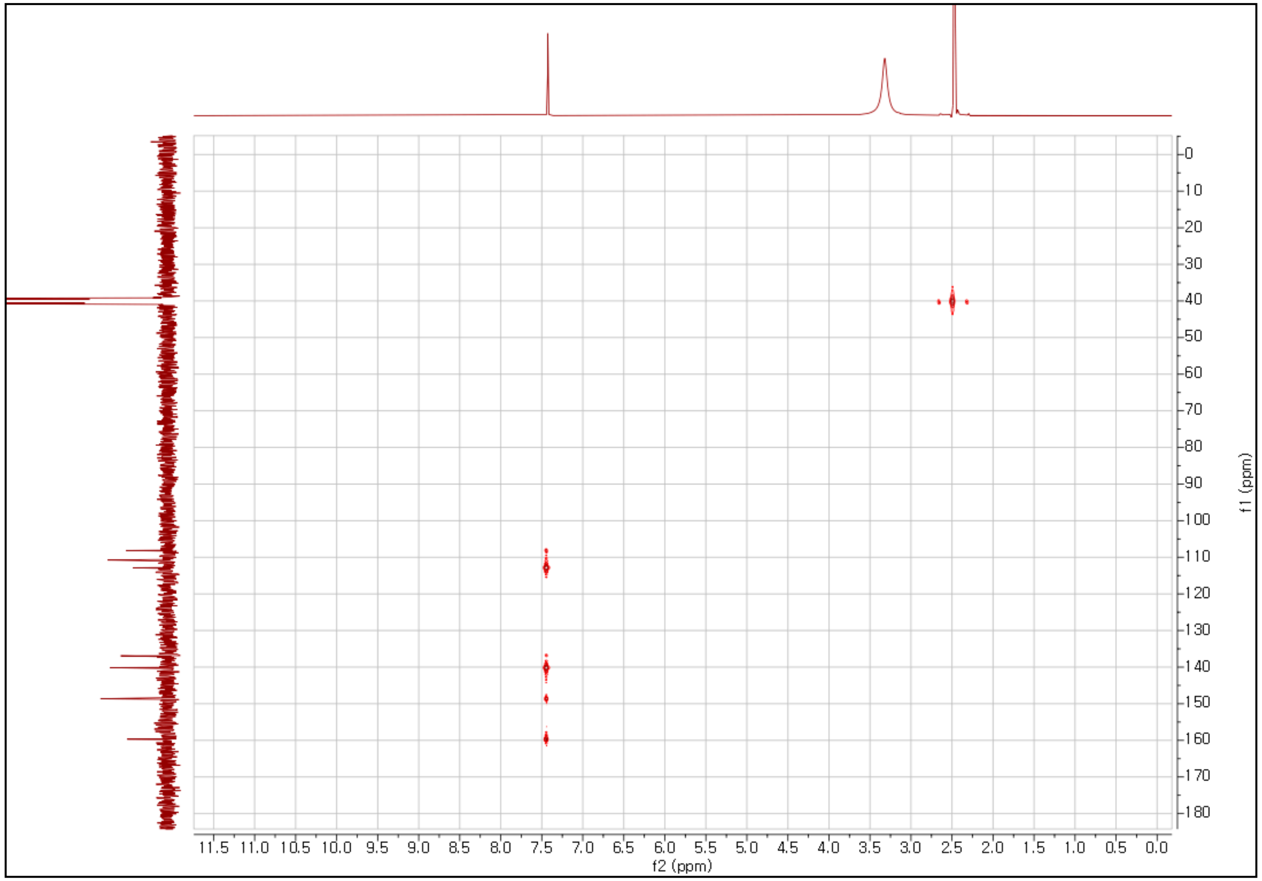

Figure S31. HMBC spectrum (DMSO-d6) of ellagic acid (**11**)

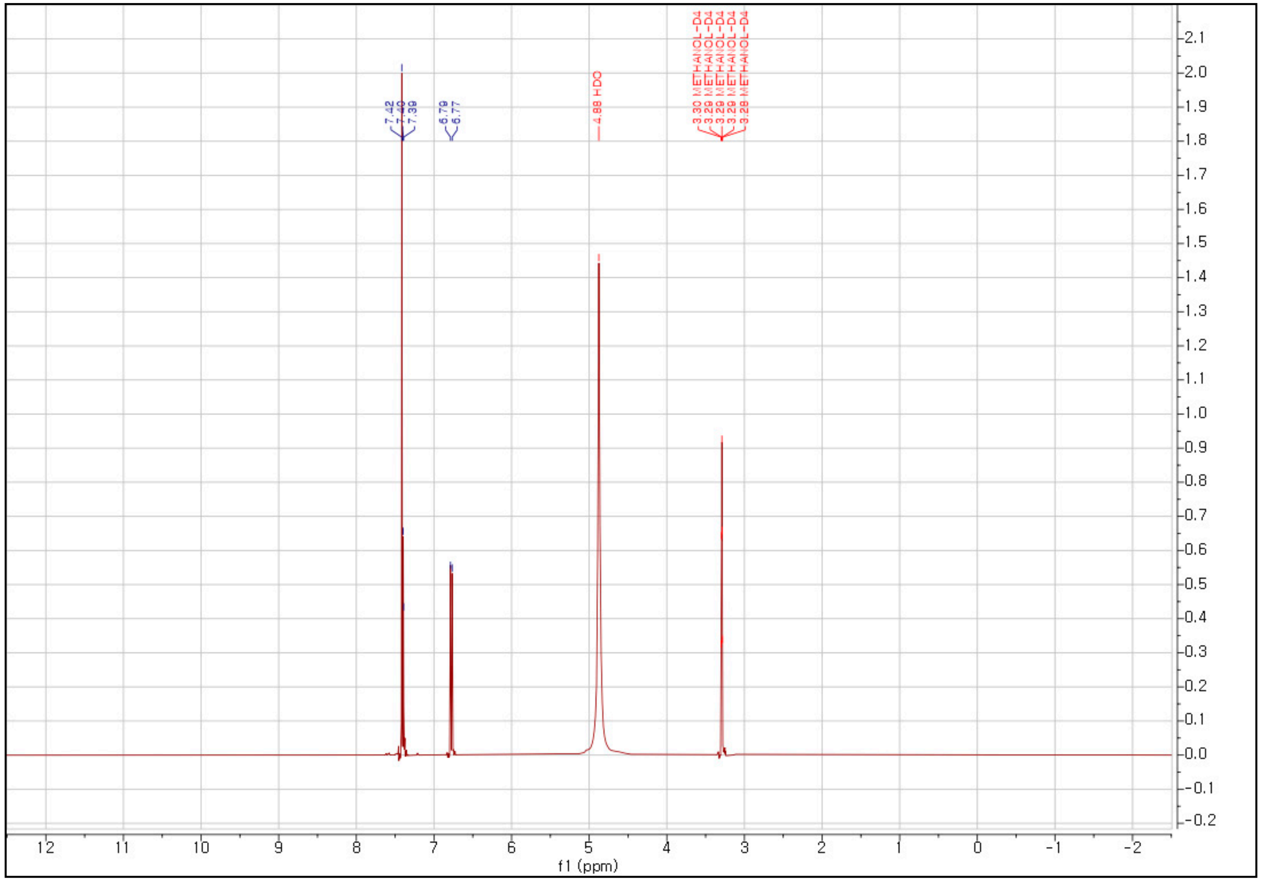

Figure S32. ^{1}H-NMR spectrum (methanol-d_4) of protocatechuic acid (**12**)

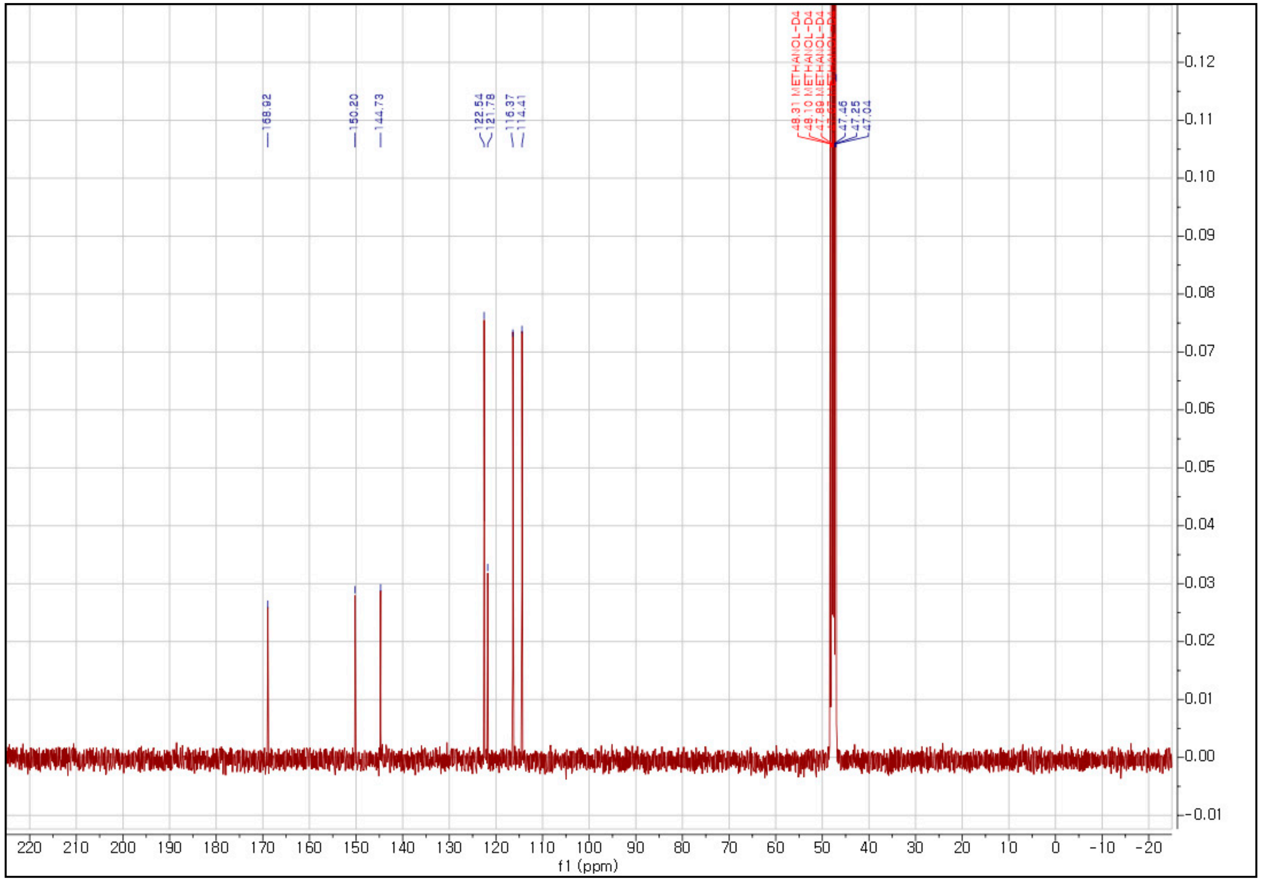

Figure S33. ^{13}C-NMR spectrum (methanol-d$_4$) of protocatechuic acid (**12**)

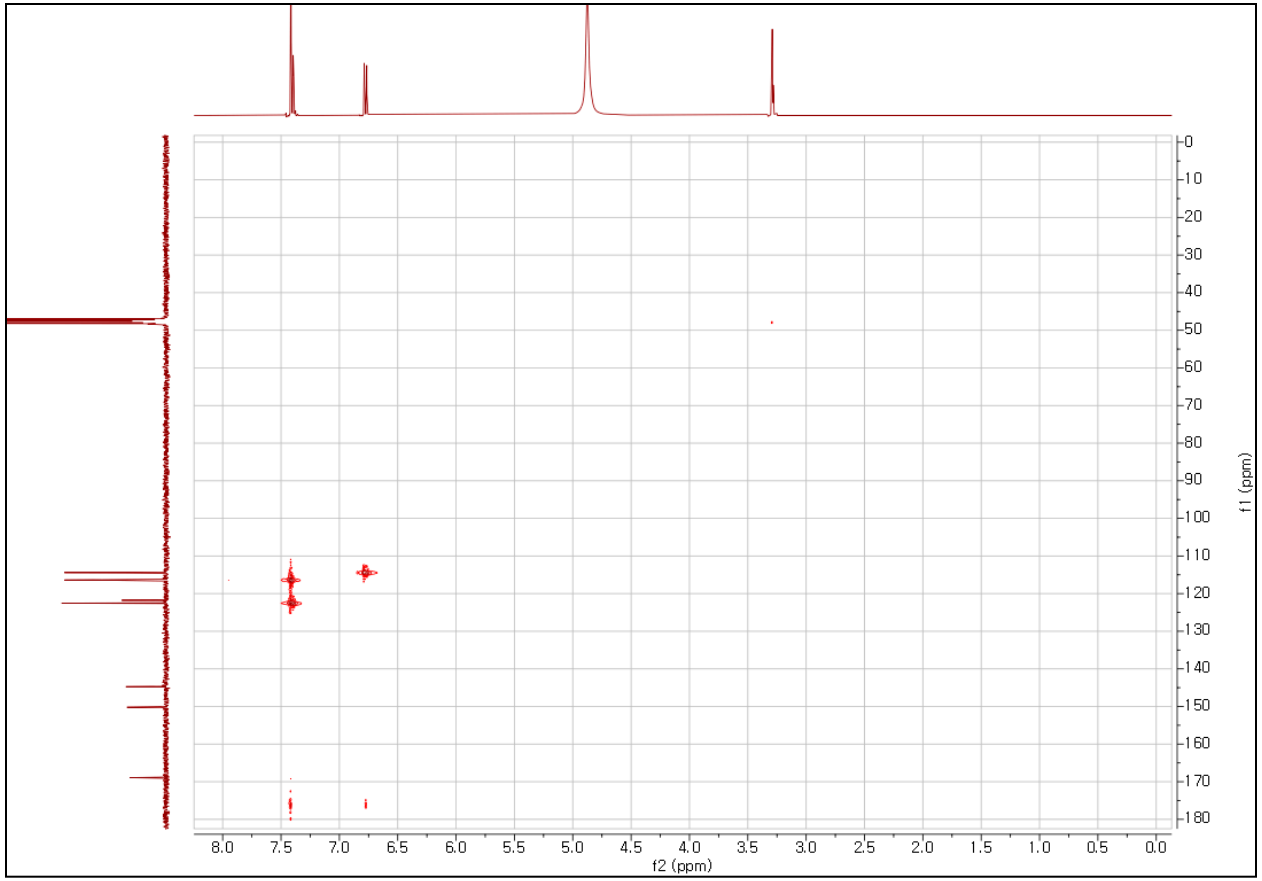

Figure S34. HMQC spectrum (methanol-d$_4$) of protocatechuic acid (**12**)

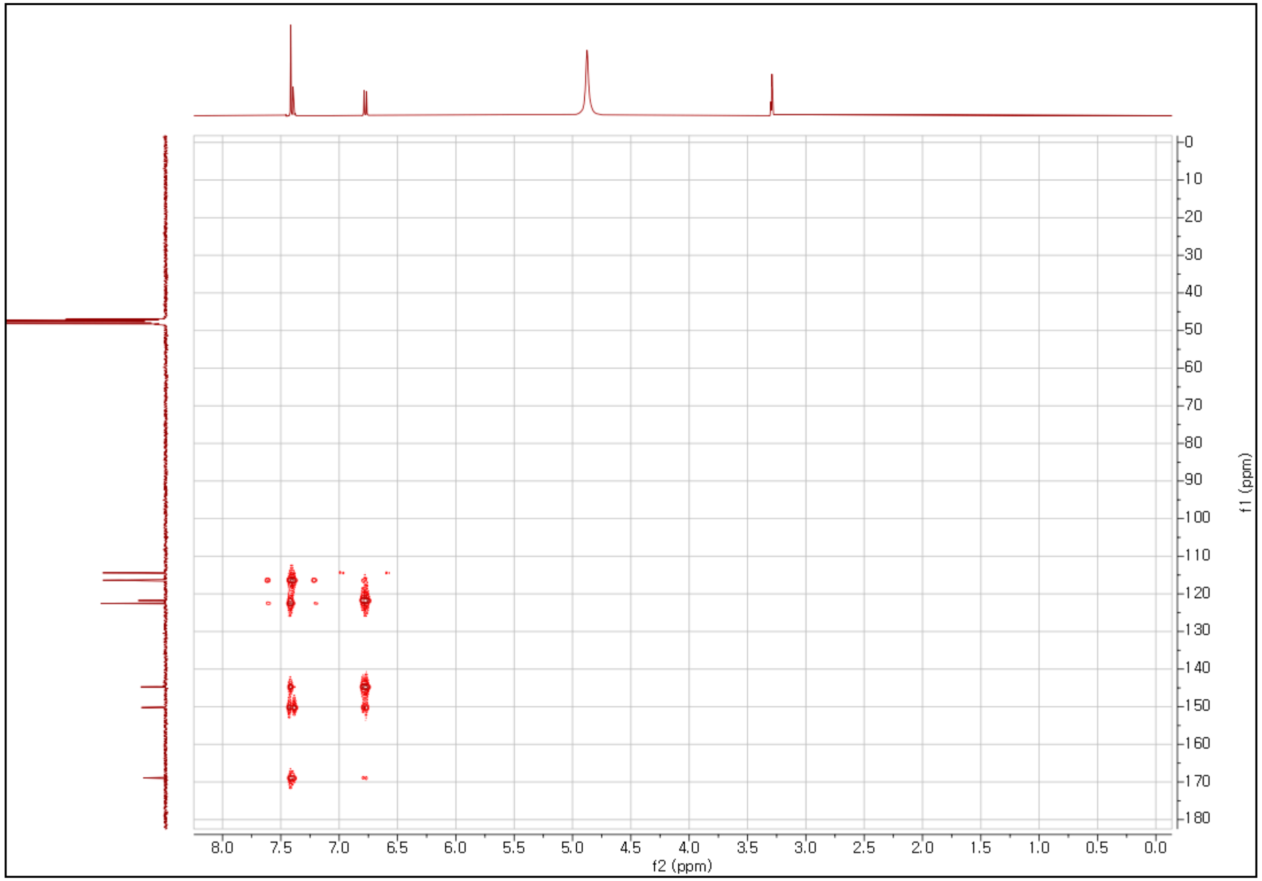

Figure S35. HMBC spectrum (methanol-d4) of protocatechuic acid (**12**)

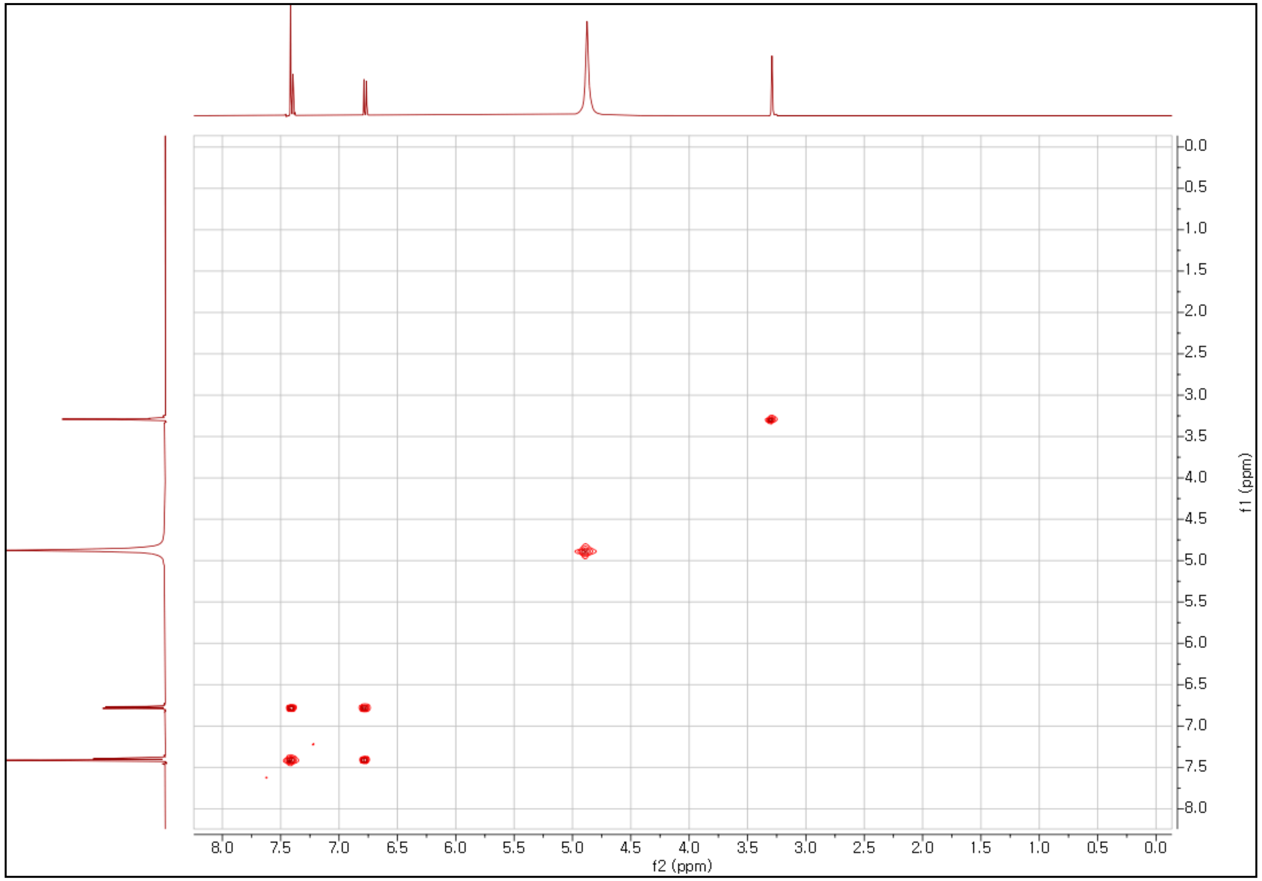

Figure S36. COSY spectrum (methanol-d4) of protocatechuic acid (**12**)

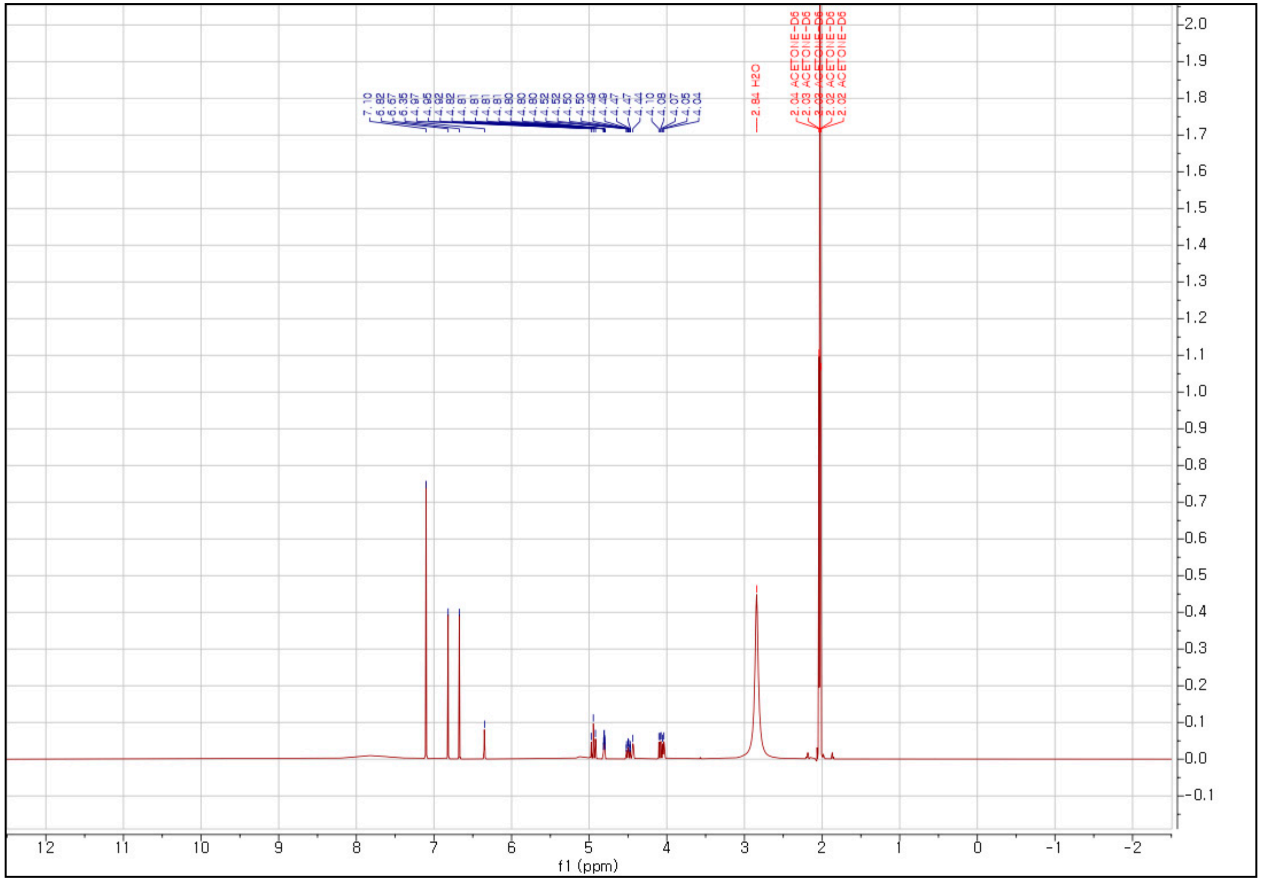

Figure S37. ^{1}H-NMR spectrum (acetone-d_6) of corilagin (**13**)

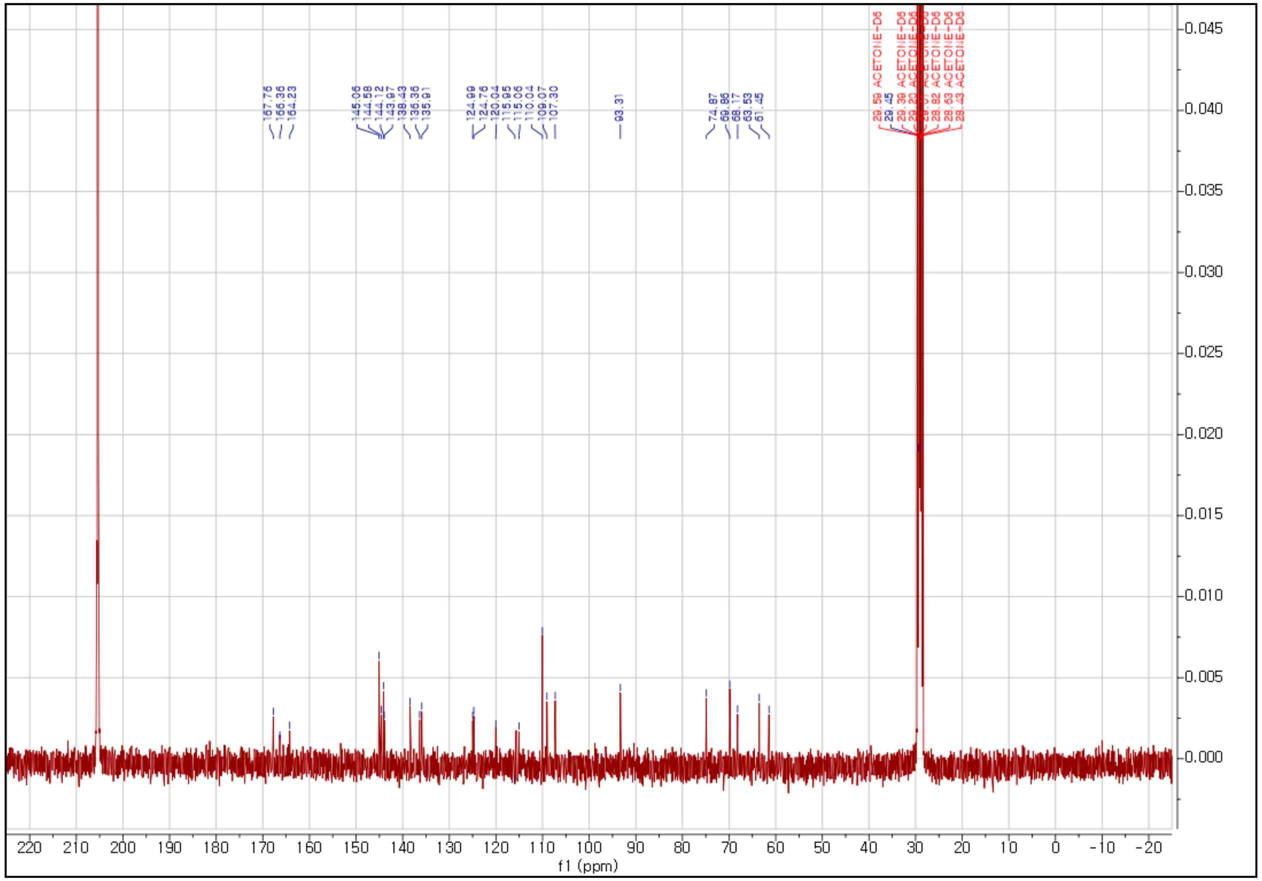

Figure S38. ^{13}C-NMR spectrum (acetone-d$_6$) of corilagin (**13**)

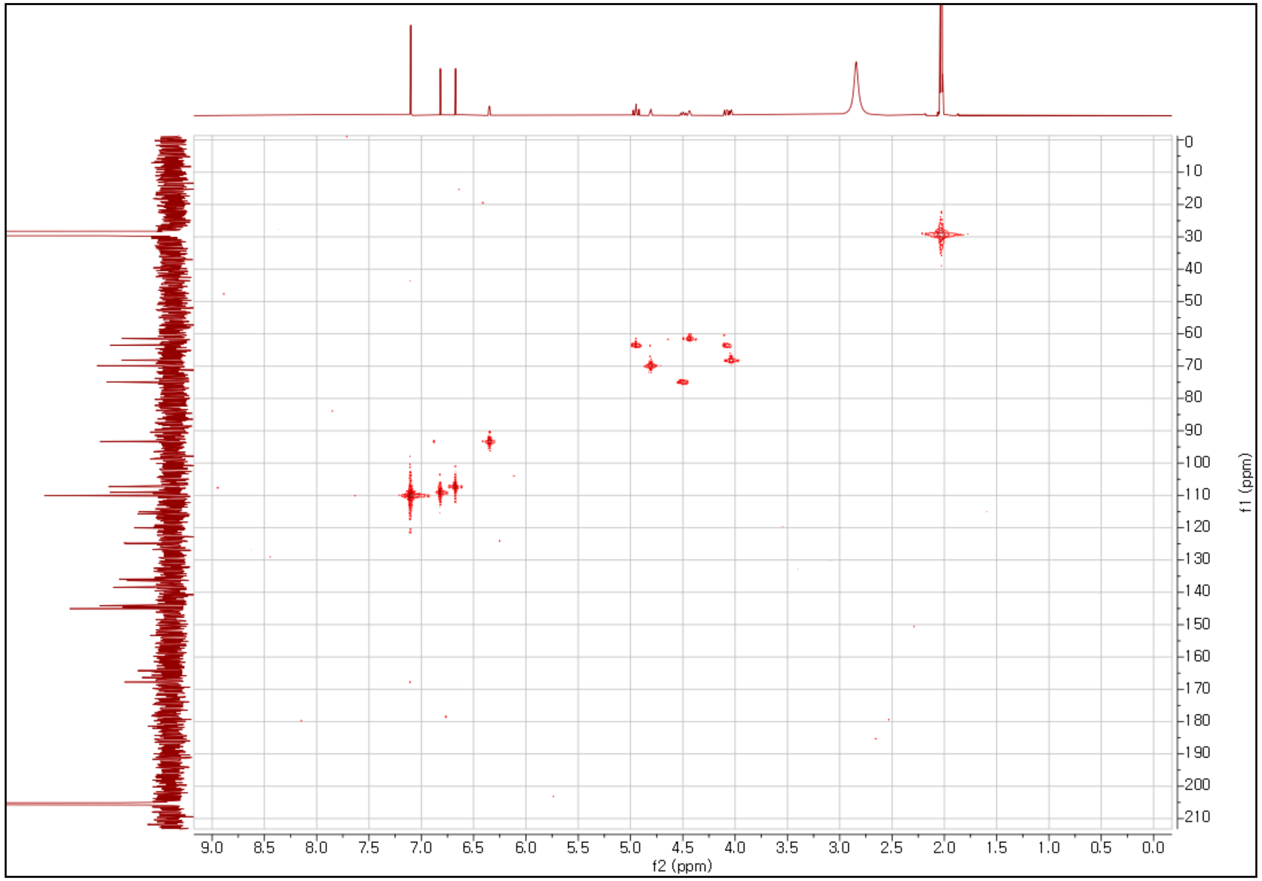

Figure S39. HMQC spectrum (acetone-d₆) of corilagin (**13**)

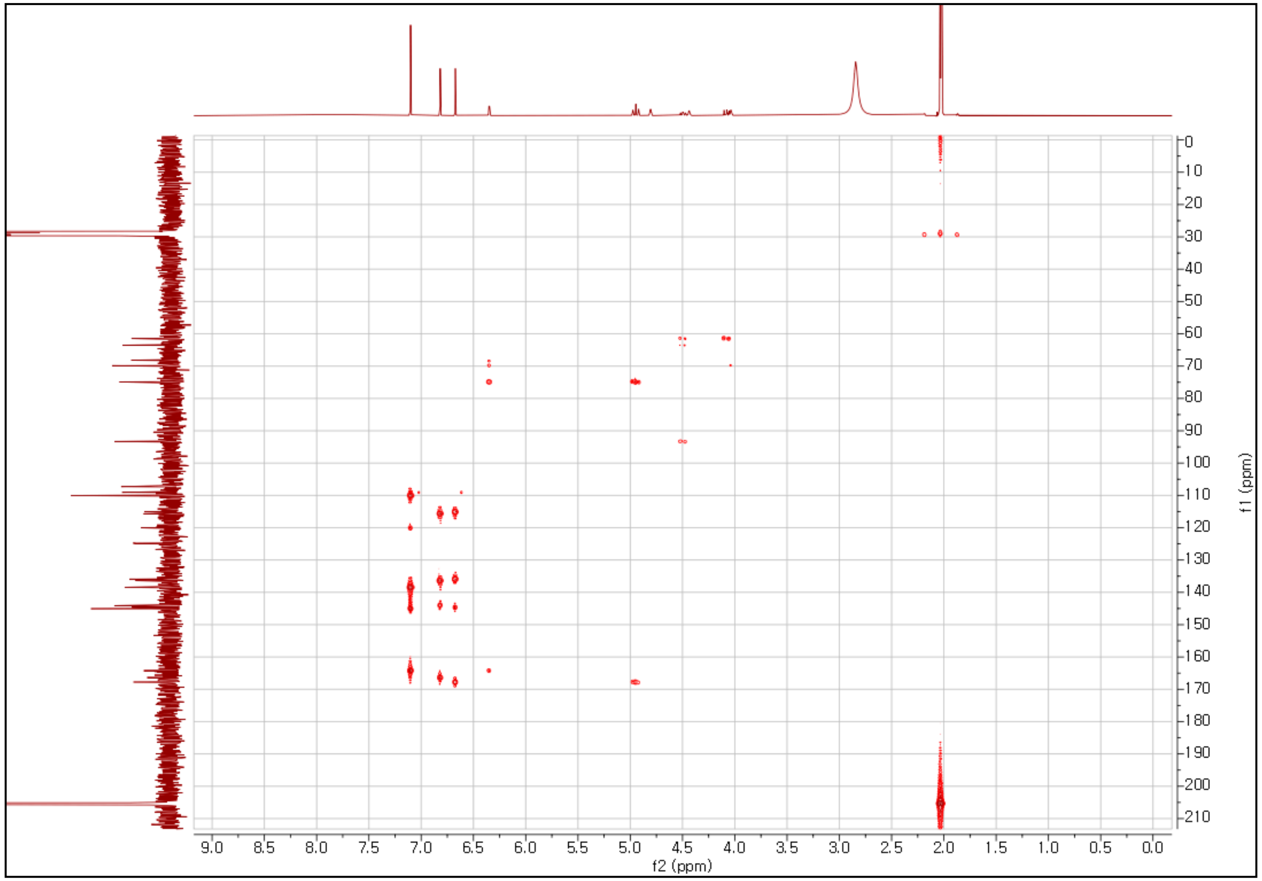

Figure S40. HMBC spectrum (acetone-d_6) of corilagin (**13**)

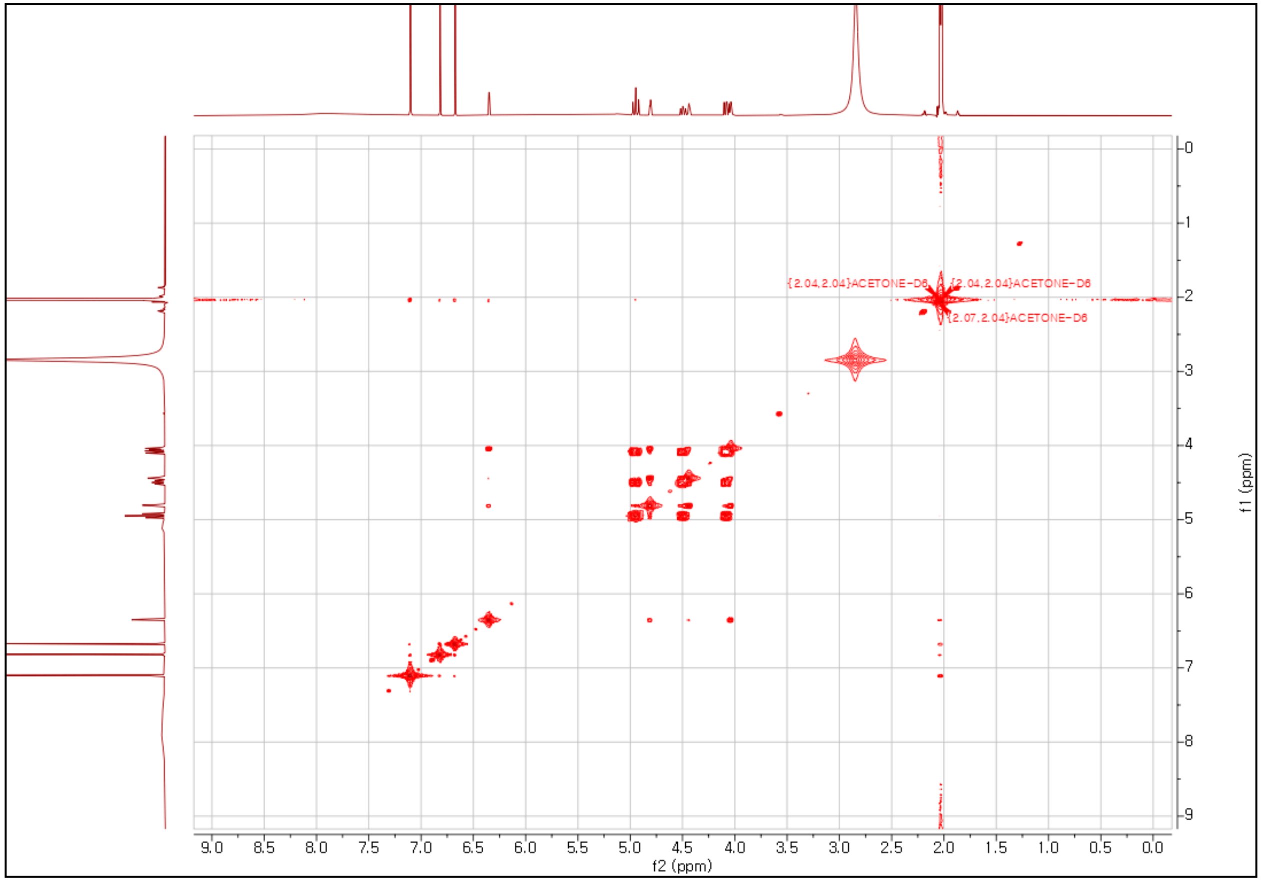

Figure S41. COSY spectrum (acetone-d_6) of corilagin (**13**)

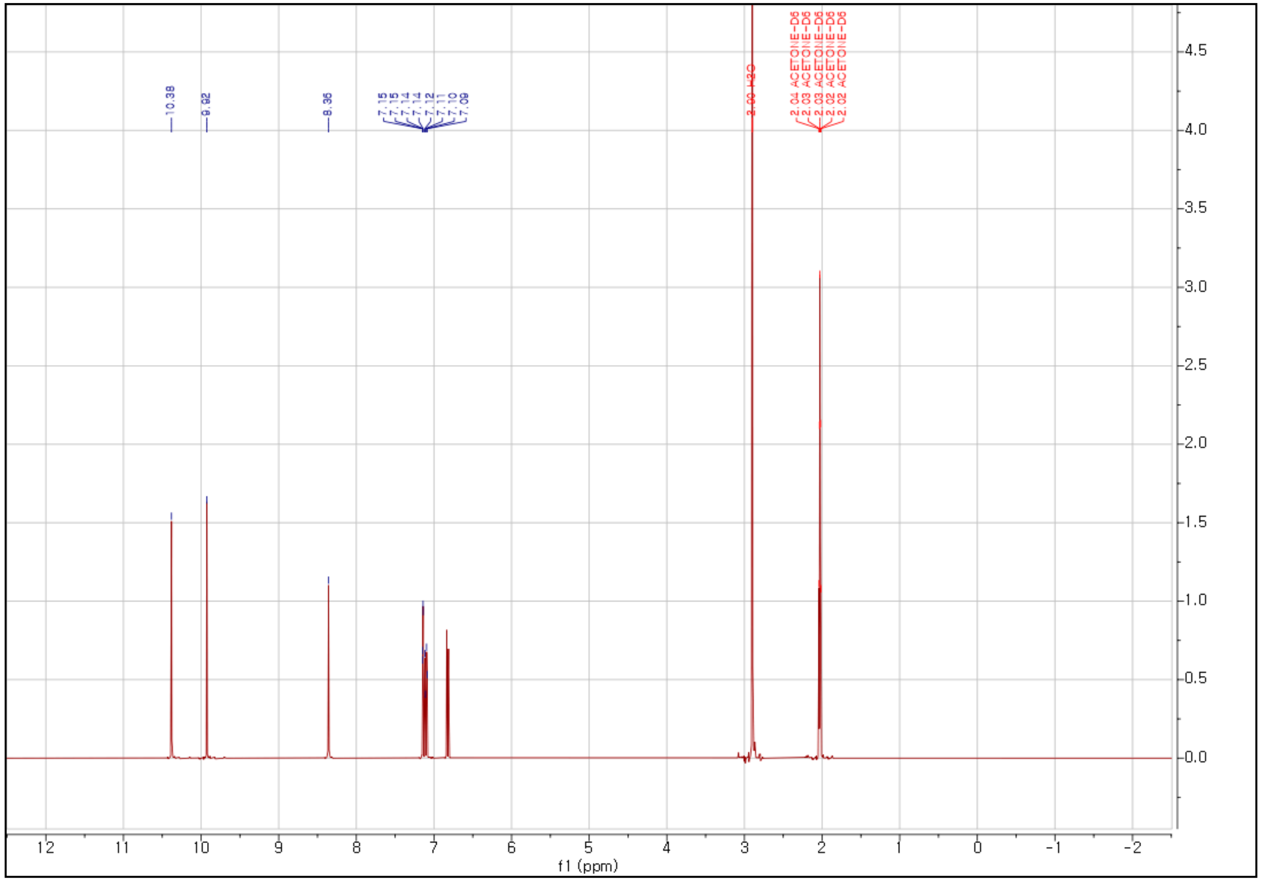

Figure S42. ^{1}H-NMR spectrum (acetone-d_6) of 2,5-dihydroxybenzaldehyde (**14**)

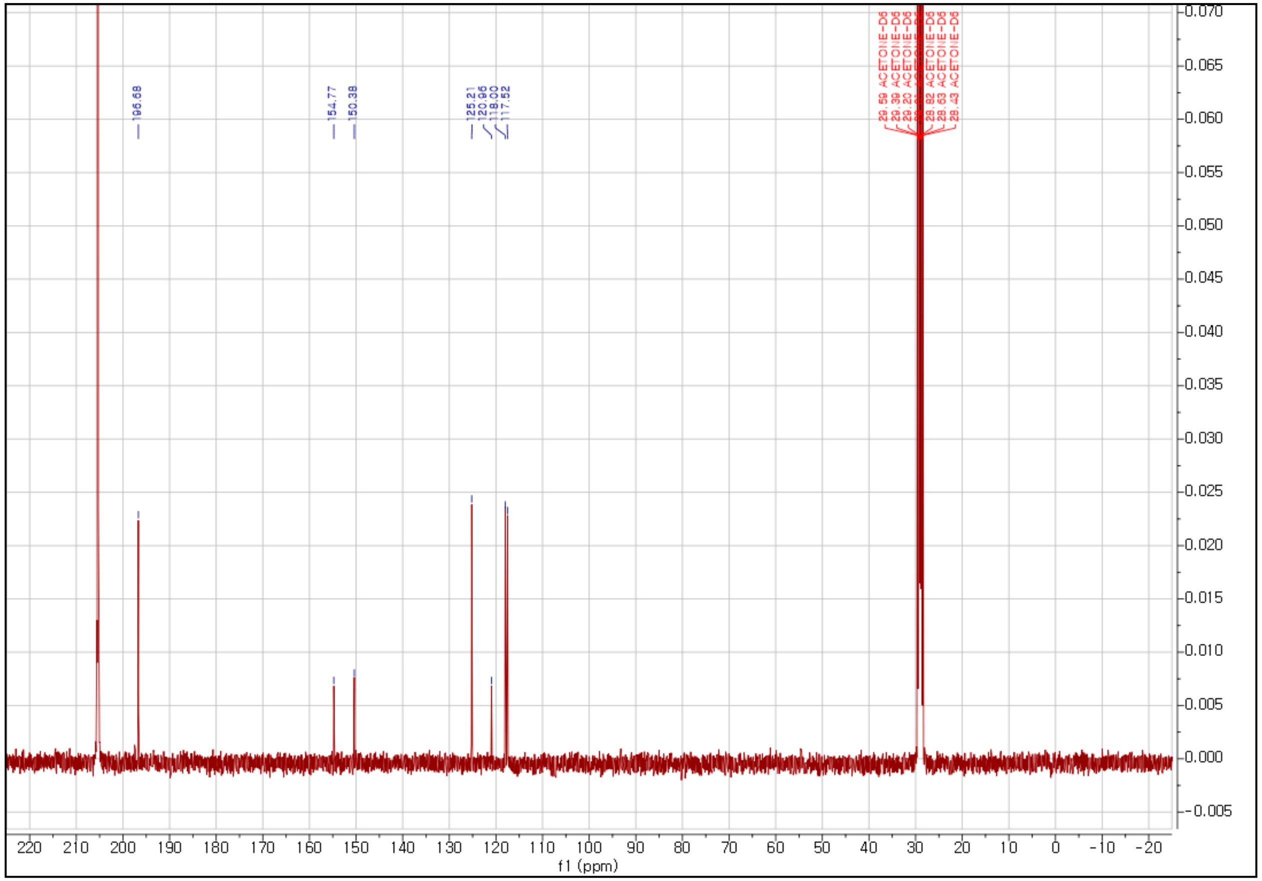

Figure S43. ^{13}C-NMR spectrum (acetone-d$_6$) of 2,5-dihydroxybenzaldehyde (**14**)

Gallic aicd (1)

purity 97.84 %

Sinapaldehyde (2)

purity 96.29 %

Coniferaldehyde (3)

purity 96.81 %

3,5,7,2',6'-pentahydroxyflavanone (4)

purity 95.57 %

1,2,3,6-Tetrakis-*O*-galloyl-beta-D-glucose (5)

purity 95.04 %

Phlorizin (6)

purity 97.97 %

3,4-Dimethoxycinnamic acid (7)

purity 99.54 %

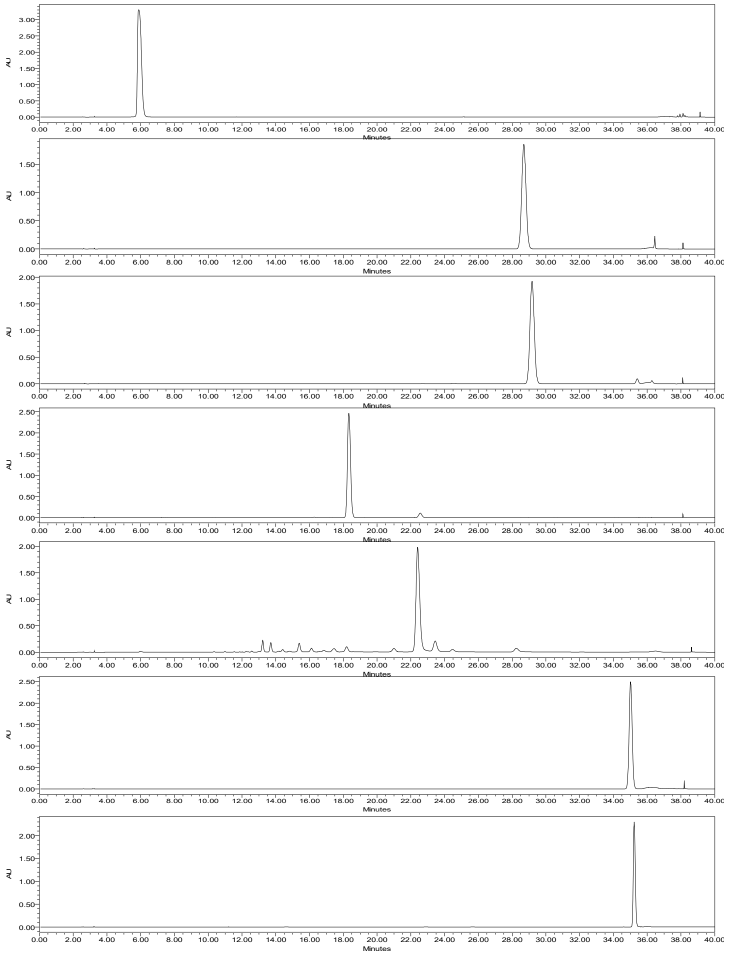

Figure S44. HPLC chromatograms of compounds **1**–**7** isolated from the ethanol extract of *Quercus acuta* fruits. Analyses were performed using an Agilent Eclipse XDB-C18 column (4.6 × 250 mm, 5 μm). The mobile phases consisted of solvent A (0.1% formic acid in water) and solvent B (acetonitrile) with the following gradient system: 0.0–5.0 min, 5% B; 5.0–20.0 min, 5–30% B; 20.0–28.0 min, 30–50% B; 28.0–32.0 min, 50–95% B; 32.0–36.0 min, 95% B; 36.0–40.0 min, 95–5% B. The flow rate was 1.0 mL/min, the injection volume was 10 μL, and detection was performed at 254 nm.

Ferulic acid (8)

purity 98.31 %

Taxifolin (9)

purity 95.48 %

Catechin (10)

purity 96.79 %

Ellagic acid (11)

purity 98.79 %

Protocatechuic acid (12)

purity 97.92 %

Corilagin (13)

purity 96.83 %

2,5-Dihydroxybenzaldehyde (14)

purity 96.07 %

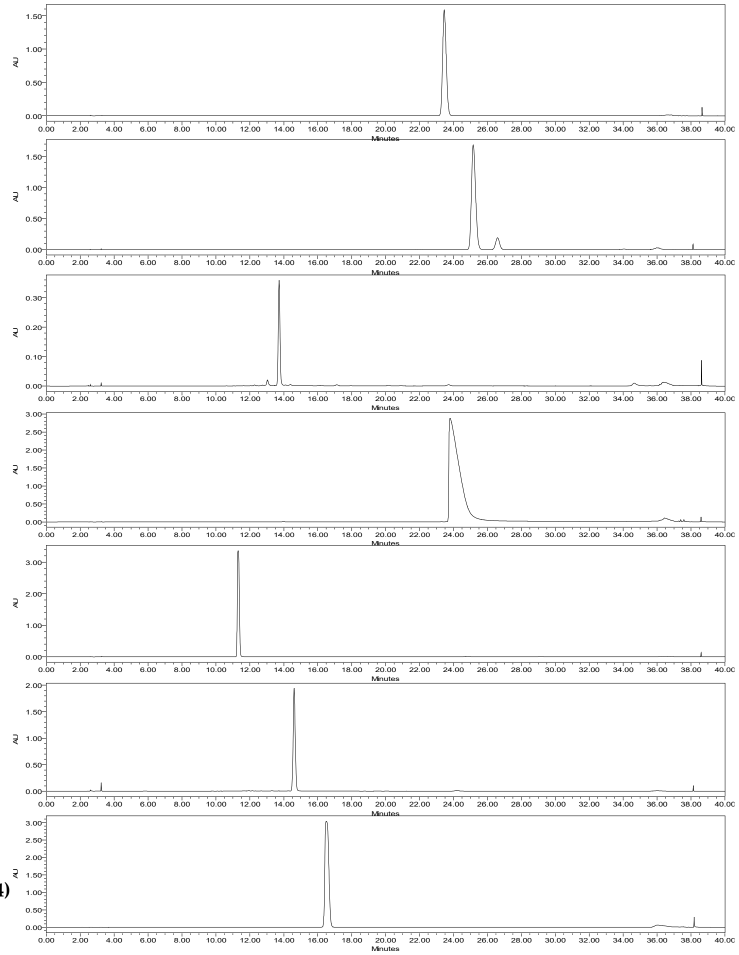

Figure S45. HPLC chromatograms of compounds **8**–**14** isolated from the ethanol extract of *Quercus acuta* fruits. Analyses were performed using an Agilent Eclipse XDB-C18 column (4.6 × 250 mm, 5 μm). The mobile phases consisted of solvent A (0.1% formic acid in water) and solvent B (acetonitrile) with the following gradient system: 0.0–5.0 min, 5% B; 5.0–20.0 min, 5–30% B; 20.0–28.0 min, 30–50% B; 28.0–32.0 min, 50–95% B; 32.0–36.0 min, 95% B; 36.0–40.0 min, 95–5% B. The flow rate was 1.0 mL/min, the injection volume was 10 μL, and detection was performed at 254 nm.
